# Structural and Functional Classification of G-Quadruplex Families within the Human Genome

**DOI:** 10.3390/genes14030645

**Published:** 2023-03-04

**Authors:** Aryan Neupane, Julia H. Chariker, Eric C. Rouchka

**Affiliations:** 1 School of Graduate and Interdisciplinary Studies, University of Louisville, Louisville, KY 40292, USA; 2 Department of Neuroscience Training, University of Louisville, Louisville, KY 40292, USA; 3 Kentucky IDeA Network of Biomedical Research Excellence (KY INBRE) Bioinformatics Core, University of Louisville, Louisville, KY 40292, USA; 4 Department of Biochemistry and Molecular Genetics, University of Louisville, Louisville, KY 40292, USA

**Keywords:** G-quadruplex, G4, clustering, hidden Markov models, DNA structures

## Abstract

G-quadruplexes (G4s) are short secondary DNA structures located throughout genomic DNA and transcribed RNA. Although G4 structures have been shown to form in vivo, no current search tools that examine these structures based on previously identified G-quadruplexes and filter them based on similar sequence, structure, and thermodynamic properties are known to exist. We present a framework for clustering G-quadruplex sequences into families using the CD-HIT, MeShClust, and DNACLUST methods along with a combination of Starcode and BLAST. Utilizing this framework to filter and annotate clusters, 95 families of G-quadruplex sequences were identified within the human genome. Profiles for each family were created using hidden Markov models to allow for the identification of additional family members and generate homology probability scores. The thermodynamic folding energy properties, functional annotation of genes associated with the sequences, scores from different prediction algorithms, and transcription factor binding motifs within a family were used to annotate and compare the diversity within and across clusters. The resulting set of G-quadruplex families can be used to further understand how different regions of the genome are regulated by factors targeting specific structures common to members of a specific cluster.

## 1. Introduction

**Figure 1:**
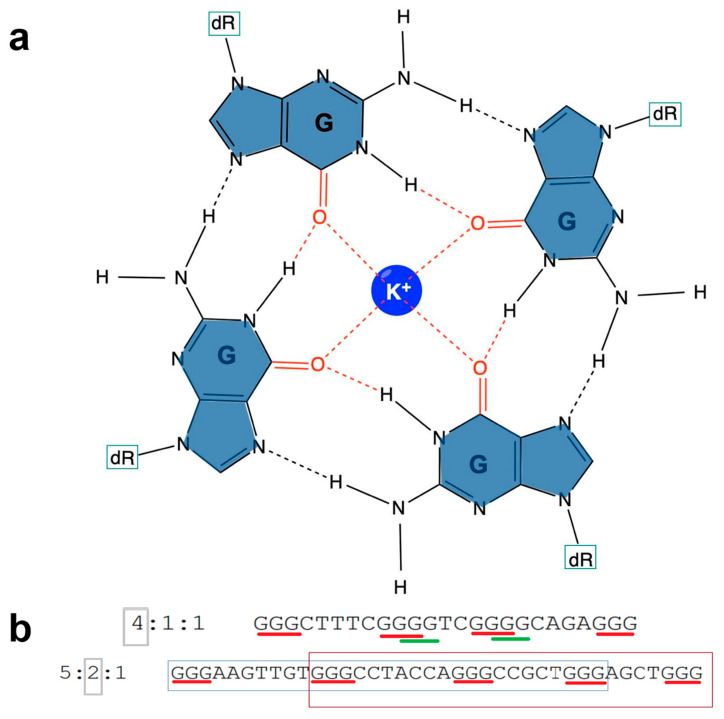
G-quadruplex structure. (**a**) G-tetrad structure that forms G-quadruplexes. Hydrogen bonds between the guanine from different tetrads form a planar ring. (**b**) Sequence of G4 with multiple guanine tetrads. Here, 4:1:1 and 5:2:1 refer to the result from Quadparser separated out as the number of tetrads: total G4 sequences: non-overlapping G4 sequences. Red line: first occurrence of G4 forming sequence; green line: alternative G4 forming sequence.

G-quadruplexes are stranded secondary structures of nucleic acids rich in guanine with the most common form containing four runs of at least three guanines. These runs are separated by short loops, typically 2–7 nucleotides in length, which can potentially fold into an intramolecular G-quadruplex structure. The tetrad guanine structure is stacked on top of each other and held together by mixed loops of DNA forming Hoogsteen base pairing giving a four-stranded structure with nucleobases on the inside and a sugar-phosphate backbone on the outside (Figure 1a,b). Metal ions (typically K^+^ or Na^+^) sitting internally to the Hoogsteen bases stabilize the base pairing. Stacking occurs through the O-6 atoms of guanines facing the center creating a tubular space able to function as an ion channel. The presence of a metal cation in this channel allows for interaction with the eight O-6 atoms of the guanine quartet. While the triplet tetrad is most frequently observed, two tetrad structures have been experimentally shown to form G-quadruplexes as well [1].

### 1.1. Roles of G-quadruplexes

Over the past three decades, guanine-rich quadruplex sequences have been implicated as key structural regulators of gene expression, cellular differentiation, transcription factors, and their cell line and tissue specificity [2]. Similarly, elevated levels of G-quadruplexes have been identified across cancer tissues including breast [3], stomach [4], and liver cancer [5] as well as neurodegenerative diseases [6]. Computational analyses of G-quadruplex patterns have identified the prevalence of G-quadruplexes in oncogenic promoters, introns, splice sites, and intergenic and telomeric ends. Initially, the secondary structures were thought to act as a physical obstacle to RNA polymerase for transcription, as identified through G4-specific antibodies [7,8] and chemical probing [9,10]. Further evidence suggests that the varied tissue-specific functionality of these structures is affected by the crosstalk of additional transcription factors [11], proteins, and physiological conditions. Additionally, G4 structures have a role in genomic instability and are associated with higher rates of double-strand breakage in nucleosome-depleted regions of highly expressed cancer genes. High- and low-density bands of G4 across both chromosomal strands have been observed showcasing a role of G-quadruplex in the pairing of homologous chromosomes during meiosis [12]. Further, recent evidence shows that G4 formation is highest during DNA replication in the S phase and lowest during the G2 and M phases, which is consistent with phases of transcription, replication, and chromatin accessibility [13].

### 1.2. Characteristics of G4s

Sequence characteristics such as sequence length [14], base composition [15,16], and loop length [17,18,19,20,21] are important parameters for defining the secondary structure and stability of G-quadruplexes. Molecular dynamics show that telomeric G4 repeats (TTAGGG) in the presence of a K^+^ cation form a structure with three single nucleotide loops in a parallel fashion. Increasing the loop length by a single base causes the sequences to adopt a mixture of parallel and antiparallel folded structures [22]. The conformation and stability of G-quadruplexes have been used to study to effect of transcription factor binding and altered mRNA expression of several genes. Examples include nucleolin [23] and Ewing’s Sarcoma proteins [24] which preferentially bind to structures with longer loop lengths. Computationally, G4s are defined by the pattern G*_x_*N_1–7_G*_x_*N_1–7_G*_x_*N_1–7_G*_x_* where x ≥ 3 (length of guanine repeats). The guanine tracts are separated by loops of any base composition of length 1–7 bases. This pattern is the basis for regular expression-based tools such as Quadparser [25] and QGRSmapper [26]. With experimental data, it is known that different intermolecular structures, long loops, and non-canonical structures with G tracts containing two guanines exist [1,27,28,29]. Methods such as G4screener [30], PQSfinder [31], and G4Catchall [32] allow the search of G-quadruplexes for variable quartet and larger loop sequences. G4Hunter [33] provides a score for guanine skewness which is based on predefined values, with a score based on the number of consecutive Gs. G4RNAscreener [30] uses a machine learning algorithm trained with experimental RNA sequences from the G4RNA database [34] and incorporates a threshold using metrics from tools such as G4Hunter [33], cG/cCscore, and G4 Neural Network score for G4 prediction. RNAfold [35] has an option to predict the thermodynamic parameters for G-quadruplex formation. DSSR [36] and ElTetrado [37] use the tertiary structure of each G-quadruplex for annotating and classifying different base pairs and tetrad structures. Notably, 3D-NuS [38] allows visualization of 3D DNA structures including duplex, triplex, and quadruplexes. Notably, 3D NuS visualizes the G-quadruplex structure and its strand orientation, loops and G quartets based on the energy minimization of G4 structures using experimental data. 

The G4 structure was found to be evolutionarily conserved in seven yeast species [39]. While G-quadruplex regions are significantly enriched in regulatory regions of eukaryotes, short loops of G4 are conserved in different species. Protozoa and fungi have limited diversity of G4 while an increase in diversity has been observed across invertebrates and vertebrates [40]. However, the evolutionary mechanism for this structure or the relationship of these structures at an evolutionary scale is not known.

Sequencing read fragments utilizing a customized approach that introduces stabilizing and destabilizing conditions (K^+^, Li^+^, pyridostatin) allows for high throughput sequencing of G4 locations [41,42] with a method known as G4 seq. Versions of this method have been used to identify 1,420,841 G-quadruplexes in 12 species. Using a similar method, 161 and 168 G4 sites were identified in the genomes of *Pseudomonas* [43] and *Escherichia* [44], respectively.

Over 100,000 G4 sequences have been mapped in vivo to the human genome. Several proteins such as FUS, TAF15, TARDBP, and PCBP1 have been determined to be enriched at G4 loci using artificial G4 binding [45]. SP2, a transcription factor (TF) encoded by a subfamily of the Sp/XKLF family, is a sequence-specific TF that has a strong association with G-quadruplex affinity. SP2 binds to the CCAAT motif independent of the zinc finger domain necessary for binding to GC-rich motifs [46]. It was shown in vitro that the SP1 TF was able to bind to a DNA sequence lacking the consensus motif and was able to form G-quadruplex sequences [47]. Luciferase expression studies show sequences of G4s in the KIT promoter mutated through site-directed mutagenesis were able to create a modulation (on/off) system for KIT expression through SP1 binding [48]. Additionally, G-quadruplex structures can bind to G-quadruplex sites in other promoter locations [49] mediating cis [50] and trans [51] acting regulation of transcriptional and translational processes, respectively, implying that G-quadruplex sequence and structural diversity are key factors for biological functions. 

### 1.3. G4 Families

Previously, a small family of G-quadruplexes labeled Pu27 was identified based on sequence homology [49]. The parent G-quadruplex is a 27 nucleotide (nt) G4 formed in the nuclease hypersensitive element (NHE) region of the *c-MYC* promoter associated with different forms of cancer, and predominantly involved in the regulation of expression of the *c-MYC* gene [52]. *c-MYC* is an oncogene that regulates genes in the cell cycle and molecular metabolism. Rezzoug et al. identified seventeen potential G-quadruplex forming sequences homologous to the Pu27 G4 which have been shown to bind to the NHE region of the c-MYC promoter selectively [49]. In addition, G4 regions regulating *VEGF* genes have been shown to have an additional G-tract to act as a spare tire for the formation of the G-quadruplex sequence of oxidative damage to the guanine tracts [53]. Similar sequences have been identified for *c-MYC*, *KRAS* [54], *BCL2* [55], *HIF-1α*, and *RET* genes. This highlights the presence of sequence-specific G-quadruplexes able to form, bind and regulate gene expression. Further, over the past decade, numerous G-quadruplex stabilizing and destabilizing ligands have been identified that recognize and interact selectively with these G4 sequences. Different classes of these heteroaromatic polycyclic, macrocyclic, and aromatic compounds have been designed to target the diversity of the G4 structure. The subtle differences in grooves, loop composition, and loop length allow for structural variability in these sequences. DNA aptamers that can form G4 are used for binding nucleolin [56]. More than 50 transcription factors with overlapping binding sites to the G4 region have been identified [2,57]. The folding, misfolding, and unfolding of G4 structures have been implicated in different biological processes [58,59].

### 1.4. Detection of G4 Families

The prediction of G-quadruplexes across genomes can be useful to identify the location of similarly structured G-quadruplexes, which can in turn be used to develop profiles of independent families based on the conservation of a variety of factors. We present a framework for predicting G-quadruplex sequences and identify similar sequences using trained profile hidden Markov models (HMMs) [60]. We identify pG4 sequences across the human genome and cluster these sequences using sequence clustering tools, CD-hit [61], MeShClust [62], and DNACLUST [63] as well as starcode [64] and BLAST [65]. These approaches utilize average weighted clustering to identify the quartet and loop patterns. We then further train HMM models using these clusters for the creation of families. Despite the short length of G-quadruplex sequences, position-dependent insertion and deletion within loops offer insight into the loop characteristics.

## 2. Materials and Methods

### Dataset Preparation

**Figure 2:**
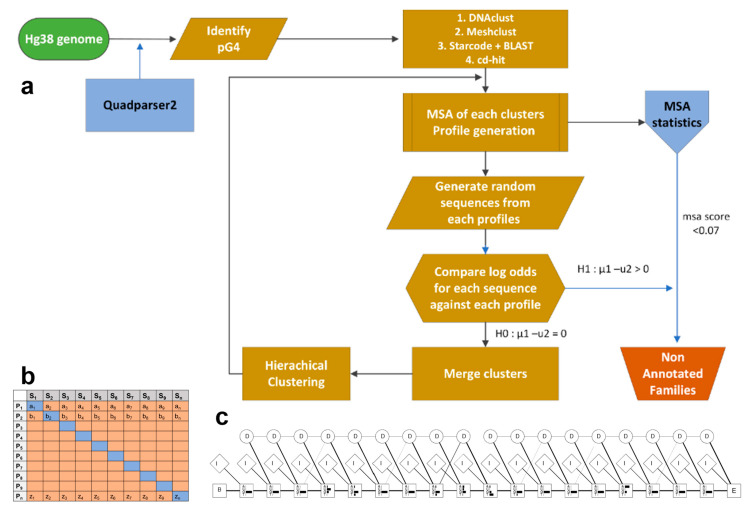
Process for identifying and characterizing G-quadruplex families. (**a**) Workflow diagram for identifying distinct G-quadruplex families. (**b**) Process for identifying appropriate profiles for a specific family. In this case, S_1_, …, and S_n_ represent the list of sequences generated from HMM profile P_1_, …, and P_n_, respectively. We compare the average log odds for input S_1_ over profile P_1_…P_n_ and recursively measure for all the profiles. For each row, the diagonal element is compared with non-diagonal values (log odds) using a Wilcoxon rank sum test with a null and alternate hypothesis, H_0_: T_1_–T_2_ = 0, H_1_: T_1_–T_2_ > 0. (**c**) Profile HMM derived from a selected G4 alignment. Match states are represented as rectangles with four residue emission probabilities indicated as black bars, insert states (I) as diamonds, and delete states as circles. The start and end states are B (begin) and E (end), respectively. Delete states are silent states with no emission probabilities and weighed lines represent the transition probabilities between states.

Since there are no current families or experimental similarities in G4 structures, we start with putative G4s and apply sequence-based methods for clustering. Later, these clusters are used as initial seeds for identifying G-quadruplexes in experimental datasets. Initially, we focused on the G4s identified from Quadparser [25] on the human GRCh38 genome. While two tetrad structures have been experimentally validated, they were omitted from our clustering due to the elevated false positive rate associated with their computational detection. The following process is followed for all groups of sequences based on the number of GGG tetrads (Figure 2a).

1. CD-hit, MeShClust, DNAclust, and a combination of Starcode and BLAST with hierarchical clustering are utilized for the initial clustering of G-quadruplex sequences.
2. Steps (3)–(7) are repeated separately for each clustering method.
3. A multiple sequence alignment (MSA) of each cluster of sequences is carried out in R using the DECIPHER package [66]. The StaggerAlignment and AdjustAlignment functions are used to separate regions of alignment and gaps are shifted to improve the alignment.
4. Clusters with fewer than four sequences are filtered out. An MSA score for each cluster is calculated as the average number of gaps in each column of an alignment divided by the length using MStatX [67].
5. Each alignment is trained as a model profile HMM using HMMER 3.0 [68] and the aphid package [69] in R version 3.4.1 independently. The transition and emission probability matrices are estimated based on the plan7 PHMM model based on Durbin [60]. An example of a profile HMM stating match, insert and delete state is shown in Figure 2b. There are seven outgoing transitions based on the match, insert and delete states, i.e., I_n_ → I_n_, M_n_ → I_n_, M_n_ → M_n+1_, M_n_ → D_n+1_; D_n_ → M_n+1_, D_n_ → D_n+1_; I_n_ → M_n+1_ where n represents each position of the alignment (except the final position). The observed counts of emissions and state transitions are converted into probabilities.
6. The sequences in each cluster are used as input for all the profiles and the log odds scores are generated using the forward algorithm.
7. A pairwise Wilcoxon rank sum test is carried out to compare each profile using the log odds between the profile HMM through which the sequences were generated and all other profiles (Figure 2c). If a profile is diverse (*p*-value < 0.05) against all compared profiles, has a probability of 0.99 for the tested sequences, and has a gap score less than a threshold of 0.10, the profile is saved as a family. For the sequences that are non-significant (*p*-value > 0.05) the sequences are input to the MSA and are merged and/or clustered using agglomerative clustering. Alignments with a gap score of 0.6 after merging are filtered. The process is iterated for a maximum of 100 times.
8. The group of sequences obtained from all the methods is combined and checked for redundancy using a modification of step (7) utilizing a threshold score of log odds 5, Akaike weight of 0.7 and MSA gap score threshold of 0.07 for identifying the final families, which are added manually, checked, and filtered.
9. The alignment and profile HMM are manually verified, resulting in 95 clusters referred to as families. Experimentally validated G-quadruplexes were obtained from processed peaks mapped to hg19 from GEO, accession GSE63874 [41] using bedtools [70] and quadparser2 after conversion to human genome hg38 coordinates by liftover. The models are used as a trained classifier to identify additional sequences. G4 sequences from experimental G4 seq were tested against the cluster HMMs. The likelihood that a query sequence fits the model of an individual family is calculated using the forward algorithm [71], and the normalized Akaike weights [72,73] are calculated. The maximum Akaike weight of the query given to a particular model is selected as the nearest family to the query sequence. The families are manually verified and the variability of sequences in the families is further analyzed based on the annotation of the G4, thermodynamic scores (folding energy), G4Hunter scores, and literature. The steps below highlight the method for the combination of Starcode and BLAST with hierarchical clustering.
  - Levenshtein distance is used to identify the nearest group of sequences which are then filtered based on the length of the sequence and the number of G tetrads. Starcode [64] utilizes a modified Needleman-Wunsch dynamic programming approach known as the poucet algorithm for determining the initial and nearest groups of sequences. Sequences below a fixed Levenshtein score are used to identify the groups, and each group is filtered by the length of the sequence and loop sequence content. Using specific Levenshtein distance as a constraint through this algorithm, one or two nucleotide mismatches can be identified in short DNA sequences.
  - The remaining sequences from step (1) that are not in any group are passed through BLAST for pairwise all vs. all BLAST. −log(E value) is used as the similarity metric.
  - Hierarchical clustering is applied by comparing the agglomerative, Ward, complete, and divisive methods of clustering. The number of clusters is calculated based on the sum of the within-cluster inertia. The optimal number of clusters is the maximum difference from two successive clusters between the groups, i.e., max (I_m_/I_m+1_). The mode of the number of clusters was selected as the optimal cluster.
  - Pairwise alignment of sequences of individual clusters obtained from steps (a) and (c) is carried out using the pairwise alignment function in the Biostrings [74] package. Hierarchical clustering of the sequences is performed based on the pairwise distance. The Consensus of Silhouette [75], Frey index, Macclain Index, Cindex, and Dunn index were used for identifying the optimal number of clusters. The metrics are calculated using the NbClust package [76] in R.

## 3. Results

In the preliminary step, a combination of Starcode and BLAST was used with hierarchical clustering to identify 2717 clusters of G-quadruplexes with 29,112 sequences. Using DNACLUST, 587 clusters with 4664 sequences were identified. A total of 786 clusters with 6335 sequences were identified with Cdhit with a k-mer of 8. MeShClust with an identity threshold of 90% and k-mer size of 9 was able to identify 508 clusters. Any clusters with fewer than four sequences were discarded. The two largest clusters had 1720 and 1410 sequences, respectively. The overall clustering summary is provided in Table 1.

The HMMs for the identified clusters were utilized to predict additional G-quadruplex sequences. In addition, the MSA was used to detect transcription factor site motifs found within each family. The G4 families suffered from the redundancy of motifs because of the high percentage of guanine bases. To identify unique motifs, a pipeline was created to merge and re-cluster the families. Overall, the Starcode and BLAST pipeline identified 95 clusters of G-quadruplex genomic DNA sequences. The MeShClust pipeline identified 72 clusters, while DNACLUST And CD-HIT identified 31 and 30, respectively. The final iteration of the clustering and merging sequences across profiles from the various clustering approaches resulted in 95 distinct families. 

### 3.1. G-quadruplex Families

The resulting 95 families were created from 1739 distinct individual G4s identified from 2145 distinct regions of the hg38 human genome. Given the short sequence length and guanine composition, many of the G4 sequences are not unique. One of the largest families identified, Family 23 is composed of 163 regions with 118 distinct G4s occurring over 122 genes (Supplemental Table S1). Similarly, Family 79 has 130 regions with 99 distinct G4s occurring over 128 genes distributed across all chromosomes (Supplemental Table S2). We identified multiple sequence repeats capable of forming multiple G4 structures with different conformation in Families 46, 62, 88, 89, and 90 based on the available guanines (Supplemental Table S3). Smaller Families 2 and 3 have 7 and 6 distinct sequences occurring in proximity to 8 and 7 genes, respectively (Supplemental Tables S4 and S5). A summary of the predicted G4 sequence families is presented in Table 2.

We analyzed the clusters for their sequence characteristics, functional annotation, and structural features, as presented below. We highlight some of the clusters that have strong biological significance with related biological and molecular processes, including Family 4 (Supplemental Table S6), Family 32 (Supplemental Table S7), Family 75 (Supplemental Table S8), and Family 80 (Supplemental Table S9). 

### 3.2. Categorical Enrichment of Select Families

**Figure 3:**
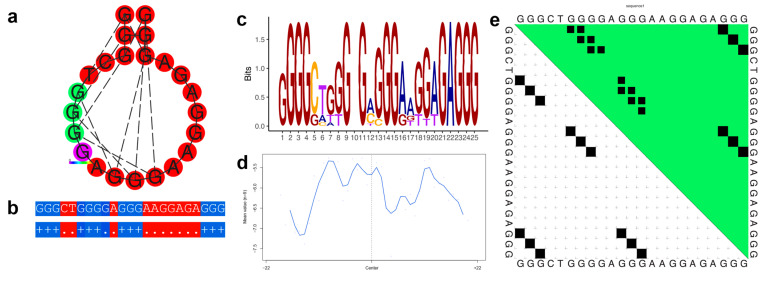
Thermodynamic properties for Family 4. (**a**) Centroid secondary structure with a minimum free energy of −9.64 kcal/mol using the consensus sequence of the family. (**b**) Dot-bracket notation showing the secondary structure. (**c**) Sequence logo representing the per base information content. (**d**) Electrostatic potential generated from all the sequences of the family using 10 flanking bases on either side of the identified G4. (**e**) Dot plot showing the substructures with the highest probabilities.

Family 4 consists of nine sequences distributed over nine genes and seven chromosomes. Figure 3 illustrates the dot-bracket notation of the consensus of the family, along with thermodynamic characteristics. While this family is relatively small, the associated genes are related, showing an enrichment of terms related to neural cells (e.g., glia-guided migration, synapse assembly, dendritic spine development, and gliogenesis) (Supplemental Figure S1, Supplemental Table S10).

**Figure 4:**
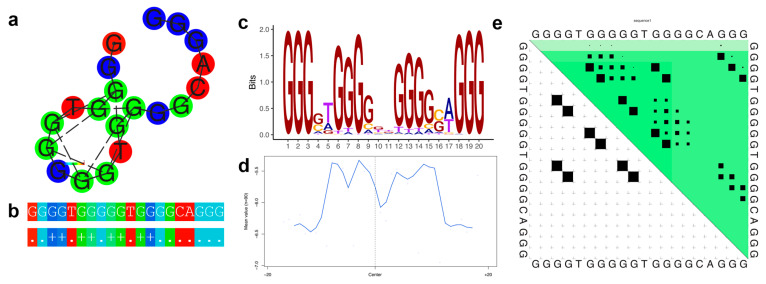
Thermodynamic properties for Family 32. (**a**) Centroid secondary structure with a minimum free energy of −18.0 kcal/mol using the consensus sequence of the family. (**b**) Dot-bracket notation showing the secondary structure. (**c**) Sequence logo representing the per base information content. (**d**) Electrostatic potential generated from all the sequences of the family using 10 flanking bases on either side of the identified G4. (**e**) Dot plot showing the substructures with the highest probabilities.

Family 32 contains 90 G4 sequences annotated with 85 genes. The thermodynamic properties are illustrated in Figure 4. The genes associated with Family 32 G4s are enriched for cellular organization (e.g., positive regulation of cell projection organization and positive regulation of cellular component organization), axonal development (e.g., neuron projection guidance, axon guidance), mitochondrial localization (e.g., regulation of protein targeting to mitochondrion and regulation of establishment of protein localization to mitochondrion) and size regulation (e.g., regulation of anatomical structure size and regulation of cell size) (Supplemental Figure S2, Supplemental Table S11).

**Figure 5:**
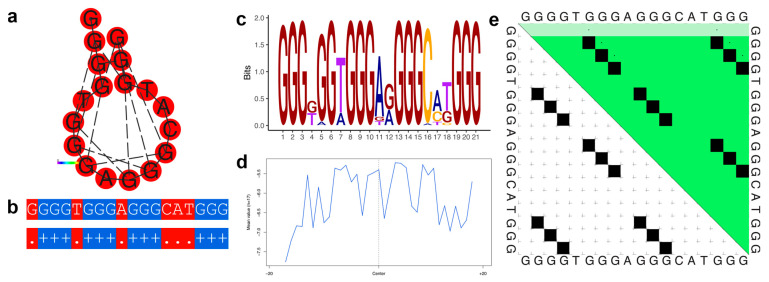
Thermodynamic properties for Family 75. (**a**) Centroid secondary structure with a minimum free energy of −22.82 kcal/mol using the consensus sequence of the family. (**b**) Dot-bracket notation showing the secondary structure. (**c**) Sequence logo representing the per base information content. (**d**) Electrostatic potential generated from all the sequences of the family using 10 flanking bases on either side of the identified G4. (**e**) Dot plot showing the substructures with the highest probabilities.

Family 75 is represented by 18 G4 sequences distributed over 10 chromosomes and 16 genes (Figure 5). Enriched GO:BP terms are highly related to immune differentiation and adhesion (e.g., positive regulation of T cell differentiation, positive regulation of lymphocyte differentiation, positive regulation of leukocyte cell-cell adhesion) (Supplemental Figure S3, Supplemental Table S12).

**Figure 6:**
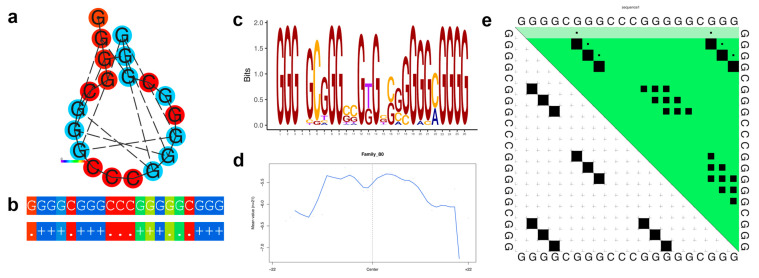
Thermodynamic properties for Family 80. (**a**) Centroid secondary structure with a minimum free energy of −17.38 kcal/mol using the consensus sequence of the family. (**b**) Dot-bracket notation showing the secondary structure. (**c**) Sequence logo representing the per base information content. (**d**) Electrostatic potential generated from all the sequences of the family using 10 flanking bases on either side of the identified G4. (**e**) Dot plot showing the substructures with the highest probabilities.

**Figure 7:**
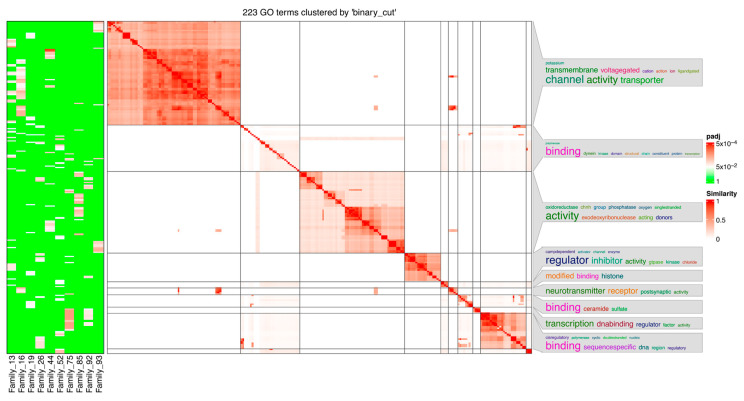
Summary of enriched GO terms for select families as determined by the GOProfiler and simplifyEnrichment R packages.

For Family 80, we identified 21 sequences distributed over 12 chromosomes and 21 genes (Figure 6). Genes associated with this family appear to be localized to cellular components, in particular membranes. Enriched GO:CC categories for the genes include the cytoplasmic side of the membrane, plasma membrane, cytoplasmic side of the plasma membrane, plasma membrane region, cell projection membrane, ficolin-1-rich granule membrane, side of the membrane, cell periphery, ruffle membrane, secretory granule membrane, leading-edge membrane, actin filament, the extrinsic component of the cytoplasmic side of the plasma membrane, ruffle, membrane, extrinsic component of the plasma membrane, intrinsic components of the membrane, intrinsic components of the endoplasmic reticulum membrane, plasma membrane protein complex, ficolin-1-rich granule, and tertiary granule (Supplemental Figure S4, Supplemental Table S13). A summary of enriched GO terms as determined from GOprofiler and simplifyEnrichment for selected families is presented in Figure 7.

### 3.3. Thermodynamic Properties of Select Families

The free energy of the thermodynamic ensemble for the consensus sequence of Family 1 was calculated to be −28.11 kcal/mol. The frequency of the minimum free energy (MFE) structure was 50.62% with an ensemble diversity of 0, suggesting a strict conformation of tetrads for the formation of a G4 structure. The minimum free energy for the family was calculated to be −27.69 kcal/mol. This family consists of six training sequences that have a single-length loop with T-T-A loops (represented by 1-1-1 loops). 

For Family 11, the free energy of the thermodynamic ensemble was calculated to be −20.22 kcal/mol. The frequency of the MFE structure in the ensemble is 25.23% and the ensemble diversity is 0, suggesting once again a strict conformation of tetrads for G4 formation. Family 63 is identified with the sequence G_3_AG_3_AG_3_AG_3_ and is found across 24 chromosomes and 97 genes distributed among intronic, intergenic, and promoter regions. The free energy of the thermodynamic ensemble for Family 63 was calculated to be −36.00 kcal/mol, while the frequency of the MFE structure in the ensemble is 100% and the ensemble diversity is 0.00. 

Figure 3a–e, Figure 4a–e, Figure 5a–e and Figure 6a–e illustrate the thermodynamic properties of families 4, 32, 75, and 80, respectively. Figure 3a, Figure 4a, Figure 5a and Figure 6a represent the base pairing of each base in the G-quadruplex sequence. Figure 3b, Figure 4b, Figure 5b and Figure 6b highlight the centroid secondary structure in dot-bracket notation. A base pairing probability matrix is used to identify added information about the ensemble G4 secondary structure. Applied initially to identify different secondary structures of RNA sequences, dynamic programming provides efficient computation of base pairing probabilities for secondary structure formation. The MFE secondary structure highlighting encoding positional entropy (Figure 3c, Figure 4c, Figure 5c and Figure 6c) is calculated using the consensus sequence of the G4 cluster as predicted by RNAfold. DNA shape features such as the minor groove width and electrostatic potential (Figure 3d, Figure 4d, Figure 5d and Figure 6d) depend upon the charge distribution of nucleotides in a DNA sequence and affect the folding into secondary structure and transcription factor binding in these locations [77]. The difference in stacking energies causing the varying hydrogen bonding patterns can be predicted in each dinucleotide step and can be used to infer minor groove width [78]. The guanine amino group repeats in G-quadruplexes affect charge distributions in the minor and major grooves of helical DNA leading to rotation of the tetrads. We use it to annotate the different families of G-quadruplex identified here. A dot plot of the structure with MFE is shown in Figure 3e, Figure 4e, Figure 5e and Figure 6e for each of the selected families. 

When DNA is bent around secondary structures such as helical or G-quadruplex structures, the bend is separated based on dinucleotide sequences. Propeller twist is defined as the twist along the axis making two bases “non-coplanar” [79]. Previous studies have provided evidence for the flexible nature of the GG and GC dinucleotides with low propeller twist while AA shows the highest. The flexible nature of such a structure favors G-quadruplex sequences. Low propeller twist is related to the ability of the nucleotides to slide on each other and stack in a stable manner. For each cluster, we calculated dinucleotide frequency normalized by the individual length of the G-quadruplex, minimum free energy, minor groove width, propeller twist, helical twist, roll, and electrostatic potential with −10 and +10 region around the identified clusters of G-quadruplex using DNAshapeR [80]. These features address the shape, thermodynamic stability, and flexibility of rotation of the guanine amino groups, and transcription factor recognition site.

### 3.4. Classification of Experimentally Validated G4 Sequences 

Using the sequences from peaks mapped from a G4 seq experiment (GEO accession GSE63874), and identified using Quadparser2, we found all possible pG4 sequences with four tetrads and used it to query the model classifier. We classified 18,340 individual G4s identified from 22,226 distinct regions of the hg38 human genome into 95 families. Based on the clustering for experimental sequences, the major families represented are Family 73 (917 unique G4s related to 664 genes), Family 2 (25 unique G4s, 29 genes), and Family 93 (26 unique G4s, 25 genes). Family 63 has a distinct G4 sequence G_3_AG_3_AG_3_ that is repeated throughout the genome, occurring 313 times over 23 chromosomes and 204 genes.

### 3.5. G4 Repeat and Loop Length Characteristics

**Figure 8:**
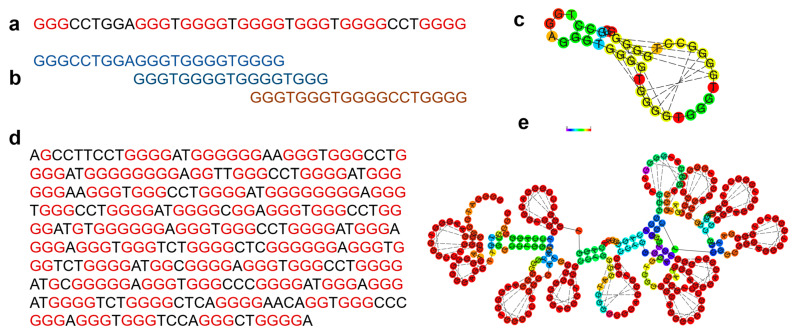
Example sequences with multiple tetrads. (**a**) G-quadruplex sequence from chr19:43479561-43479598 overlapping the *PHLDB3* gene with guanines labeled in red. (**b**) Three possible alternate G4 regions for the *PHLDB3* region. (**c**) MFE structure for the *PHLDB3* region. (**d**) G-quadruplex sequence from chr17:81432609-81432932 overlapping the *BAHCC1* gene with guanines labeled in red. (**e**) MFE structure for the *BAHCC1* gene.

For genes with repeats of G4 sequences (i.e., more than four tetrads), multiple G4 sequences with a variable loop length are possible (Figure 8). We identify all possible linear combinations of G tetrads for such sequences and classify all combinations of the sequences into families. This provides a way to identify multiple conformations forming G-quadruplexes. One example gene with a variable length sequence is *BAHCC1*, a chromatin regulator known to interact with transcriptional repressors to ensure gene silencing through recognition and bind to PRC2 complex mediated H3K27me3 through chromatin compaction and histone deacetylation [81,82]. Within a single G4 region, we identified repeats of 13 different sequences (length of G4 repeat: 314 bases), with each sequence being distinct enough to occur in a separate family. We also find 29 G4 sequences in *NRD2*, with most of the sequences occurring in Family 17, with one each also occurring in Family 7 and 10. 

We identify similar repeats of five distinct sequences spanning an intronic region in *PLOD1*, which codes for lysyl hydroxylase and is involved in collagen synthesis. A 45 nucleotide G-quadruplex sequence present in the promoter region of tyrosine hydroxylase (*TH*) can regulate transcription and has been linked with neurological and psychological disorders such as Parkinson’s and schizophrenia [83,84]. We found two additional G-quadruplex sequences in the opposite strand across promoter and intronic regions of *TH* which have matches to Family 14 and 37, respectively.

Semaphorins are a group of membrane-spanning proteins that bind to Plexin (PLXNA and PLXNB) receptors to regulate axon cue signaling, cytoskeletal development, and cell adhesion [85,86]. The regulation and signaling of SEMA proteins within the plexin family have been a topic of study, and we identified 39 and 37 distinct G-quadruplex forming sequences in the SEMA family and PLXN family, respectively, with similar G4 loops present in both genes. The prediction identified multiple G4 sequences present in *SEMA6C*, *SEMA6D*, and *PLXND1* with the highest match to Family 48 (Table 3). Similarly, *SEMA4D*, *SEMA4B*, and *PLXNA4* shared sequences occurring in Family 17. These findings suggest that multiple regions can form G-quadruplex in these genes, resulting in multiple conformations that might allow for differentiation for methylation in a pattern-specific manner.

The PDB structures 22AG, 2KF8, 5LQG, and 5YEY represent telomeric quadruplex DNA forming a range of conformations with antiparallel topology based on varying physiological conditions. These telomeric G4 sequences are determined to have the highest likelihood of matching Family 22. They have a similar loop size to structure 2KM3 [28], which has a variant of CTAGGG repeat instead of TTAGGG repeats. The 2KM3 structure forms a chair-type G-quadruplex in the K^+^ solution and is most similar to Family 33. Based on the sequence characteristics, these differences in structure which are caused by a one or two bp change can affect the overall prediction of the glycosidic conformation. This, in turn, can be used to help understand the structure based on the local environment and interacting conditions.

The 2LXQ G4 structure is found upstream of the pilin expression locus in *Neisseria gonorrhoeae*, a human pathogen 5′-G_3_TG_3_TTG_3_TG_3_ sequence is implicated in pilin antigenic variation [87]. Known to form an all-parallel stranded topology, the sequence was predicted to have the highest likelihood score with Family 40. A highly conserved G4 sequence at NHE III_1_ upstream of promoter one has been studied and identified to silence transcription of *c-MYC* [52,88,89,90,91] and other short-loop G4 sequences that form a similar topology. TAG_3_AG_3_TAG_3_AG_3_T was predicted to belong to Family 52 as well as Family 1. Despite following the same 1:2:1 pattern as the 2LXQ structure, the presence of adenosine in place of thymidine as the linker loops is considered a different family. 

Experimental evidence shows that G4s with short-loop sequences favor a parallel topology, while structures with longer loops tend to form hybrid or antiparallel structures [92]. Sequences with thymine compared to adenine as a single-length loop have been found to have a higher melting point than a single A base [93]. Given our clustering scheme, multiple sequences with short loops can show high log odds for multiple families. In these cases, the Akaike weight can help guide the context and identify multiple families containing such sequences.

### 3.6. G4 in Enhancers

Potential regulatory roles of G4 families were analyzed by looking at the overlap between G4s and enhancers. The overlapping enhancers were then used as input into the Gene-Enhancer link correlation (http://compbio.mit.edu/epimap/ (accessed on 3 March 2023)) to determine if any of the overlapping enhancers were correlated with gene expression, and if so, in what cell type. We then performed hierarchical clustering of the intersecting G4s based on the correlations. Two main groups of interest result. 

In the first group, 102 G4 sequences are found in 158 genes, belonging to 57 distinct G4 families. GO:BP analysis of this group results in terms associated with immune system processes (e.g., T cell receptor signaling pathway, regulation of leukocyte proliferation, interleukin-10 production, and regulation of cytokine production involved in immune response) or signaling cascades (e.g., positive regulation of ERK1 and ERK2 cascade, calcium ion transmembrane import into the cytosol, and Fc receptor signaling pathway) (Supplemental Figure S5, Supplemental Table S14).

The second group had a ubiquitous high correlation with all cell types in the dataset (Supplemental Figure S6). We identified 234 genes in this group with 107 distinct G4s belonging to 55 distinct families and found enrichment of terms relating to immune responses (e.g., defense response to the virus, cytokine-mediated signaling pathway, and regulation of defense response), regulated cell death (e.g., apoptotic signaling pathway, extrinsic apoptotic signaling pathway via death domain receptors, and positive regulation of programmed cell death), lipid biosynthesis (e.g., regulation of lipid biosynthetic process and response to a fatty acid), and migration (e.g., positive regulation of protein localization and positive regulation of mononuclear cell migration) (Supplemental Figure S7, Supplemental Table S15).

Based on the enriched terms of the two groups, it appears as though the G-quadruplex functions across multiple pathways in different cell types. It is possible that tissue-specific conditions control the actual G4 formation, leading to tissue-specific functional regulation. The results of the enhancer-gene correlation related to the presence of G4 sequences in enhancer regions in group 1 are more likely to affect genes in the thymus, T cell, and lymphoblastoid cells.

## 4. Discussion

Our clustering methodology presented here has allowed for the construction of families of G-quadruplexes based on sequence similarity, loop length and composition, and thermodynamic properties. Further analysis of these families uncovers that many of these families have functional enrichments, indicating they are potentially regulated by common mechanisms since they have structural similarities. Comparing our results to the only previously studied family, Pu27, shows a high agreement, with 12 of the 18 Pu27 members belonging to Family 1 (Table 4).

Multiple transcription factors can bind to the alternative motifs present in G-quadruplex regions [57] in response to environmental conditions and in response to stimuli. These conditions trigger the folding and unfolding of G4 structures. We identify Family 40 as an alternate conformation in these sequences, as multiple tetrads allow the alternate guanine bonds for a stable structure. Nucleoside diphosphate kinase (NM23-H2) [94,95] has been previously identified to unfold Pu27, causing the increase of c-MYC transcription while nucleolin [96] has been identified to stabilize the G4 structure. The mechanism of TF binding and the control of the expression of the c-MYC gene is poorly understood and is beyond the scope of prediction through this model. However, this process sheds light upon the collection of multiple conformations of structures in equilibrium which can alter the change in binding grooves for transcription factors and a further downstream process. Failing to take the dynamic nature of Pu27 and other G-quadruplex sequences in the genome into account could limit the effectiveness of any therapeutic compounds designed to target it.

Several G4 ligands are currently being considered for their therapeutic value. For instance, CX-5461 is utilized for the treatment of BRCA1/2 deficient tumors through topoisomerase II inhibition [97,98], and melanoma cell lines have been treated with G4 ligand RHPS4 that targets the *MYC* gene [99] among others. G4 ligands such as APTO-253 [100], TMPyP4 [101], and telomestatin [102] have been tested for their effect on leukemia. Despite showing promising results and inhibition of cell growth, telomerase shortening and senescence were observed with some of the G4 ligands in different leukemia cells [103]. With the information on G4 formation and binding of specific ligands to multiple G4 structures, identification of G4 clusters can provide additional information about DNA damage occurring or novel binding motifs of specific G4 ligands. 

G4 structures contribute to genomic instability and the proliferative nature of different cancers. The context and location of individual G4 can serve as a roadblock for many oncogenes, but the presence of G4 in the vicinity of a tumor suppressor gene can have the opposite effect. To understand the intended consequence of these targets for all the G4 ligands, it is important to characterize the thousands of G4 structures present in the genome and classify these structures based on their structure, function, or localization.

This study identifies related families of G-quadruplex sequences within the human genome and presents them as clusters described by both an MSA and HMM. The approach described here can easily be applied to other model organisms where G4s are known to play regulatory roles. Many of these clusters were functionally annotated, allowing for a more complete understanding of these structures as well as the identification of multiple targets for testing of G4 ligands. Currently, our approach utilizes experimentally validated sequences as part of the clustering algorithm which makes it more robust to false negative G4s but also makes it more difficult to compare to strictly computational approaches that might be constructed in the future. However, we do provide all the clustering scripts and resulting family-based HMMs on our Github repository. As more information on experimentally validated G4 regions becomes available, refinement of clustering methodologies will yield more informative G4 families.

## Figures and Tables

**Table 1:** Cluster summary based on different clustering techniques.

Method	Number of Sequences	No of Clusters	No. of Sequences in 2 Largest Clusters	HMM Clusters (Sequences)	HMM Families, 1st Iteration (Sequences)	Final Families Selected (Sequences)
Starcode + BLAST with hierarchical clustering	29,112	2717	419, 323	95 (842)		
DNAclust	9610 (4664)	587	142, 126	31 (1165)		
Cd-hit (kmer 8)	6335	786	182, 115	30 (389)		
Meshclust	14,222	508	1720, 1410	72 (1843)		
Total					220 (3888)	95 (2145)

**Table 2:** Summary of count of G4 sequences identified using predictive models pHMM across different clusters, genes, and chromosomes.

	TRAINING					PREDICTED			
Family	G4s	Chrs	Distinct Sequences	Associated Genes	Consensus Using Training Sequences	G4s	Chrs	Distinct Sequences	Associated Genes
1	15	12	5	14	GGGGTGGGTGGGGAGGG	643	24	118	468
2	8	5	7	8	--GGGARKGGSCTGGGACAGGG	25	13	25	29
3	10	6	6	7	GGGAGGGGGCTGCWGGGATGGGGG	270	22	257	219
4	9	7	8	9	-GGGCTGGG-GMGGGAAGGAGAGGG	106	22	106	95
5	8	6	7	7	GGGKKGGGGWGAATRGGGCAYGGG-	355	23	341	271
6	8	6	6	7	-GGGGKCTCAGGGGCTGGGCAGRGGG	213	23	200	183
7	7	7	7	7	-GGGC-CCSKGGGCDGSGRGGMRGGG	636	24	614	564
8	7	7	7	7	GGG-MCTTGGGGGTKGGGASAA--GGG-	376	23	369	311
9	10	9	8	10	-GGGSTGGGGAGGGTGGG	350	23	136	276
10	20	15	10	20	GGGGTGGGGGTGGGAGGG	261	23	107	187
11	15	12	8	15	GGGRGKKKGGTGGGAGGG	164	23	84	132
12	17	9	17	17	GGGGC-CWGGG-TGGGA-AAGGG-	347	24	330	289
13	64	20	62	62	---GG-RWGGGCYKGG-GGGCWGGG	143	22	125	125
14	52	20	50	51	-GGGRCGGGGCAGGGG-TG-GGG	163	24	153	140
15	13	9	13	13	GGGRRAWRGGGTGGGAGGG	151	22	116	121
16	8	7	8	8	GGGGATKDG-GGGAGGGAGGG	152	23	134	113
17	23	11	16	23	GGGAAGGG---TCAGGG-CCAGGG	312	22	293	286
18	14	11	12	14	GGGTGGGTGGGGKMAGGG	439	23	242	345
19	8	8	8	8	GGGCCMMGGGCTGGGGCAGGG	59	19	59	63
20	8	6	7	8	GGGWDGGSMRGGGCM--CAAGGG	421	23	414	343
21	7	6	7	7	GGGGC-AGGGGCAGGGDGTGAGGGG	130	23	120	101
22	8	6	8	8	-GGGCYAGGGT-TGGGWRAGGG	60	22	49	44
23	163	23	118	122	--GGGTKG--GKGRWG-GGRTGGGGG	794	24	555	603
24	35	19	34	35	GGGGGYRGGGSWGGGGWGGG	107	21	91	84
25	39	18	32	37	GGGRR-GGG-RTGGGG--CCKGGGG	434	23	418	365
26	9	7	9	9	-GGGGBWGGGGKSAGGGWGGG	69	19	67	49
27	11	9	11	11	-GGG-GCTGGGRMCWGGGCWGGG	113	22	107	98
28	79	21	79	79	GGGGA-WGGGMARGGGY-RGGG	87	21	83	67
29	18	15	17	18	GGGSHWGGGGGGKGGGRGGG	108	21	103	98
30	12	6	12	12	GGGGKRKGGGKMWGGGKGGG	209	23	180	174
31	44	18	43	44	GGGGMRGGGGKKGGGGTGGG	107	23	94	88
32	90	23	85	88	GGGSTGGGGKKGGGGSWGGG	164	22	146	130
33	111	22	102	108	GGGCTG-------GGGCKGGG--SCWGGG	210	22	184	160
34	9	6	8	9	GGGAATGGGGGGTGGGGG-GGGG	101	22	98	70
35	25	16	25	25	-GGGCA---GG-GGAGGGMYAGG-----GG	179	22	173	148
36	52	20	46	48	-GG--GCCTKGGGG---WGGGAGGG-	540	23	497	439
37	7	6	5	7	-GGGSCAGGGCCAGGGCCAGGG	137	22	125	124
38	7	7	7	7	GGGGYGGGGGR-CAGGGCCAGGG	207	23	200	199
39	12	8	11	12	GGGGAGRGTGGG-MAGGGTGGG	145	24	143	111
40	21	13	16	20	GGGYTGGGRA-TGGGTGGG	489	23	289	348
41	11	8	10	11	GGGM-CAGGGYKSSGGSSAGGG	100	22	99	88
42	17	13	17	17	GGGA-GGGAGGGRAACYYSRGG-	534	23	522	415
43	17	11	17	17	GGGGCCYGGGCCTGGGGAGGG	68	22	64	73
44	9	6	9	9	GGGC-YAGA-GGGTGGGYWGGG	151	22	141	125
45	28	12	28	28	-GGGSKK-KGGGCAGGGG--CAGGGG-	207	23	196	151
46	8	7	8	8	-GG-GKTGGGGGMWGGGRGGRGGG	83	21	77	61
47	21	13	17	20	--GGGGTGGGA--GGGATGGYGGGG-	134	21	118	101
48	21	13	21	21	-GG-GRTTGGGGGT-GG-GG-RTGGGG	776	24	724	547
49	29	10	12	12	-GGGGGCAGGGCYGGG-GCTGGG	54	21	44	43
50	32	19	30	32	-GGGAGAGGGT--TKGGKGR--AGGG	271	23	252	221
51	12	7	9	10	-GGGGTGGGCAGGGMAGMYTGGG	141	24	136	118
52	9	8	9	9	GGGCCCCSGGGGCGGGGCGGG	265	24	264	309
53	56	19	54	50	--GGGDGT-G-G-GSGG-AGGGAGGG--	155	22	145	127
54	33	18	31	33	GGG-CTCR-GG-RMAGGG-CTGGG	214	24	206	196
55	21	16	21	21	-GGGYR-GGGGTGG-GGGGC---RGGG	111	23	110	112
56	9	7	9	9	-GGGGTGGGGTKGGGG-GKRGAGGG	332	24	319	258
57	14	11	9	14	GGGSC-GGGGCGGGCGGGG	314	23	164	328
58	27	9	15	14	-GGGCTGGGKGRGGGGA-GCAGGG	155	23	132	110
59	44	16	44	44	GGG-SAGGGC-KGGGADRGGGG	265	23	247	226
60	8	7	8	8	-GGGGGTGGGGG--RRWGGGSAGGG	124	21	115	98
61	10	1	9	8	GGGACTYRTGGGCTTTGGGCCAAGGG--	106	21	105	106
62	10	4	8	6	GGGGAGACTGGGGAGGCCGGGGYRGAAGGGG	73	20	64	45
63	97	24	1	97	GGGAGGGAGGGAGGG	313	23	1	204
64	31	9	16	12	-GGGGTGKG-GGGGGGRMSGGGG	54	17	42	29
65	16	11	9	15	GGG-GARTGGGCYGGGATGGG-	97	21	86	72
66	58	21	49	53	-GG-----STGGG--CCYTG--GGK-TG--GGG	268	23	260	236
67	6	4	6	6	GGGGTGGG-CATGGGAG-GCAGGG-	214	23	200	171
68	13	1	12	12	-GGGGAGG-GGGGTGCCCTGGGTTGGG-	138	20	118	119
69	11	7	8	11	GGGCAW-GAGGG-A-G-GGKTGGG	129	22	119	99
70	19	11	14	16	GGGRGKTGGGTGGGGGTGGG	202	23	155	161
71	6	5	5	5	GGGGAAGGGACAGGGGMMRGGG	162	23	157	157
72	10	8	8	10	GGGSWG-CAGGG---AGGGCTGGG-	206	22	188	158
73	12	10	11	12	GGGTG-GGGTGGGGK-KRGATGGG-	947	23	917	664
74	12	8	12	11	GGGTGGGGRCAAGGGTRGGG	142	22	129	119
75	18	10	13	16	-GG-GGTGGGA-GGGCMKGGG	343	23	180	265
76	6	4	6	6	GGGGTGGGTGGGG-RATGAGGGG	451	24	420	329
77	19	13	19	19	-GGRRWGGGGRA--ARGAGGGAGGG	296	23	290	223
78	10	9	10	10	GGGGAMT-TGGGGGKGGGG-GGG	329	24	321	268
79	130	22	99	128	-GGGMGGGG-CGGGGCG--GGG	712	24	400	677
80	21	12	21	21	GGG-GCGGGSC---SSGGGGGMGGG-	406	23	389	418
81	13	10	13	13	GGGGRAGGG-T-GGGCTTTGGGG	347	23	329	270
82	38	20	13	34	GGGCAGGGCAGGG-CAGGG	391	24	211	284
83	10	5	8	10	GGGT-CTGGGT--CTGGGTCWGGG-	116	23	111	102
84	6	4	5	5	-GGGGCCGGGGTGGGARGYGGG	66	21	64	62
85	12	8	12	10	-GGGKY-AGGGCCAGGGTGGGGG--	53	21	50	42
86	8	3	4	5	GGGAGGGTCCWGGGGYTGGG	129	22	116	103
87	9	6	9	7	GGGSBCWGGGWS-AGGGAGGG	73	20	69	67
88	11	7	11	11	-GGGRGRCYTGGGTGGGGGGG-	120	22	107	103
89	11	9	6	11	-GGGGTGGGGGTGGGGGGG	43	20	12	40
90	10	8	3	9	-GGGGTGGGGTGGGGGGG	112	23	13	81
91	9	9	9	9	-GG-GGWGGGAGGGAARACKGGG-	75	21	70	70
92	13	7	11	13	GGGKT-GGGGAGGGGAWTWRGGG	451	23	428	367
93	9	8	7	9	GGGCCTGGGCYTGGGCYDGGG-	26	16	25	25
94	12	10	12	12	GGGAMAGGGGGSAGGGGCRGGG	86	20	86	80
95	8	7	8	8	----GGGGACAGGGRCA-GGGVCAGGG	120	21	88	79

**Table 3:** G4 sequences identified in the genic regions associated with the plexin and semaphorin gene families with high similarity to G4 Families 17, 48, and 79.

Location	Sequence	Log Odds	Akaike Weight	Strand	Gene ID	Gene Symbol	Family
chr15:90204178-90204199	GGGAGGGCACTAGGGCCCTGGG	8.987	0.617	+	10509	*SEMA4B*	17
chr3:126991053-126991092	GGGCAGGGCAGGCAGGGAAGGG	10.584	0.892	+	5361	*PLXNA1*	17
chr9:89440465-89440503	GGGTAGGGCTCAGGGGCCAGGG	14.015	0.996	−	10507	*SEMA4D*	17
chr1:151141755-151141776	GGGATGGGGGTTGGGGGGTGGG	13.6	0.828	−	10500	*SEMA6C*	48
chr15:47662210-47662233	GGGGTGGGGGGTGAGGGGATGGGG	11.857	0.994	+	80031	*SEMA6D*	48
chr3:129567938-129567973	GGGTTGGGGTGGGGGGTGGGG	12.652	0.772	−	23129	*PLXND1*	48
chr3:129588350-129588372	GGGTGTCGGGGGTGGGGGAGGGG	9.599	0.787	−	23129	*PLXND1*	48
chr3:122983446-122983465	GGGCGGGGACGGGGCGGGG	12.301	0.981	−	54437	*SEMA5B*	79
chr3:129606851-129606910	GGGCGGGGCCGGGGCGGGG	14.216	0.916	−	23129	*PLXND1*	79
chr3:50276050-50276067	GGGAGGGTCGAGGGCGGG	6.415	0.677	+	7869	*SEMA3B*	79

**Table 4:** Family prediction for previously identified Pu27 family of G4 sequences.

Overall Sequence	Name	Minimum G4 Sequence	Length	Log Odds	Akaike Weight	Family
TGGGGAGGGTGGGGAGGGTGGGGAAGG	Pu27-c-MYC	GGGGAGGGTGGGGAGGG	17	6.99	0.89	1
		GGGTGGGGAGGGTGGGG	17	5.7	0.59	40
		GGGGAGGGTGGGGAAGG	17	4.95	0.45	1
TGGGAGGTGGGGAGGAGGGTTGGGAAGG	Pu1--PLEKHG5	GGGAGGTGGGGAGGAGGGTTGGG	23	7.42	0.53	48
TGGGAGGTGGGGAGGAGGGTTGGGAAGG		GGGAGGAGGGTTGGGAAGG	19	6.93	0.94	15
TGGGGAGGGTGGGGAGGCCGGG	Pu1-2-MYBPHL	GGGGAGGGTGGGGAGG	16	2.41	0.53	1
TGGGGAGGGTGGGGAGGGTGGG	Pu3---	GGGGAGGGTGGGGAGGG	17	6.99	0.89	1
		GGGTGGGGAGGGTGGG	16	7.33	0.9	9
TGGGGAGGGTGGGGAGGGCGGGG	Pu3-SOX2	GGGGAGGGTGGGGAGGG	17	6.99	0.89	1
		GGGAGGGTGGGGAGGG	16	5.62	0.74	1
TGGGGAGGGTGGGGAGGGTGGTGAGGGT GGGGAGGGGGAAGG	Pu5-GRM6	GGGGAGGGTGGGGAGGG	17	6.99	0.89	1
		GGGAGGGTGGGGAGGG	16	5.62	0.74	1
		GGGGAGGGTGGTGAGGGTGGGG	22	7.53	0.26	76
		GGGTGGTGAGGGTGGGGAGGGGG	23	7.47	0.82	73
TGGGGAGGGTGGGGAGGGTGGGGAGGG	Pu7-SDK1	GGGGAGGGTGGGGAGGG	17	6.99	0.89	1
		GGGTGGGGAGGGTGGGG	17	5.7	0.59	40
GGGTGGGGAGGGTGGGGAAG	Pu9---	GGGTGGGGAGGGTGGGG	17	5.7	0.59	40
GGGGAGGGTGGGGAGGGGATGGAA	Pu9-2BC022036	GGGTGGGGAGGGGATGG	17	5.85	0.37	40
GGGAGGGTGGGGAGGGTGGGGAGGG	Pu10-1--	GGGAGGGTGGGGAGGG	16	5.62	0.74	1
		GGGTGGGGAGGGTGGGG	17	5.7	0.59	40
		GGGGAGGGTGGGGAGGG	17	6.99	0.89	1
GGGTGGGGAGGGTGGGGAAGG	Pu10-2--	GGGTGGGGAGGGTGGGG	17	5.7	0.59	40
		GGGGAGGGTGGGGAAGG	17	4.95	0.45	1
GGGGAGGAAGGGGAGGGTGGGGAGGG	Pu11NAV2	GGGGAGGGTGGGGAGGG	17	6.99	0.89	1
		GGGAGGGTGGGGAGGG	16	5.62	0.74	1
GAGGGTGGGGAGGGTGGATGAGGAAGG	Pu14SPTLC2	GGGTGGGGAGGGTGG	15	3.19	0.63	9
TGGGGAGGGTGGGGAGGGTGG	Pu16--	GGGGAGGGTGGGGAGGG	17	6.99	0.89	1
		GGGAGGGTGGGGAGGG	16	5.62	0.74	1
GAGGGTGGGGAGGGTGGGGA	Pu17--	GGGTGGGGAGGGTGGGG	17	5.7	0.59	40
GGGGAGGGTGGGGAGGGAGCTGGGGA	Pu20-CDH4	GGGGAGGGTGGGGAGGG	17	6.99	0.89	1
		GGGTGGGGAGGGAGCTGGGG	20	4.01	0.49	51
TGGGGAGGGTGGGGAGAGGCGGGGTGGGGAGGG	PuX-TM4SF2	GGGAGGGTGGGGAGAGG	17	3.41	0.83	18

## Data Availability

All code and resulting data are available in the GitHub repository (https://github.com/UofLBioinformatics/G4-Cluster (accessed on 3 March 2023)).

## Supplementary Materials

The following supporting information can be downloaded at https://www.mdpi.com/article/10.3390/genes14030645/s1, Figure S1: Top 25 GO:BP enrichments for Family 4; Figure S2: Top 25 GO:BP enrichments for Family 32; Figure S3: Top 25 GO:BP enrichments for Family 74; Figure S4: Top 25 GO:CC enrichments for Family 80; Figure S5: Top 25 GO:BP enrichments for experimentally validated G4s overlapping enhancers, group 1; Figure S6: Correlation of selected enhancers consisting of pG4 with gene expression in multiple cell types utilizing the epimap correlation group-link data; Figure S7: Top 25 GO:BP enrichments for experimentally validated G4s overlapping enhancers, group 2; Table S1: Summary of Family 23; Table S2: Summary of Family 79 G4 sequences; Table S3: Sequence repeats capable of forming multiple G4 structures; Table S4: Summary of Family 2 G4 sequences; Table S5: Summary of Family 3 G4 sequences; Table S6: Summary of Family 4 G4 sequences; Table S7: Summary of Family 32 G4 sequences; Table S8: Summary of Family 75 G4 sequences; Table S9: Summary of Family 80 G4 sequences; Table S10: Enriched GO:BP categories for Family 4; Table S11: Enriched GO:BP categories for Family 32; Table S12: Enriched GO:BP categories for Family 75; Table S13: Enriched GO:CC categories for Family 80; Table S14: Enriched GO:BP categories for experimentally validated G4s overlapping enhancers, group 1; Table S15: Enriched GO:BP categories for experimentally validated G4s overlapping enhancers, group 2.
