# Supplementary material for: Structural and Functional Classification of G-Quadruplex Families within the Human Genome
Source: Genes (Basel). 2023 Mar 4;14(3):645. doi: 10.3390/genes14030645 (PMC10048163; doi:10.3390/genes14030645)

Supplemental Figure S1. Top 25 GO:BP enrichments for Family 4.

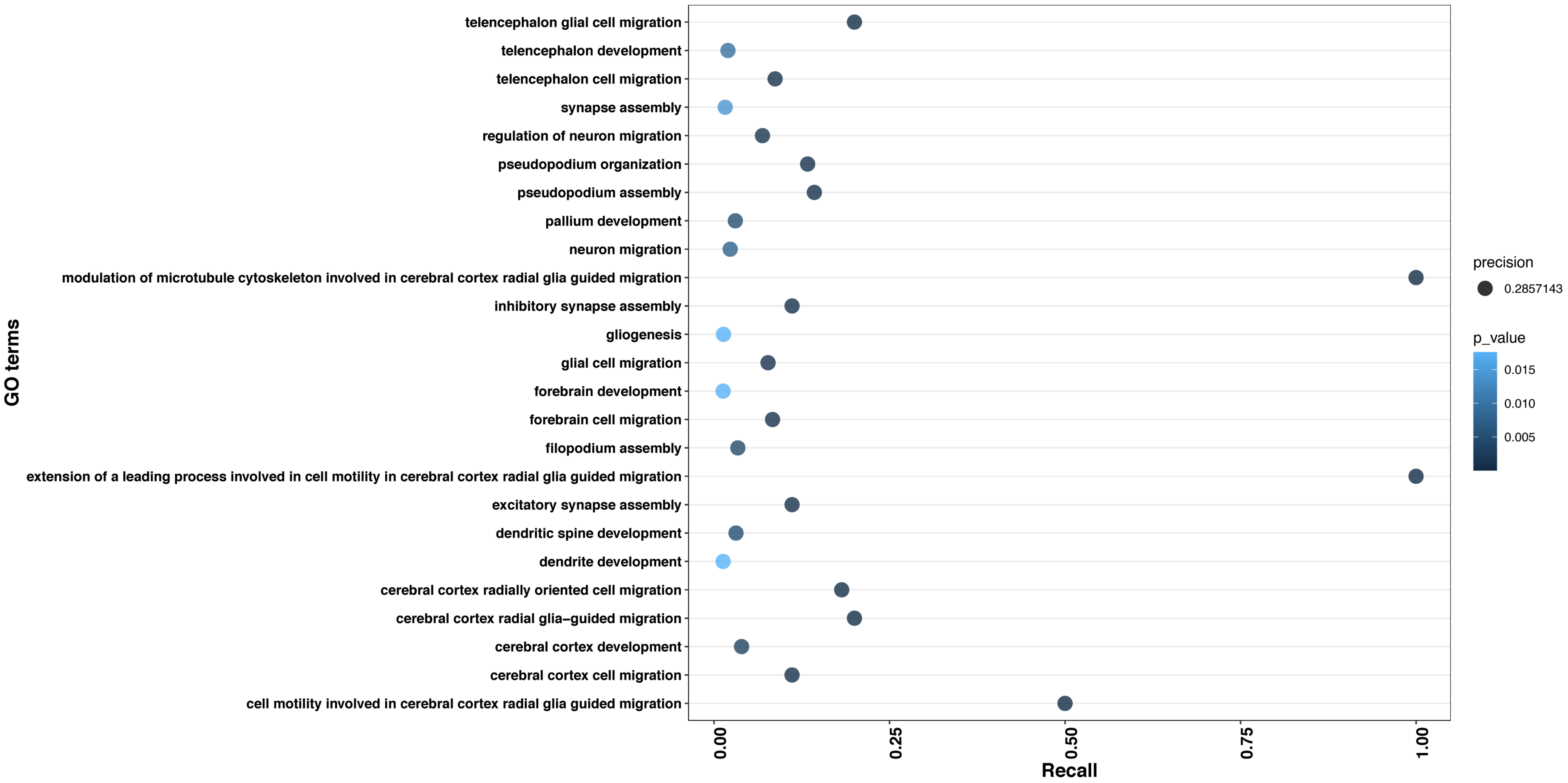

Supplemental Figure S2. Top 25 GO:BP enrichments for Family 32.

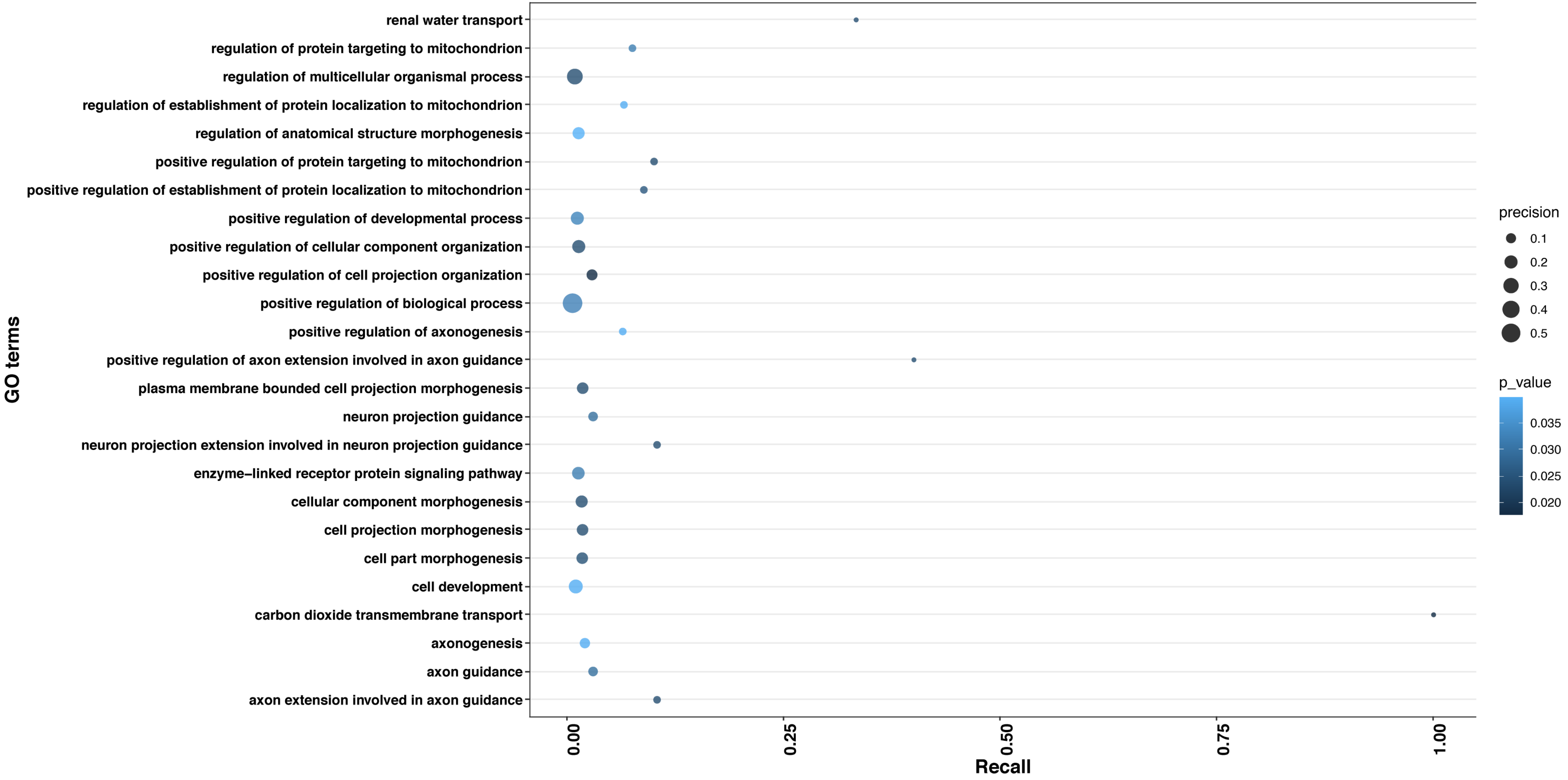

Supplemental Figure S3. Top 25 GO:BP enrichments for Family 74.

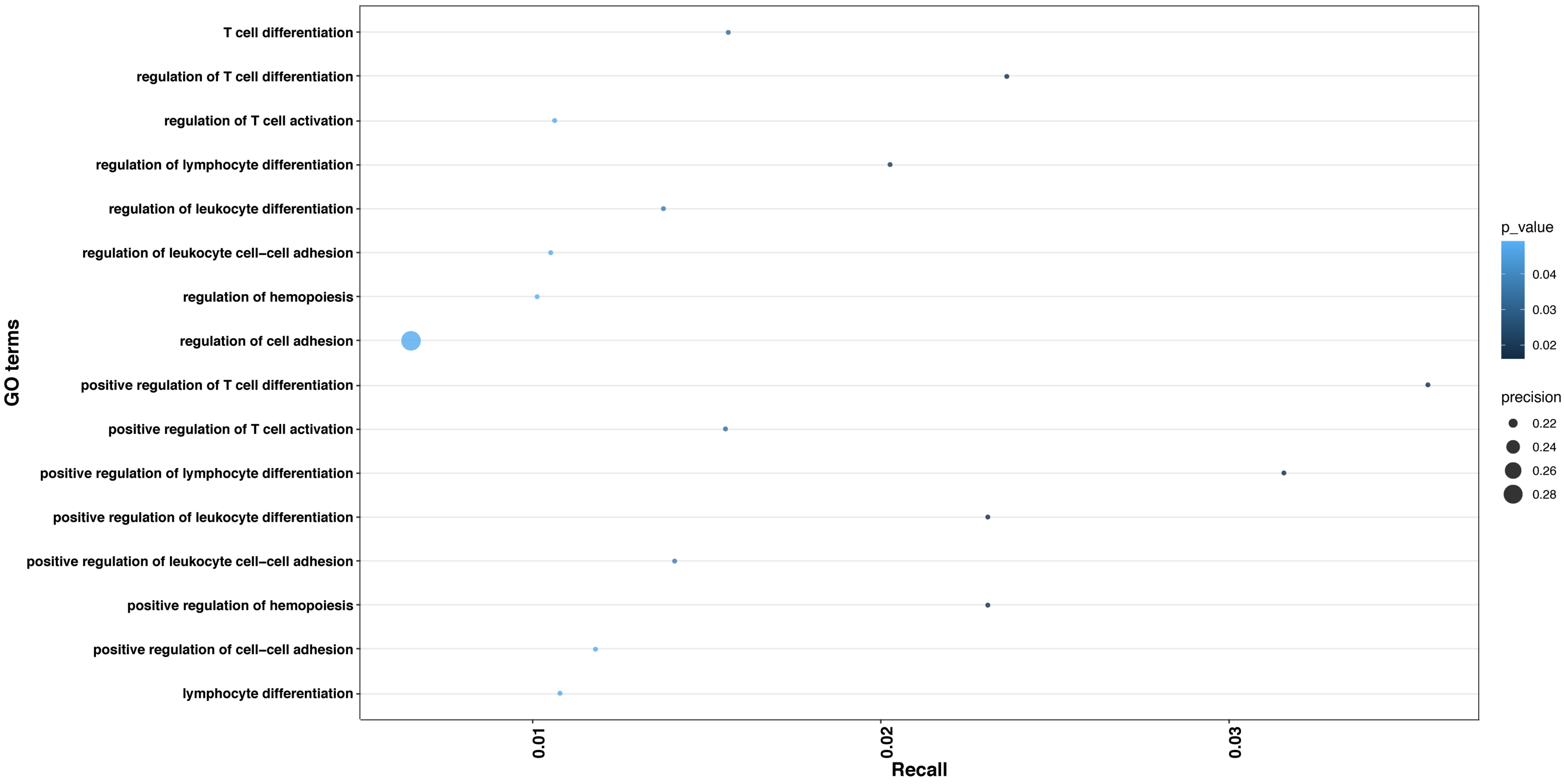

Supplemental Figure S4. Top 25 GO:CC enrichments for Family 80.

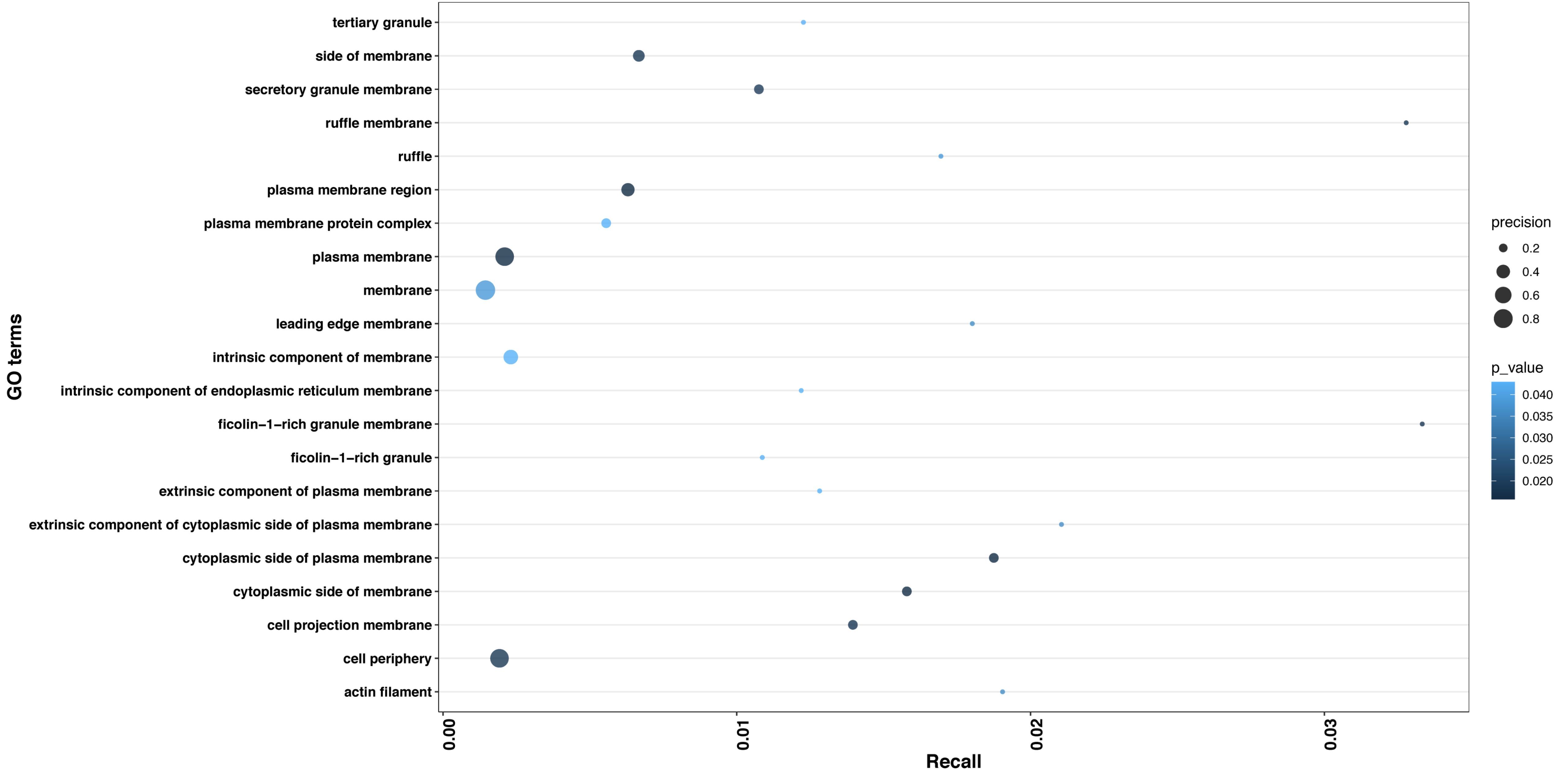

Supplemental Figure S5. Top 25 GO:BP enrichments for experimentally validated G4s overlapping enhancers, group 1.

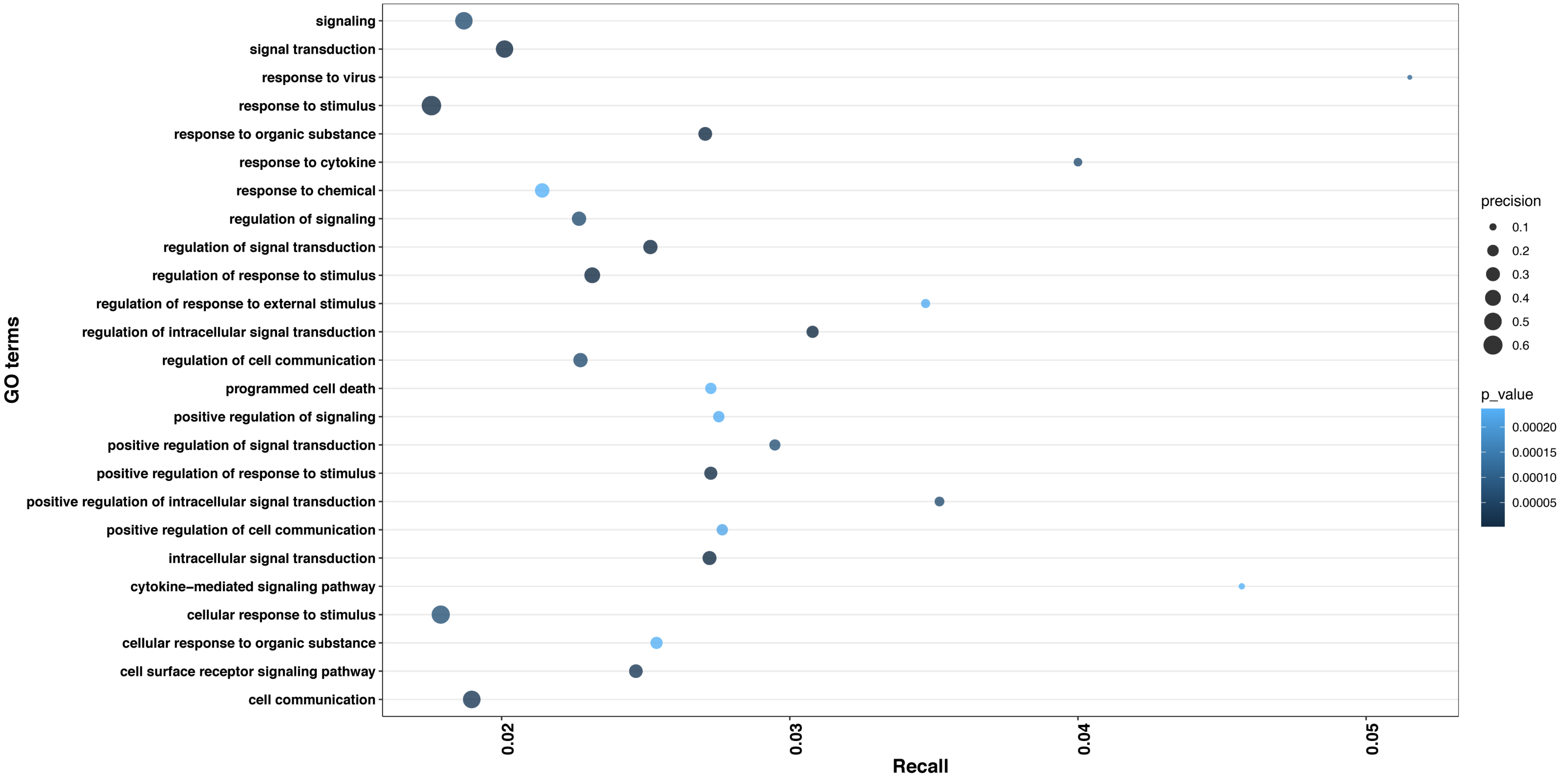

Supplemental Figure S6. Top 25 GO:BP enrichments for experimentally validated G4s overlapping enhancers, group 2.

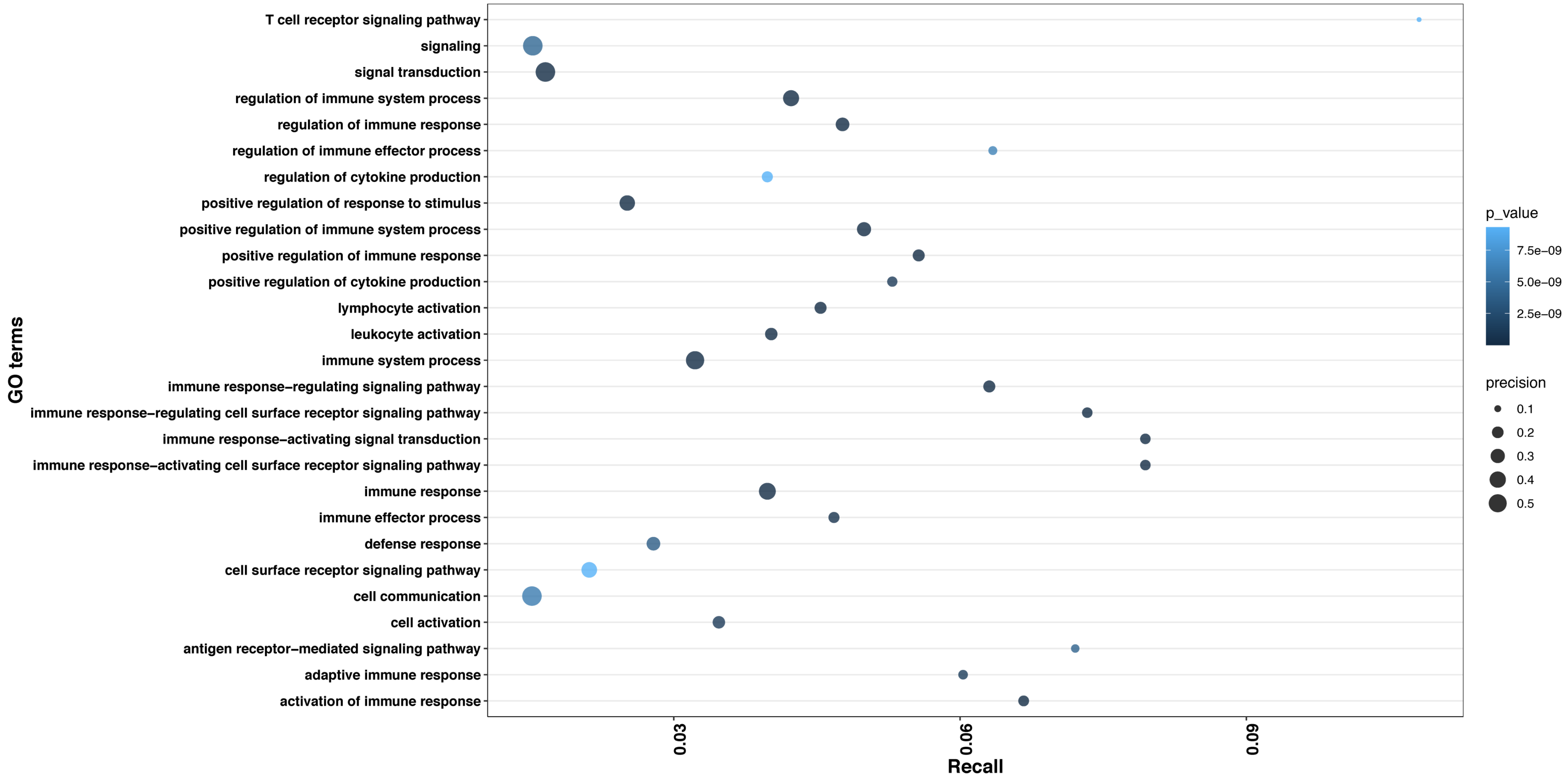

Supplemental Figure S7. Correlation of selected enhancers consisting of pG4 with gene expression in multiple cell types utilizing the epimap correlation group-link data.

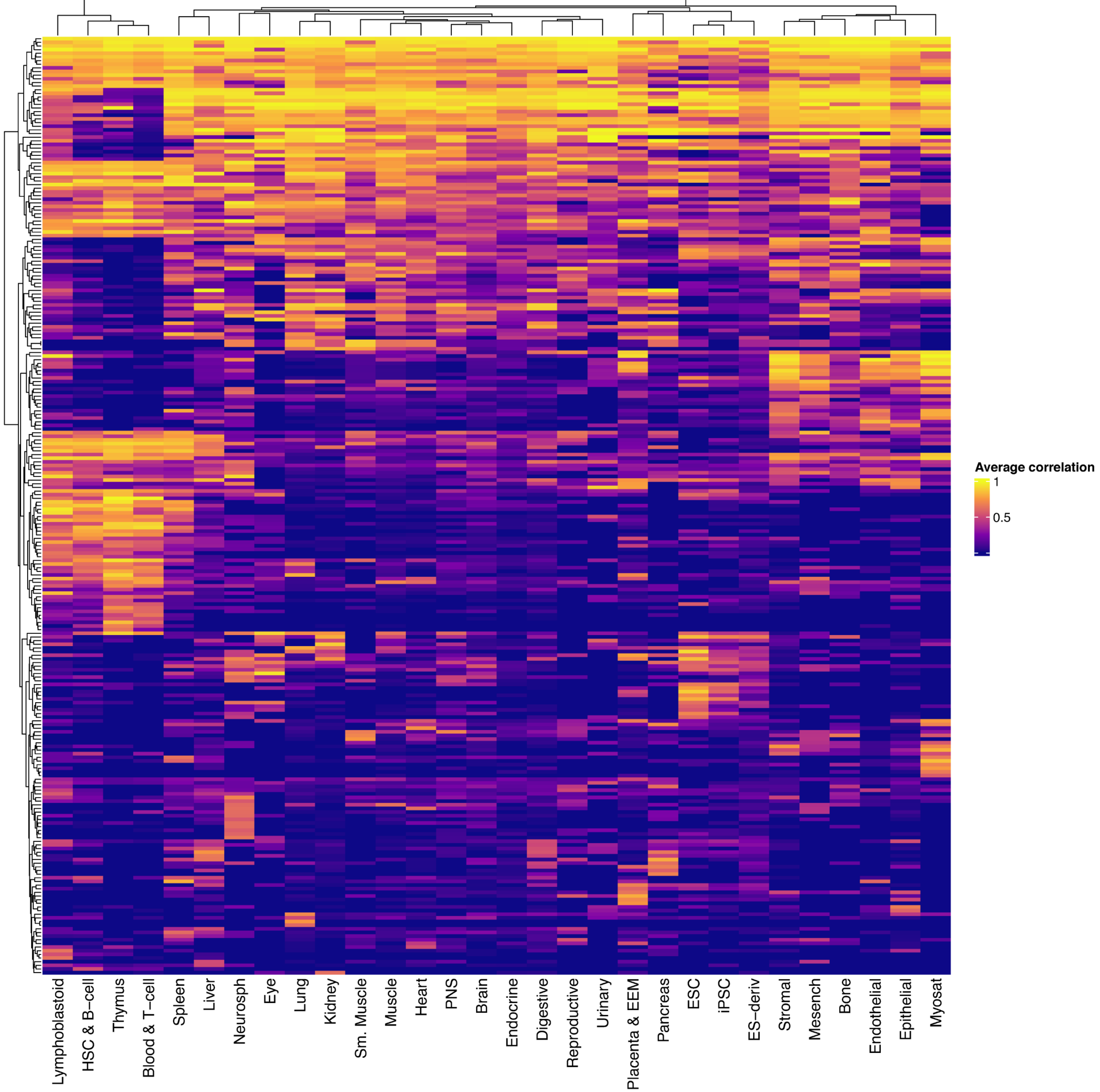

Supplement: Supplementary file 1 [file genes-14-00645-s001.zip › Figures_Supplemental.pdf]
