# Supplementary material for: Structural and Functional Classification of G-Quadruplex Families within the Human Genome
Source: Genes (Basel). 2023 Mar 4;14(3):645. doi: 10.3390/genes14030645 (PMC10048163; doi:10.3390/genes14030645)
Supplement: Supplementary file 1 [file genes-14-00645-s001.zip › Tables_Supplemental.pdf]

Supplemental Table S1. Summary of Family 23.

Sequence	Location	Experimental Evidence	Gene ID	Anntoation
GGGTGGCGGGTGGGGGGAGGG	chr10:123316717-123316737	absent	9184	Intergenic
GGGTGAGGGTGCGGGTGAGGG	chr10:124610344-124610364	present	64077	Promoter
GGGTGAGGGTGCGGGTGAGGG	chr10:124610377-124610397	present	64077	Promoter
GGGTGAGGGTGCGGGTGAGGG	chr10:124610509-124610529	present	64077	Promoter
GGGTGAGGGTGCGGGTGAGGG	chr10:124610542-124610562	present	64077	Promoter
GGGTGAGGGTGCGGGTGAGGG	chr10:124610647-124610667	present	64077	Promoter
GGGTGAGGGTGCGGGTGAGGG	chr10:124610752-124610772	absent	64077	Promoter
GGGTGAGGGTGCGGGTGAGGG	chr10:124610785-124610805	absent	64077	Promoter
GGGTGAGGGTGCGGGTGAGGG	chr10:124610956-124610976	absent	64077	Promoter
GGGTGAGGGTGAGGGTGAGGG	chr10:124610410-124610430	present	64077	Promoter
GGGTGAGGGTGAGGGTGAGGG	chr10:124610857-124610877	absent	64077	Promoter
GGGTGAGGGTGAGGGTGAGGG	chr10:124610890-124610910	absent	64077	Promoter
GGGTGGAGGGGTGGGGTGGGG	chr10:132548209-132548229	present	3632	Intron
GGGTGGGGGGTGGGGAGAGGG	chr10:15725418-15725438	present	8516	Intergenic
GGGTGGGGGGTGGGGAGGGG	chr10:21717394-21717413	present	8028	Intron
GGGTGGGGGGTGGGGAGGGG	chr18:11976921-11976940	absent	3613	Promoter
GGGTTGGGGGTAGGGTGGGGG	chr10:62544042-62544062	present	22891	Intron
GGGGTGTGGGGCAGGGATGGGG	chr10:70678644-70678665	present	140766	Intron
GGGTGGGGGCTGGGGAGAGGG	chr10:78293711-78293731	absent	414243	Intron
GGGTCGGGGGCGGGGGAGGG	chr11:101129211-101129230	present	101054525	Promoter
GGGTGGGAGTGGGATGAGGG	chr11:125159894-125159913	present	103695364	Promoter
GGGTTGGGGAGTGGGGTTGGG	chr11:43926065-43926085	present	100507300	Intron
GGGTTGGGGGTGGGGTGGGG	chr11:44101102-44101121	present	2132	Promoter
GGGATGTGGGAAGGGATGGGG	chr11:69269215-69269235	present	26579	Intergenic
GGGGTGGGTGTGGGGTGGGG	chr12:113876018-113876037	present	9904	Intron
GGGGTGGGTGTGGGGTGGGG	chr16:46909101-46909120	present	84706	Intron
GGGATGGGGGTCGGGTGGGG	chr12:131917175-131917194	present	8408	Promoter
GGGGTGGGGGTGGGAGAGGG	chr12:2537890-2537909	present	775	Intron
GGGGTGAGGGTAGGGATGGGG	chr12:6640013-6640033	absent	84519	Promoter
GGGGTTGGGGAAGGGAGGGGG	chr13:112058816-112058836	present	6656	Intergenic
GGGGTGGGGAAGGGATTGGGG	chr13:53043320-53043340	absent	10562	Intron
GGGTGGGGGTGGGGGGCAGGG	chr14:100443444-100443464	present	79446	Intron
GGGTGGGGGTGGGGCAAGGG	chr14:103532880-103532899	present	115708	Promoter
GGGGTGGGTGAAGGGATGGGGG	chr14:105596969-105596990	present	102465871	Intergenic
GGGGTGGGTGAAGGGATGGGGG	chr14:105597009-105597030	present	102465871	Intergenic
GGGGTGGGTGAAGGGATGGGGG	chr14:105597050-105597071	present	102465871	Intergenic
GGGGTGGGTGAAGGGATGGGGG	chr14:105597091-105597112	present	102465871	Intergenic
GGGGTGGGTGAAGGGATGGGGG	chr14:105597132-105597153	absent	102465871	Intergenic
GGGGTGGGTGAAGGGATGGGGG	chr14:105717880-105717901	present	102465871	Intergenic
GGGGTGGGTGAAGGGATGGGGG	chr14:105717921-105717942	present	102465871	Intergenic
GGGGTGGGTGAAGGGATGGGGG	chr14:105717962-105717983	present	102465871	Intergenic
GGGGTGGGTGAAGGGATGGGGG	chr14:105718002-105718023	present	102465871	Intergenic
GGGGTGGGTGAAGGGATGGGGG	chr14:105718043-105718064	absent	102465871	Intergenic
GGGGTGGGTGAAGGGATGGGGG	chr14:105718084-105718105	present	102465871	Intergenic
GGGGTGGGTGAAGGGATGGGGG	chr14:105718125-105718146	present	102465871	Intergenic
GGGGTGGGTGAAGGGATGGGGG	chr14:105718125-105718146	present	102465871	Intergenic
GGGGTGGGTGAAGGGATGGGGG	chr14:105718166-105718187	present	102465871	Intergenic
GGGGTGGGTGAAGGGATGGGGG	chr14:105718206-105718227	present	102465871	Intergenic
GGGGTGGGTGAAGGGATGGGGG	chr14:105718247-105718268	present	102465871	Intergenic
GGGGTGGGTGAAGGGATGGGGG	chr14:105718288-105718309	absent	102465871	Intergenic
GGGGTGGGTGAAGGGATGGGGG	chr14:105718329-105718350	absent	102465871	Intergenic
GGGGTGGGTGAAGGGATGGGGG	chr14:105718370-105718391	absent	102465871	Intergenic
GGGGTGGGTGAAGGGATGGGGG	chr14:105718411-105718432	absent	102465871	Intergenic
GGGGTGGGTGAAGGGATGGGGG	chr14:105718452-105718473	absent	102465871	Intergenic
GGGGTGGGTGAAGGGATGGGGG	chr14:105718493-105718514	absent	102465871	Intergenic
GGGGTGGGTGAAGGGATGGGGG	chr14:105718534-105718555	absent	102465871	Intergenic
GGGGTGGGTGAAGGGATGGGGG	chr14:105718575-105718596	absent	102465871	Intergenic
GGGGTGGGTGAAGGGATGGGGG	chr14:105718616-105718637	absent	102465871	Intergenic
GGGGTGGGTGAAGGGATGGGGG	chr14:105718657-105718678	absent	102465871	Intergenic
GGGGTGGGTGAAGGGATGGGGG	chr14:105718698-105718719	absent	102465871	Intergenic
GGGGTGGGTGAAGGGATGGGGG	chr14:105718739-105718760	absent	102465871	Intergenic
GGGGTGGGTGAAGGGATGGGGG	chr14:105718780-105718801	present	102465871	Intergenic
GGGGTGGGTGAAGGGATGGGGG	chr14:105718821-105718842	present	102465871	Intergenic
GGGGTGGGTGAAGGGATGGGGG	chr14:105718862-105718883	present	102465871	Intergenic
GGGGTGGGGGTGGGGTGGGGG	chr14:19037660-19037680	present	100508046	Intron
GGGGTGGGGGTGGGGTGGGGG	chr22:15760289-15760309	present	106146148	Intron

Supplemental Table S1. (Continued).

Sequence	Location	Experimental Evidence	Gene ID	Anntoation
GGGTCAGGGGTGGGGTGGGG	chr14:50864482-50864501	present	145447	Intron
GGGTTGGGGGCGGGGGTGGG	chr14:65413539-65413558	present	2530	Promoter
GGGGTGGGGGTGGGGCGGGG	chr14:68908184-68908203	present	87	Intron
GGGGTGAGGGGAAGGGATGGG	chr14:72421211-72421231	absent	9628	Promoter
GGGTTGGGGGGGTGGGTGGGG	chr15:65356691-65356711	present	9543	Promoter
GGGTGGGGCTGGGGTGTGGG	chr15:88391894-88391913	absent	26589	Intergenic
GGGTTTGGGGTGGGGGTAGGG	chr15:89109459-89109479	absent	11057	Intron
GGGATTGGGGGTGGGGGAGGG	chr16:27805003-27805023	present	23247	Intron
GGGGTGGGGGCCGGGATGGGG	chr16:51096031-51096051	present	6299	Intergenic
GGGTGGGGGTGGGCTGAGGG	chr16:67172198-67172217	present	8996	Promoter
GGGAGGGGGGTGGGGGGAGGG	chr16:7709746-7709765	present	54715	3' UTR
GGGGTGGGGGCAGGGTGGGG	chr16:79383220-79383239	present	51741	Intergenic
GGGTGGGGGTGGGAGTCAGGG	chr16:86705437-86705457	present	101928614	Intergenic
GGGAGTGGGGGTGGGGTGGGG	chr17:12982890-12982910	present	60528	Exon
GGGAGTGGGGGTGGGGTGGGG	chr9:76754262-76754282	present	50652	Intron
GGGTTAGGGGTGGGGTGGGGG	chr17:18127574-18127594	present	51168	Intron
GGGGTTGGGGGAGGGAGGGG	chr17:1880069-1880088	present	6117	Promoter
GGGCTGGGGGTGGGGAAGGG	chr17:50867047-50867066	present	400604	Promoter
GGGGTGGGGAGTGGGGTGGGG	chr17:65457424-65457444	present	105827617	Promoter
GGGGTGGGGGGAAGGGAGGGGG	chr17:82020660-82020681	present	201254	Promoter
GGGTTGGGGGAGGGATTGGG	chr18:24852934-24852953	absent	105372028	Intron
GGGGGTGGGGGTGGGGTGGGG	chr18:26503844-26503864	present	284252	Intron
GGGGTTGGGGGTGGGGGGGG	chr18:62610383-62610402	present	54877	Intergenic
GGGGTGGCGGGTGGGGTGGGG	chr18:77413747-77413767	present	2587	Intergenic
GGGTTGCGGGGTAGGGGAGGG	chr19:35851196-35851216	absent	4868	Promoter
GGGGTGGGGGAAGGGAGGGG	chr19:408850-408869	present	126567	Promoter
GGGTGGGGGTGGAGGGAGGG	chr19:5117318-5117337	present	23030	Promoter
GGGTTGGGGGTGGGCTGGGG	chr1:10670083-10670102	present	54897	Intron
GGGTGGGGCTGGGAGTGAGGG	chr1:1075343-1075363	present	401934	Promoter
GGGTTGGGACTGGGGTGGGG	chr1:157198319-157198338	present	2117	Intergenic
GGGGTGGGGATGGGATGGGG	chr1:202304270-202304289	present	59352	Intron
GGGGTGGGGGAAGGGGGTGGGG	chr1:211260741-211260762	present	55758	Promoter
GGGTGGAGGGTGGGGTGGGG	chr1:21865234-21865253	present	3339	Intron
GGGTGGAGGGTGGGGTGGGG	chr1:30271549-30271568	absent	101929406	Intergenic
GGGTGGGGATGGGAGTGAGGG	chr1:23911503-23911523	present	1269	Promoter
GGGGTGGGCGGTGGGGTGGGG	chr1:24584671-24584691	present	400746	Promoter
GGGTTGGGGGTGGGGTGGGGGG	chr1:36566733-36566754	present	1441	Intergenic
GGGTGGGGGTGGGGTGAGGG	chr1:36576371-36576390	present	1441	Intergenic
GGGCAGGGGTGGGGTGAGGG	chr1:44506457-44506476	present	100847089	Intron
GGGGTGGGCGAAGGGAGTGGGG	chr1:53947258-53947279	present	115353	Promoter
GGGGGGGAGTGGGGTGAGGG	chr1:87522994-87523013	absent	100505768	Intergenic
GGGGCTGGGGGTGGGGGAGGG	chr20:2820918-2820938	present	100288797	Promoter
GGGTTGCGGGGTGGGGTGGGGG	chr20:29750401-29750422	absent	245929	Intergenic
GGGTTGCGGGGTGGGGTGGGGG	chr20:30497208-30497229	absent	245929	Intergenic
GGGTTGGGGGAGGGGGTGGG	chr20:32980815-32980834	present	140732	Downstream
GGGGTGGAGGGTGGGGTGAGGG	chr20:34705516-34705537	present	58476	Promoter
GGGTGGGGATGGGGGGAGGG	chr20:43934072-43934091	present	84969	Intron
GGGTTGGGGGGTGGGGTGGG	chr20:46247013-46247032	present	64405	Promoter
GGGTTGGGGGTAGGGGGTGGG	chr21:32537338-32537358	present	59271	Intergenic
GGGTTGCGGGGTGGGGTGGG	chr22:11262057-11262076	absent	81061	Intergenic
GGGACGGGGGTGGGGTGGGG	chr22:18855039-18855058	present	8214	Intergenic
GGGGTGGGGTTGGGGTGGGG	chr22:19999303-19999322	present	421	Intron
GGGGAGGGGGGAGGGATGGGG	chr22:21451131-21451151	absent	23119	3' UTR
GGGTGGGGATGGGGTGAGGG	chr22:48336509-48336528	present	100422916	Intergenic
GGGTTGGGGGTGGGGGAGGG	chr2:10264610-10264629	present	3241	Intergenic
GGGTTGGGGGTGGGATGGGG	chr2:134334522-134334541	absent	4249	Intron
GGGTGGGGATGAGGGTGAGGG	chr2:136030238-136030258	present	101928243	Intergenic
GGGTGGGGATGGGGAGAGGG	chr2:186884280-186884299	present	151112	Intergenic
GGGGATGGGGGAGGGATGGGG	chr2:235026100-235026120	present	23677	Intron
GGGTTGGGGTTGGGGATGGGG	chr2:237474553-237474573	present	79083	Intergenic
GGGTGGGGGGAGGGGGAGGG	chr2:71452928-71452947	present	8291	Promoter
GGGTTGGGAGGTGGGGGAGGGG	chr2:80306685-80306706	absent	1496	Promoter
GGGTGTGGGTGAGGGTGAGGG	chr3:105648950-105648970	absent	214	Intergenic
GGGTGGGGGTGGGGAGAAGGG	chr3:14867703-14867723	absent	152273	Intron
GGGGTAGGGGTAAGGGATGGGG	chr3:170871296-170871317	absent	200916	Promoter
GGGTCCGGGGTGGGGTGGGG	chr3:183908290-183908309	present	100616127	Intergenic

Supplemental Table S1. (Continued).

Sequence	Location	Experimental Evidence	Gene ID	Anntoation
GGGTTGGGGGAGGGATGGGG	chr3:186926653-186926672	present	6480	Promoter
GGGCTGGGGGTTGGGTGGGG	chr3:50367106-50367125	present	11068	Promoter
GGGTATTGGGGTGGGGTGGGG	chr3:73674103-73674123	present	23024	Intergenic
GGGTGGTGGGTGGGGTGGGG	chr3:9937507-9937526	present	78987	Promoter
GGGTGGGGTGGGGTGAGGGG	chr4:13540437-13540456	present	579	Promoter
GGGTTGGGGGTGGGGCAGGG	chr4:1780111-1780130	absent	2261	Intergenic
GGGTTGGGGGTGGGGCAGGG	chr9:99297662-99297681	present	100996569	Intergenic
GGGGTGGGGGTAGGGAGGGG	chr4:185101976-185101995	present	291	Intron
GGGCTGGGGCTGGGGTGGGG	chr4:3820535-3820554	present	152	Intergenic
GGGTCGGGGGTGGGGCGAGGG	chr4:94451863-94451883	absent	10611	Promoter
GGGGAGGGGGAGGGATGGGG	chr5:140141772-140141791	present	101929719	Intergenic
GGGTTGGGGGCAGGGTTGGGG	chr5:64122940-64122960	absent	285671	Intergenic
GGGGTGGGGGAAGGGAAGGG	chr6:35743073-35743092	present	221481	Intron
GGGTTGGGGGTTGGGGGTGGGG	chr6:43679847-43679868	present	55168	Intron
GGGATGGGGGTGGGGGAGGG	chr6:57178779-57178798	present	101927211	Promoter
GGGGTGGGGGAGGGACGGGG	chr7:100472520-100472539	present	81628	Promoter
GGGCAGGGGGTGGGGGAGGG	chr7:128832311-128832330	absent	2318	Promoter
GGGTTGGGGAGAGGGATGGG	chr7:149751402-149751421	absent	84626	Intergenic
GGGTTGGGGGAGTGGGAGGG	chr7:26621451-26621470	present	285941	Intergenic
GGGTTTGGGGAGGGAAGGGG	chr7:4482507-4482526	absent	221937	Intergenic
GGGTGGGGGCGGGGGGAGGG	chr8:101761018-101761037	present	83988	Intron
GGGGTGGGGGCGGGGGAGGG	chr8:113439774-113439793	present	114788	Promoter
GGGTTTGGGGTGGGGTGGGGG	chr8:130519615-130519635	present	50807	Intergenic
GGGTGTGGGGGTTGGGGGAGGG	chr8:142753544-142753565	absent	137797	Promoter
GGGCCGGGGGTGGGGGAGGG	chr8:25689097-25689116	absent	64641	Intergenic
GGGTTGGGGCTTGGGGAGGG	chr8:26653400-26653419	present	1808	Intron
GGGTTGGGGGTGGGGTGGGGG	chr9:114098608-114098628	present	113220	Promoter
GGGTTGTGGGTGGGGATGGGG	chr9:116931301-116931321	present	22954	Intron
GGGATTGGGGATGGGGTGGGG	chr9:134806401-134806421	present	1289	Promoter
GGGGTTGGGGGTGGGGAGGG	chr9:91299523-91299542	present	549	Intron
GGGTGGGCGCGGGGTGAGGG	chr9:98745018-98745037	absent	203286	Intron
GGGTTAGGGGGAGGGGTGGGG	chrX:120755247-120755267	present	643311	Intergenic

Supplemental Table S2. Summary of Family 79 G4 sequences.

Sequence	Location	Experimental Evidence	Gene ID	Annotation
GGGAGGGGAGGGGAGGGG	chr1:11692947-11692964	present	374946	Promoter
GGGAGGGGAGGGGAGGGG	chr1:32936592-32936609	present	127544	3' UTR
GGGAGGGGAGGGGAGGGG	chr10:12887513-12887530	present	83643	Intergenic
GGGAGGGGAGGGGAGGGG	chr11:47595913-47595930	present	114900	Promoter
GGGAGGGGAGGGGAGGGG	chr13:99003017-99003034	present	23348	Intron
GGGAGGGGAGGGGAGGGG	chr16:54930945-54930962	present	10265	Promoter
GGGAGGGGAGGGGAGGGG	chr4:151016295-151016312	present	987	Promoter
GGGCGGGGCGCGGGCGGGG	chr10:133262048-133262066	present	101	Promoter
GGGCGGGGCGCGGGCGGGG	chr10:133262092-133262110	present	101	Promoter
GGGGCGGGGAGGGGCGGGG	chr10:14604436-14604454	absent	83641	Promoter
GGGGCGGGGCGGGGCGGG	chr10:17348902-17348919	absent	338596	Intron
GGGGCGGGGCSSGGGGCGGG	chr11:533399-533416	present	3265	Promoter
GGGGCGGGGCGGGGCGGG	chr14:89701705-89701722	absent	1112	Intron
GGGCGGGAGGGGCGGGG	chr10:3172964-3172980	present	10531	Promoter
GGGGAGGGGCGGGGGGGG	chr11:115164500-115164517	present	23705	Intergenic
GGGGCGGGGGGGGGCGGG	chr11:134401965-134401982	present	27087	Intron
GGGAGGGGCAGGGGCAGGG	chr11:2571906-2571924	absent	3784	Intron
GGGAGGGGCAGGGGCAGGG	chr5:149676067-149676085	present	389337	Intergenic
GGGCGGGGCAGGGGCGGG	chr11:2902105-2902122	present	5002	Promoter
GGGCGGGGAAGGGTGGGG	chr11:2902676-2902693	present	5002	Promoter
GGGGGGGAGGGGCGGGG	chr11:64342922-64342938	present	283234	Promoter
GGGAGGGGCCGGGGCGGGG	chr11:6473947-6473965	present	10612	Promoter
GGGCGGGGCGGGGCGGGG	chr11:65558635-65558652	absent	4054	Promoter
GGGCGGGGCGGGGCGGGG	chr21:45555638-45555655	present	6573	Intergenic
GGGCGGGGCGGGGCGGGG	chr9:124777075-124777092	absent	169611	Promoter
GGGAGGGGCGGGGCGGG	chr11:7020387-7020403	present	7761	Promoter
GGGAGGGGCGGGGCGGG	chr16:371393-371409	present	10573	Promoter
GGGAGGGGCGGGGCGGG	chr19:17747734-17747750	present	23149	Promoter
GGGAGGGGCGGGGCGGG	chr19:44847716-44847732	present	5819	Promoter
GGGAGGGGCGGGGCGGG	chr9:70258881-70258897	absent	100507299	Promoter
GGGGCAGGGGCAGGGCGGG	chr11:79081753-79081771	present	26011	Intron
GGGGAAGGGGGGGCGGGG	chr12:111597150-111597167	present	6311	Promoter
GGGCGGGGCGGGGCGGG	chr12:48350921-48350937	absent	121274	Promoter
GGGGCGGGGCGGGGGGG	chr14:105441449-105441465	present	9112	Promoter
GGGGCGGGGCGGGGGGG	chr5:70126581-70126597	absent	6606	Intergenic
GGGGCGGGGCGGGGGGG	chr5:71126892-71126908	absent	728340	Intergenic
GGGGCGGGGCGGGGGGG	chr7:99770423-99770439	present	1576	Intron
GGGGCGGGGCGGGGGGG	chrX:132751250-132751266	present	100874102	Intron
GGGCGGGGCGGGGGCGGG	chr14:22871353-22871370	present	26020	Promoter
GGGCGGGGCGGGGGCGGG	chr22:37519650-37519667	absent	29775	Promoter
GGGGCGGGGCGGGCGCGGG	chr14:55052093-55052111	present	93487	Promoter
GGGCGGGGCGGGGCGCGGG	chr14:99645483-99645501	absent	84439	Promoter
GGGGCGGGGCGGGGCGGGG	chr1:3718211-3718229	absent	7161	Intron
GGGGCGGGGCGGGGCGGGG	chr15:101724648-101724666	present	123283	Promoter
GGGGCGGGGCGGGGCGGGG	chr17:62808389-62808407	absent	162333	Promoter
GGGGCGGGGCGGGGCGGGG	chr2:19348311-19348329	present	100616307	Promoter
GGGGCGGGGCGGGGCGGGG	chr22:15761338-15761356	absent	106146148	Promoter
GGGGCGGGGCGGGGCGGGG	chr22:42500547-42500565	present	94009	Promoter
GGGCGGGGAGGGGGCGGG	chr15:39366098-39366115	present	400360	Intron
GGGAGGGGACGGGCGGGG	chr15:69298887-69298904	absent	54852	Promoter
GGGTGAGGGGGCGGGCGGG	chr15:88257743-88257761	absent	4916	Promoter
GGGAGGGTGAGGGGAGGGG	chr15:92439783-92439801	present	8128	Intron
GGGCAGGGGAGGGGCGGG	chr16:21511745-21511762	present	100500917	Intergenic
GGGCAGGGGAGGGGCGGG	chr16:87860829-87860846	present	8140	Promoter
GGGTGAGGGGCGGGCGGG	chr16:22008068-22008085	present	730094	Promoter
GGGGAGGGGGCGGGTCGGG	chr16:66604580-66604598	present	123920	Promoter
GGGCGGGGAGGGCGCGGG	chr16:706088-706105	absent	146330	Promoter
GGGCGGGGAGGGCGCGGG	chr16:67530169-67530186	present	79567	Promoter
GGGAGCGGGAGGGGCGGGG	chr16:8847828-8847846	present	5373	3' UTR
GGGCGGGACGGGGCGGGG	chr16:88686619-88686636	present	333929	Promoter
GGGCGGGGTAGGGCGGGG	chr16:9091577-9091594	present	29035	Promoter
GGGGAGGGGGCGGGGGGG	chr17:3635824-3635841	present	23729	Promoter
GGGGAGGGGGCGGGGGGG	chr17:65055061-65055078	present	10672	Promoter
GGGGAGGGGCGCGGGCGGG	chr17:43755320-43755338	absent	50964	Promoter
GGGCGGGGGGGGCGGG	chr17:518280-518295	present	55275	Promoter
GGGCGGGGACGGGGCGGGG	chr17:64130213-64130231	present	2081	Promoter

Supplemental Table S2. (Continued).

Sequence	Location	Experimental Evidence	Gene ID	Annotation
GGGGCGGGGTGGGGCGGG	chr17:74380229-74380246	present	350383	Intergenic
GGGTGAGGGCGGGGCTGGG	chr17:75740069-75740087	absent	3691	Promoter
GGGGAAGGGGGCGGGGGGG	chr17:81067374-81067392	present	10458	Promoter
GGGCCGGGCGGGGCGGG	chr17:81716621-81716637	present	1468	Promoter
GGGGGGGCAGGGGCGGGGG	chr17:9825847-9825865	present	9340	Promoter
GGGCAGGGGCAGGGCGGGG	chr18:37517402-37517420	present	56853	Intron
GGGGCGGGGCGGGCCGGG	chr19:1418556-1418573	present	26528	Promoter
GGGCGGGGAGGGGCGGG	chr19:1940858-1940874	absent	1455	Promoter
GGGCGGGGCAGGGCGGGG	chr19:3557619-3557636	absent	126321	Promoter
GGGAGGGTGAGGGGCGGGG	chr19:45497095-45497113	present	6253	Promoter
GGGCGGGGAGGGGCGGGG	chr1:180632001-180632018	present	9213	Promoter
GGGCGGGGAGGGGCGGGG	chr19:49388163-49388180	present	147872	Promoter
GGGGGGGAAGGGCGGGG	chr19:49556137-49556153	present	51070	Promoter
GGGCGGGGCCGGGGCGGG	chr19:52690611-52690628	present	55769	Promoter
GGGGCGGGCGGGGCGGG	chr19:676544-676560	absent	10272	Promoter
GGGGAGGGGGGCGGGGGGG	chr1:10856271-10856289	present	54897	Intergenic
GGGGAGGGGCGGGGCAGGG	chr1:110161522-110161540	present	388662	Intron
GGGGCGGGGCGGGGTGGG	chr1:156677373-156677390	present	10763	Promoter
GGGGCGGGGCGGGGTGGG	chr3:141402686-141402703	present	253461	Promoter
GGGGGGGCGGGGCGGGG	chr1:209825787-209825803	present	27042	Promoter
GGGCGGGAAGGGCGGGG	chr1:228276035-228276051	present	84033	Promoter
GGGCGGGGGAGGGGCGGGG	chr1:33182128-33182146	absent	55223	Promoter
GGGGTGGGGGGGGCGGGG	chr1:42731825-42731842	present	149461	Downstream
GGGGAGGGCAGGGCGTGGG	chr21:40155906-40155924	absent	100616148	Intron
GGGGCGGGGCCGGGGCGGG	chr21:42975112-42975130	present	5316	Promoter
GGGCGGGGAGGGGAGGGG	chr22:27554681-27554698	present	4330	Intergenic
GGGGGGGGCGGGGCGGGG	chr22:33528130-33528147	present	9215	Intron
GGGGCGGGGCGGGGCAGGG	chr22:44891552-44891570	present	23779	Intron
GGGGAGGGCTGGGCTGGGG	chr22:44916501-44916519	present	112885	5' UTR
GGGAGGGGCAGGGCGGGG	chr2:11130389-11130406	present	285150	Promoter
GGGAGGGGAGGGGCGGG	chr2:131005683-131005699	present	50649	Intron
GGGACGGGGCGGGGCGGG	chr2:197785363-197785380	present	66037	Promoter
GGGGTGGGGCGGGGCGGGG	chr2:231683502-231683520	present	5757	Intergenic
GGGGAGGGGGCGGGGCGGG	chr2:44778533-44778551	present	79823	Intergenic
GGGGAGGGCGGGTGCTGGG	chr3:127140774-127140792	present	285311	Intergenic
GGGAGGGGAGGGGCGGGG	chr3:13018647-13018664	present	9922	Intron
GGGAGGGGAGGGGCAGGG	chr4:1766503-1766520	absent	10460	Intergenic
GGGCGGGGCGGGCGGGG	chr4:2418706-2418722	absent	57732	Promoter
GGGGAGGGGCAGGGCTGGG	chr4:3779401-3779419	present	152	Intergenic
GGGCGGGAAGGGGCGGGG	chr4:41360750-41360767	present	22998	Promoter
GGGAGGGGCAGGGGCGGGG	chr4:6782547-6782565	absent	9778	Promoter
GGGCGGGGAGGGGGCGGGG	chr5:179795444-179795462	present	4056	Promoter
GGGGAGGGGCAGGGCGGGG	chr5:2011684-2011702	present	101929081	Intergenic
GGGATGGGGAGGGGCGGGG	chr5:72299335-72299353	present	23107	Intron
GGGAGGGGAAGGGGCGGGG	chr6:34223494-34223512	present	3159	Intergenic
GGGGAGGGGAGGGCGCGGG	chr7:148884873-148884891	absent	2146	Promoter
GGGGAGGGGGGGGCGGGG	chr7:151520014-151520031	present	6009	Promoter
GGGCGGGGAGGGCCGGGG	chr7:1669902-1669919	present	392617	Intergenic
GGGCAGGGCGGGGCGGG	chr7:2096544-2096560	present	8379	Intron
GGGTAGGGGCGGGGCGGGG	chr7:75410724-75410742	absent	378108	3' UTR
GGGCGGGGAAGGGCGGGG	chr7:7566848-7566865	absent	54468	Promoter
GGGCGGGGCGGGGCCGGG	chr7:99375518-99375535	absent	10095	Promoter
GGGGAGGGGTCGGGGGGG	chr8:133453251-133453268	present	6482	Downstream
GGGAGCGGGGCGGGGCGGG	chr8:26383646-26383664	present	665	Promoter
GGGAAGGGGCAGGGCGGGG	chr9:1046289-1046307	present	102800446	Promoter
GGGGGGGCGGGGCGGG	chr9:113012640-113012655	present	169834	Promoter
GGGGCGGGGCGGGGCCGGG	chr9:124853507-124853525	absent	401551	Promoter
GGGCGGGGAGGGCCGGG	chr9:133557493-133557509	present	9719	Intron
GGGGCGGGGTCGGGGCGGG	chr9:5629030-5629048	present	57589	Promoter
GGGGCGGGCCGGGGCGGG	chr9:91423955-91423972	present	4783	Promoter
GGGGCGGGCGCGGGGCGGG	chrX:153411372-153411390	present	139735	Intergenic
GGGCGGGGCGGGGCGAGGG	chrX:154546963-154546981	present	2539	Promoter
GGGCGGGCCGGGGCGGGG	chrX:155458643-155458660	absent	8263	Promoter
GGGGAGGGGCGGGGCGGGG	chrX:76427616-76427634	present	57692	Promoter

Supplemental Table S3. Sequence repeats capable of forming multiple G4 structures.

Location	Gene ID	Gene Symbol	Sequence
chr4:1318076-1318118	10296	MAEA	GGGGGAAGCCGGGCACACGGGGCCAGGGAGGCGGGTGGGTGGG
chr4:2756374-2756472	79155	TNIP2	GGGCGGGGCTGCGCGGGGAAGGGCGGGGCTGCGCGGGGGGCGGGGCTGCGTGGGGGAGGGCGGGGCCGGGCTGTTGT
			GGGTGGGCGGGGCGCACCGGGG
chr4:49572959-49573006			GGGGGGTGGGGAGGGTTGGGGGGATTAAGGGGTGGGGAGGGTTGGGGG
chr5:1295088-1295155	7015	TERT	GGGGAGGGGCTGGGAGGGCCCGGAGGGGGCTGGGCCGGGGACCCGGGAGGGGTCGGGACGGGGCGGGG
chr5:2204989-2205021			GGGAAGTGGGGGTGGGGCTGGGGCTTGGGCGGG
chr7:101017720-101018062	102724094	LOC102724094	GGGGGAAGGGAAGGGGTCCCAGGGGGAGGGAGGGAGTCCCAGGGGGAAGGGAAGGGGTCCCAGGGGGAGGGAGGGA
	140453	MUC17	GTCCCAGGGGGAAGGGAAGGGGTCCCAGGGGGAGGGAGGGAATCCCAGGGGGAAGGGAAGGGGTCCCAGGGGGAGG
	10071	MUC12	GAGGGAGTCCCAGGGGGAAGGGAAGGGGTCCCAGGGGGAGGGAGGGAGTCCCAGGGGGAAAGGAAGGGGTCCCAGG
			GGGAGGAAGGGAGTCCCAGGGGGAAGGGAAAGGGAAGGGGTCCCAGGGGGAAGGGAGGGAGTCCCAGGGGGAGAAG
			GGAGTCCCAGGGGGAGAAGGGAGTCCCGGGGGAAGAGGG
chr7:129978853-129978883		RP11-306G20.1	GGGGGTGGGGGTGGGTGTGGGAGGGCAGGGG
chr7:149018792-149018849	9601	PDIA4	GGGGTGGGGGTGGGGGGGTAGGAGGGGGGAGTAGTGGGGTGGGCAGGGTGGGTTGGGG
chr7:26864641-26864689	8935	SKAP2	GGGCCGGGGCGGGGAGATGGGTGGGAAGGGACACGAAGGGCCTGAGGGG
chr7:44224942-44224975	816	CAMK2B	GGGAGGGGCTGGGCAGGGCTGGGAAAGGGGTGGG
chr7:74218035-74218071	7462	LAT2	GGGGGCTGGGGGTGGGCAGGGCCTGAGGGGAGAGGGG
chr8:142348488-142348542	203062	TSNARE1	GGGGCAGCCTGGGGCGGGAGCGGGGGCCAGGGGAGGGTGGGCATGGGGTGCCGGG
chr8:142348556-142348610	203062	TSNARE1	GGGGCAGCCTGGGGCGGGAGCGGGGGCCAGGGGAGGGTGGGCATGGGGTGCCGGG
chr8:144465691-144465773	90990	KIFC2	GGGGAGAAGGGCCGGGGCGGGGCTGCGAGGGGCGGGGTCTGGGCGGGGCTGCGAGGGGCGGGGGGTCTGGGCGGGG
	50626	CYHR1	CTGAGGG
chr9:32956186-32956230			GGGAGGGTCCGGGAAGGGTCCCTGGGTGGGGGAGGGGGAAAGGGG
chr9:39145609-39145636	79937	CNTNAP3	GGGGGCTGGGGGCATGGGGAGGGTAGGG
chr9:5840502-5840539			GGGGGCCGGGGAGGGGCAGTGGGCATGGGTAGGGAGGG
chr9:93210282-93210324	65268	WNK2	GGGGTGAGGGATGGGCAGGGTGGGCAGGGATGGGGGACTGGGG
chrX:107610224-107610250			GGGGGGCAGGGGCAGGGAAGGGGAGGG
chrX:12711746-12711782	9758	FRMPD4	GGGGACTTGGGGCGGGGGGCAGGGTTGGGGGGAAGGG
chr1:11971187-11971260	5351	PLOD1	GGGGCAGGGGGATGGGGTGGGAGGGGTAGGGTGGAGTGGGGGGCTTGGGTGGAAGGGCCAGGGGTGGGTGGGGG
chr1:38005556-38005593	2275	FHL3	GGGCTGGGGGGCCGGGGGCGGGGTCCGGGCGGGGCGGG
	51118	UTP11	
chr10:131738939-131738977			GGGGTGGGTGGGGAGGGAGCATGGGGTGGGCAGGGTGGG
chr10:44647571-44647613			GGGTGAGGGGGATGGGTGGGGGATGGGCAGGGTAGGGCAGGGG
chr10:86347049-86347092	2894	GRID1	GGGTGGGTGGGGCAGGGCAGGAGGGTGGGGCTGGGCAGTTAGGG
chr11:119695264-119695328	5818	NECTIN1	GGGGACTGGTGGGGAGGGTGGGGACCTGGGAGGGGTGGGAGAGGGAGATGGGAATATGGGCAGGG
chr11:63562894-63562932	54979	HRASLS2	GGGAGGGATTAGCTGGGGAGGGAGGGTCCAGGGAAAGGG
chr12:123034471-123034509	57605	PITPNM2	GGGTCGTGGGGCAAGGGTGGGTCATGGGGTGAGGGTGGG
chr12:47939348-47939389	7421	VDR	GGGTGGGGCTTGGGGGAGGTGGGTCCTGGGGTGGGGATGGGG
chr12:53499343-53499404	6895	TARBP2	GGGGGGGTGGGGGGCAGGGATGGGTCTGGGTCCTGGGATCCGGGCGTGGAGGGAGGGTCGGG
	7786	MAP3K12	
chr14:23386616-23386678	4624	MYH6	GGGAGGCCTGGGAAGGGGTGGGGCGAGGGCGGGCAGACAGGGCACAGGGCAGGGTTGAGAGGG
chr16:3957697-3957730	115	ADCY9	GGGATGGGGGTCCTGGGAGGGCAGGGCTAGGGGG
chr17:50604073-50604120	8913	CACNA1G	GGGGGTGGGGAGCAGGGTCAAGGGACAAGGGAGGGTCTGGGCTGGGGG
chr17:82698358-82698455	10966	RAB40B	GGGTGAGCGCGGGCGGAGGGCGTGCCGGGGTGCGGGCGCGGGGCCGGGGAGGGGCGCGGGGCTGGGGAGGGGGTGC
			GGGTGGGGGTCCGGGTCCGGGG

Supplemental Table S3. (Continued).

Location	Gene ID	Gene Symbol	Sequence
chr18:79395460-79395704	4772	NFATC1	GGGGGGGCGCACGGGGAGGGGGGGGCGCACGGGGAGGGGGGGCGCCCGGGGAGGGGGGCGCCCGGGGAGGGGGGGCGCACGGGGAGGGGGGCGCACGGGGAGGGGGGGCGCACGGGGAGGGGGGCGCACGGGGAGGGGCGCACGGGGAGGGGATGGGGGCGTAGGGGCGGGAACGGGGAATCCGGGGGCCGGGCAGGGGGGCGCTGGGGCTGGCGGGGGAAACGGGGGCGAACGGGCCAGACGGG
chr18:79617457-79617512			GGGGGGCGGGGTCCTGGGGGGGCAGGGTCCCGGGGAGGGCGGGGTCCCGGGGAGGG
chr19:6373235-6373294	84266	ALKBH7	GGGGTTGTGGGGCCAGGGGGGTCGGGCGCAGGGATGGGGCGGGGCCACGCTGGGGCGGGG
chr19:9851039-9851068			GGGGGTGAGGGGGTGGGTGGGAGGTGAGGG
chr20:1225680-1225711	642636	RAD21L1	GGGACCGGGGGCAGGGGGCGGGGAAGGGCGGG
chr21:40006234-40006276			GGGCCTGGGGAGAGGGAGGGCCTGGGGAGAGGGAGGGACTGGG
chr21:45287489-45287569	23275<br>642852	POFUT2<br>LOC642852	GGGGCAGGGGCCAGGGGGATGGGATGGAGCGGGGTCAGGGGGCAGGGGTCAGGGGAATGGGATGGGGTCAGGGTTCTGGGG
chr22:43280423-43280510	101927447<br>80274	Z99756.1<br>LOC101927447<br>SCUBE1	GGGTGAGGGGAAGGGACGGGGATGGGTGAGGGGAAGGGACGGGAGGATGGGTGAGGGGAAGGGACAGGGGGATGGGTGAGGGGAAGGG
chr22:49037526-49037614			GGGAGCGGGAGGGGCCAGGGGGTGGGACGGGGCGGGGAGAGGGAGAAGAGGGGTTCTGGGTGGGAAGGATTGGGGAGCGGGAGGAGGGG
chr3:129606851-129606910	23129	PLXND1	GGGCGGCCAGGGGCAGGCGGGGGTCCCGGGGGCGGGCGGGGCCGGGGCGGGGAGTGAGGG
chr4:88699286-88699364	8916<br>266812	HERC3<br>NAP1L5	GGGCAGGGTGGGGAAGAAGAGGGTGGGGCTGCGTGGGTGGGTGGGGAGGAAGAGGGTGGGGCTGGGTGGGTGGGTGGGG
chr5:524556-524624	6550	SLC9A3	GGGCTCCGGGGGAGGGTGGGCACCAGGGAGCGCGGGGTGGGCGTGCGCGGGCGGGGCGGGCGTGCCGGG
chr6:34486149-34486233	29993	PACSIN1	GGGGGTGAGGGGTGGAGGGACAGGGGGCCTGGGAACCCAGGGAGAGGGAGGCAGGGCCTAGGGGTGGGGTGAGGTGGGTTTGGGG
chr6:44222239-44222286	2030	SLC29A1	GGGCTGGCGGGGATGTGGGGGATGGGGGTGGGGTGGGGGAGGGTTGGG
chr7:157138531-157138583	9690	UBE3C	GGGCCGGGATGGGGTGCAGGGCAGGGTGCGGGGTGCAGGGTGGGGTGCAGGGG
chr8:142348624-142348668	203062	TSNARE1	GGGGCGGGAGCGGGGGCCAGGGGAGGGTGGGCATGGGGTGCCGGG
chr8:142464060-142464098	575	ADGRB1	GGGGGCAGGGAGGCGGGGCAAGGGTGGGATGGGAGAGGG
chr8:26383526-26383612	665	BNIP3L	GGGCGGGGCGGGGCGGGGGCGGGCCTGGGGGGCGGGGAGGCCGGGTGGGCGGAGCGGGCCGCGGAGGGGACGTGGGCCGGGATGGGG
chr9:132589622-132589662	56751	BARHL1	GGGGGGCACTGGGCTGGGGCGCCAGGGAGGGCCGGGCAGGG
chr9:136800509-136800550	84960<br>55684	RP11-216L13.19<br>CCDC183<br>RABL6<br>RP11-216L13.18	GGGAAGGGCGGGGGTCAGGGGCTGGGATCTGGGAGGGGCGGG
chrX:120157742-120157823	727940	RHOXF2B	GGGGTGGGGGGAGTAGGGCGGGGGAGGGAGTAGGGCGGGGGGGCGTAGGGTGGAGGGGGGGAGTAGGGCGGGGGGGCCGGGG
chr1:2640997-2641101	100287898	TTC34	GGGGCAGGGAAGCGGGGTGTGGGGAGGGCAGGGAAGGGGGGTGTGGGGAGGGCTGGGAAGGGAGGTATGGGGAGGGCTGGGAAGGGAGGTATGGGGAGGGCTGGG
chr1:29727790-29727836			GGGTGGGCTTGGGGAGGGGTGGAGGGAAGGGTGGGCTGAGGGAGGGG
chr14:103109168-103109254	91828	EXOC3L4	GGGAGGGAGACACGGGGACAGGGTGGAGAGGGAGGGAGGGAGGGAGACATGGGGACAGGGTGGCGAGGGAGAGAGGGAGACCGGGGG
chr14:103109565-103109722	91828	EXOC3L4	GGGAGACCCAGGGACAGGGTGGAGAGGGAGGGAGGGAGGGAGACATGGGGGCAGGGTGGAGAGGGAGGGAGGAGGGAGACATGGGGGCAGGGTGGAGAGGGAGGGAGGAGGGAGACATGGGGGCAGGGTGGAGAGGGAGGGAGGAGGGAGACATGGGG
chr16:47887333-47887374	101927132<br>100507534	RP11-523L20.2<br>LINC02133<br>LINC02192	GGGTCACAGGGTCAGGGAGGGGGCTGCGGGTGGGAGGCAGGG

Supplemental Table S3. (Continued).

Location	Gene ID	Gene Symbol	Sequence
chr19:38390491-38390536	399473	SPRED3	GGGGCATGCGGGGAGGGTAGGGACCTGGGGAGGGAGGGGAGAGGGG
	199720	GGN	
chr2:129877629-129877660			GGGGAGAGGGGCGGGGGCGGGGCCGGGCCGGG
chr21:43075944-43076011	875	CBS	GGGGTGGGGAAGGGGTGGGGGGGAGGGGCCCGGGCTGGGTGGGGTGGAGGAGGGGCTGGGGGGGCGGG
chr22:44939685-44939728	112885	PHF21B	GGGGATGGGTGGGAGCAGGGCTAGGGAGGGGCGAAGGGATAGGG
chr3:50204812-50204840	10991	SLC38A3	GGGGTGGGGTGGGGGCGAGGGTGGGAGGG
chr8:10852146-10852235			GGGAGGGGAAGGGGAGGGCAGGGAAGAGAAGGGTCAGCACGGGGAAGAGAAGGGGAGCGCGGGGAAGGGAAGGGTCAGCGCGGGGAAGGG
chr9:127809879-127809969	2356	FPGS	GGGGCGGGATCTTGGGGAAGGGCGGGGCGGGGTCGTGGGGAAGGGCGGGGGCGGGCCCATGGGGAGGGGCGGGGTCGTGGGCGGGGACGGG
chr9:135757071-135757140	57582	KCNT1	GGGGTATCAGCGGGGGCGATGGAGGGTGGGGGTGGGGCCCAGCAGGGGAGGGGCAGGGTGGGGAAGAGGG
chrX:154065081-154065110	4204	MECP2	GGGGTGGGTGGGGTGGGGGCCGGGGAAGGG
chr5:181098279-181098403			GGGGGAAGGGACTGGAGGGGAGGGGAGGGGAGGGGACGGGTGGGGAGGGGTGGGGCGGGGAGGGAAAGGGTGGGGAGCAGAGGGGTGGGGAGGGGAGGGGTGGGGAGAGGTAGGGAGGGGAGGGG
chr7:76282546-76282601	222183	SRRM3	GGGGGCTGGGGGCGGGGAGGGTTCCCTGGGGCGGGGCTTAGGGCGGAGGGGCGGGG

Supplemental Table S4: Summary of Family 2 G4 sequences.

Sequence	Location	Experimental Evidence	Gene ID	Anntation
GGGGAGGGCCTGGGACAGGG	chr11:1648838-1648857	absent	387742	Intergenic
GGGAGGGCCTTGGGACAGGG	chr1:226482764-226482783	absent	375057	Intergenic
GGGAGGGGCCTGGGACAGGG	chr9:135803176-135803195	present	57582	Intergenic
GGGGGAATGGGCTGGGACAGGG	chr1:7715171-7715192	absent	23261	Intron
GGGAAGGGGGCTGGGAAAGGG	chr17:74059333-74059353	absent	6169	Intron
GGGCTGGGCATGGGACAGGG	chr14:74084190-74084209	present	4329	Promoter
GGGGAGTGGGCTGGGACAGGG	chr1:120652107-120652127	absent	101954277	Intergenic
GGGGAGTGGGCTGGGACAGGG	chr1:149272171-149272191	absent	400818	Intergenic

Supplemental Table S5. Summary of Family 3 G4 sequences.

Sequence	Location	Experimental Evidence	Gene ID	Annotation
GGGAGGGGGCTGCAGGGAGCTGGG	chr19:41700278-41700301	present	1087	Intron
GGGAGGGGGCTGCAGGGAGCTGGG	chr19:41700314-41700337	present	1087	Intron
GGGAGGGGGCTGCAGGGAGCTGGG	chr19:41700350-41700373	present	1087	Intron
GGGAGGGGGCTGCAGGGAGCTGGG	chr19:41700386-41700409	present	1087	Intron
GGGAGGGGGCTGCAGGGATGGGGG	chr12:124531250-124531273	present	9612	Promoter
GGGAGGGGGCTGCAGGGATGGGGG	chr3:53193909-53193932	absent	5580	Intergenic
GGGAGGGGCTGCAGGGATGGGGG	chr22:43426947-43426969	absent	758	Intron
GGGAGGGGGAGGCAGGGTTGGGG	chr1:206041760-206041782	absent	440712	Intron
GGGAGGGTGCTCCTGGGATGGGG	chr17:1312485-1312507	present	286753	Intergenic
GGGAGGGGGCTTCTGGGGTGGGG	chr3:13852428-13852450	present	7476	Intron

Supplemental Table S6. Summary of Family 4 G4 sequences.

Sequence	Location	Experimental Evidence	Gene ID	Annotation
GGGCCTGGGAGGGAAGGAGAGGG	chr4:3513681-3513703	absent	4043	Intron
GGGCTAGGGTCGGGAGTAGAGGG	chr2:88972574-88972596	absent	100616399	Intron
GGGGCTGTGGAGGGAGGGAGAGGG	chr15:41893960-41893983	absent	51332	Promoter
GGGCTGGGGCGGGAAGGAGAGGG	chr1:121185193-121185215	absent	653464	Promoter
GGGCTGGGGCGGGAAGGAGAGGG	chr1:143972835-143972857	absent	554282	Promoter
GGGGCTGGGGCGGGAAGGAGAGGG	chr1:206203718-206203741	present	729533	Promoter
GGGCAGGGCGAGGGATGGAGAGGG	chr17:39144386-39144409	absent	57125	Intron
GGGCATGGGCGGGGTGGAGAGGG	chr3:143313988-143314010	absent	100885796	Intron
GGGGTGGGGAGGGAATGTGAGGG	chr10:70886991-70887013	absent	5092	Promoter

Supplemental Table S7. Summary of Family 32 G4 sequences.

Sequence	Location	Experimental Evidence	Gene ID	Annotation
GGGAAGGGGAAGGGACAGGG	chr1:1136393-1136412	present	254099	Promoter
GGGGTGGGGTGGGGAGAGGG	chr1:161197724-161197743	present	4720	Promoter
GGGCTGGGGTTGGGGCTGGG	chr1:201275493-201275512	present	5317	Intergenic
GGGCTGGGGCTGGGGCAGGG	chr1:229251426-229251445	absent	5867	3' UTR
GGGGTGGGGTGGGGGATGGG	chr1:3110907-3110926	present	63976	Intron
GGGCTGTGGGCGGGGCTAGGG	chr1:37735516-37735536	present	284656	Promoter
GGGCTGGGAGAGGGCCTGGG	chr1:54138165-54138184	absent	200008	Intron
GGGATGGGCATGGGGGAGGG	chr10:124644752-124644771	present	64077	Intron
GGGGTGGGGGTGGGGTTGGG	chr10:130945693-130945712	present	100422867	Intergenic
GGGATGGGCTGGGGGCTGGG	chr10:27715853-27715872	present	283078	Intron
GGGGTGGGGTGGGGGCAGGG	chr10:69897023-69897042	present	1305	Intron
GGGATGGGGCATGGGGAGGG	chr10:79375641-79375660	present	10105	Intergenic
GGGCAGGGGGTGGGGCAGGG	chr11:404765-404784	present	11187	Promoter
GGGCAGGGGGAGGGGGAGGG	chr11:64934344-64934363	absent	170589	Promoter
GGGGTGAGGGTGGGGGTGGG	chr12:129184860-129184879	present	101927735	Intron
GGGGTGGGGGATGGGGAGGG	chr12:51905879-51905898	present	94	Promoter
GGGGTGGGGGAGGGGCAGGG	chr13:111296247-111296266	present	8874	Intron
GGGCAGGGCTGGGTGGAGGG	chr13:31009892-31009911	absent	122046	Intergenic
GGGCAGGGGTTGGGTGAGGG	chr14:24147068-24147087	present	5721	Promoter
GGGTTGGGGGCGGGGGTGGG	chr14:65413539-65413558	present	2530	Promoter
GGGGTGTGGGTGGGGCAGGG	chr14:95332851-95332870	present	101929080	Promoter
GGGGAGGGGTGGGGACCGGG	chr14:96219408-96219427	present	623	Intron
GGGATGGGGAGGGGACAGGG	chr15:77583296-77583315	absent	84894	Intron
GGGCTGGGGAGGGGACAGGG	chr15:85258124-85258143	absent	11214	Intergenic
GGGGAGGGCTGGGACCAGGG	chr15:90884045-90884064	present	2242	Promoter
GGGCTGGGACAGGGCCAGGG	chr16:32301164-32301183	absent	729264	Intergenic
GGGCAGGGCTGGGTCCAGGG	chr16:32302937-32302956	absent	729264	Intergenic
GGGCTGGGACAGGGCCAGGG	chr16:33507434-33507453	absent	24150	Intergenic
GGGCAGGGCTGGGTCCAGGG	chr16:33509201-33509220	absent	24150	Intergenic
GGGGAGGGCATGGGGCAGGG	chr16:46623763-46623782	present	79801	Promoter
GGGGTGGGGTTGGGGGAGGG	chr16:54970795-54970814	present	10265	Intergenic
GGGCAGGGCTGGGAGAAGGG	chr17:10232070-10232089	absent	8522	Intergenic
GGGAAGGGGAGGGGTCTGGG	chr17:58116701-58116720	absent	140735	Intergenic
GGGCTGGGCCTGGGCCTGGG	chr17:61402103-61402122	absent	6909	Promoter
GGGCGGGGCTGGGTCCTGGG	chr17:76079074-76079093	present	353174	Promoter
GGGGTGGGGGTGGGGATGGG	chr17:77413847-77413866	present	10801	Intron
GGGCTGGGGGAGGGGGTGGG	chr17:82435883-82435902	present	284004	Promoter
GGGGTGGGGTGGGTGGAGGG	chr17:8743246-8743265	absent	146849	Promoter
GGGCAGGGGCCTGGGGAGGG	chr18:10168387-10168406	present	9218	Intergenic
GGGGGGGGGGTGGGGGTGGG	chr18:22548091-22548110	present	64693	Intergenic
GGGCCCAGGGTGGGGCAGGG	chr18:37415961-37415980	present	56853	Intron
GGGCCGGGGCTCTGGGCGGG	chr18:62596252-62596271	absent	54877	Intergenic
GGGGTGGGATGGGGGCTGGG	chr19:17101093-17101112	present	4650	Promoter
GGGCAGGTGGGTGGGCAGGG	chr19:42351769-42351788	absent	102465875	Promoter
GGGAGGGGCGAGGGCCAGGG	chr19:51421895-51421914	present	89790	Promoter
GGGTTGGGGGTGGGGGAGGG	chr2:10264610-10264629	present	3241	Intergenic
GGGAAGGGGGTGGGAGAGGG	chr2:205734541-205734560	absent	8828	Intron
GGGCAGGGACATGGGGTGGG	chr2:233976450-233976469	present	79054	Promoter
GGGGTGAGGGTGGGGGTGGG	chr20:32612791-32612810	present	149950	Intergenic
GGGTTGGGGGAGGGGGTGGG	chr20:32980815-32980834	present	140732	Downstream
GGGGTGGGACAGTGGGAGGG	chr21:40286094-40286113	present	1826	Intron
GGGTGGGGCTAGGGCCAGGG	chr22:21613160-21613179	absent	150223	Intron
GGGGTGGGAGTGAGGGTGGG	chr3:11222231-11222250	present	3269	Promoter
GGGCTGGGGCTGGGCCAGGG	chr3:125190497-125190516	absent	84561	Promoter
GGGCTGGGGCAGGGGCCGGG	chr3:127823053-127823072	absent	11343	Promoter
GGGGAGGGCATGGGGCAGGG	chr3:129088461-129088480	absent	2815	3' UTR
GGGCTGGGGGAGAGGGTGGG	chr3:129348117-129348136	present	339942	Intergenic
GGGGGGGGGTGGGGGCAGGG	chr3:131466690-131466709	present	11222	Promoter
GGGAGGGGCTGGGGCCTGGG	chr3:13628839-13628858	absent	2199	Promoter
GGGCAGGGCTCGGGACAGGG	chr3:42732393-42732412	absent	100874114	Promoter
GGGTAGGGAAAGGGAAAGGG	chr3:45979832-45979851	absent	79443	Intron
GGGGTGGGGTAGGGGGAGGG	chr3:49489904-49489923	present	1605	Promoter
GGGGTGGGGTGGGGGATGGG	chr3:49642027-49642046	present	8927	Promoter
GGGGTGGGTGCGGGGCAGGG	chr4:102826891-102826910	present	7323	Promoter
GGGGTGGGGAGGGGGCTGGG	chr4:141929124-141929143	present	3600	Intergenic
GGGCAGGGGAATTGGGTGGG	chr4:152304991-152305010	absent	55294	Intergenic

Supplemental Table S7. (Continued).

Sequence	Location	Experimental Evidence	Gene ID	Annotation
GGGCAGGGGTGGTGGGTGGG	chr5:113656482-113656501	absent	64848	Intergenic
GGGGTGGGGCTTGGGGAGGG	chr5:134151255-134151274	present	6932	3' UTR
GGGGTGTGGGCGGGGCAGGG	chr5:151772043-151772062	present	10146	Promoter
GGGCTGGGGCTAGGGGCGGG	chr5:168410001-168410020	present	23286	Promoter
GGGCAGGGGCAGGGTGAGGG	chr6:157921891-157921910	absent	51429	Intron
GGGCTGGGGCAGGGGGAGGG	chr6:166558569-166558588	absent	6196	Intron
GGGCAGGGGGTGGGGGAGGG	chr7:128832311-128832330	absent	2318	Promoter
GGGAGGGGCTGGGGGCTGGG	chr7:30915933-30915952	absent	358	Promoter
GGGAGGGGCCGGGAGCTGGG	chr7:74454399-74454418	absent	9569	Promoter
GGGGTGGGGGCGGGGGAGGG	chr8:113439774-113439793	present	114788	Promoter
GGGCAGGGGTGGGGGGAGGG	chr8:127255311-127255330	present	100507056	Intergenic
GGGAGGGGTTGGGGGCTGGG	chr8:144496661-144496680	present	84988	Promoter
GGGCTGTGGGCGGGCCAGGG	chr8:144502891-144502910	absent	2875	Promoter
GGGGTGGGGGAGGGGTTGGG	chr8:25517201-25517220	present	157313	Intergenic
GGGGTGGGAGTAGGGGAGGG	chr8:38451245-38451264	present	2260	Intron
GGGGTTGGGGTGGGGGAGGG	chr8:54466113-54466132	present	64321	Intergenic
GGGCTGGGGTTGGGGAGGGG	chr8:99974271-99974290	present	26166	Promoter
GGGATGGGCTTGGGCCTGGG	chr9:113536716-113536735	absent	5998	Promoter
GGGCAGAGGGTGGGGCAGGG	chr9:121763670-121763689	absent	153090	Promoter
GGGGTTGGGTGGGGGCTGGG	chr9:124506593-124506612	present	2516	Promoter
GGGCAGGGGACGGGGGTGGG	chr9:27312142-27312161	absent	54586	Intergenic
GGGCTGGGGTCGGGGTGGGG	chr9:84670271-84670290	present	4915	Promoter
GGGATGGGGAGGGAACAGGG	chrX:18891734-18891753	present	100132163	Promoter
GGGCTGGGGCAGGGATAGGG	chrX:19081377-19081396	absent	10149	Promoter

Supplemental Table S8. Summary of Family 75 G4 sequences.

Sequence	Location	Experimental Evidence	Gene ID	Annotation
GGGGGAGGGAGGGCCTGGG	chr11:19817544-19817562	present	89797	Intron
GGGGGTGGGAGGGGCAGGG	chr11:65967601-65967619	present	9092	Intron
GGGTGGAGGGAGGGCTGGG	chr12:130663161-130663179	absent	23504	Intron
GGGGGTGGGGGGGCCTGGG	chr12:56096956-56096974	present	2065	Promoter
GGGGGTGGGAGGGCAGGG	chr16:78000971-78000988	present	10143	Intergenic
GGGGTGGGAGGGGCATGGG	chr17:41888505-41888523	present	47	Intron
GGGGGTGGGAGGGCATGGG	chr19:52643765-52643783	present	55769	Intron
GGGGGTGGGAGGGCATGGG	chr19:52700738-52700756	present	55769	Downstream
GGGGGTGGGAGGGCATGGG	chr19:52762022-52762040	present	162966	Downstream
GGGGGTGGGAGGGCATGGG	chr19:52818986-52819004	present	7576	Promoter
GGGGGTGGGAGGGCATGGG	chr19:52855275-52855293	present	7576	Promoter
GGGGGTGGGAGGGCATGGG	chr19:52938557-52938575	present	388559	Intron
GGGGGTGGGAGGGCACGGG	chr19:53129043-53129061	present	55786	Promoter
GGGTGGTGGGAGGGATGGG	chr1:18332823-18332841	absent	84966	Intron
GGGGGTGGGAGGGCCTGGG	chr20:63293908-63293926	present	57642	Promoter
GGGGGTGGGTAGGGCCGGG	chr2:219060082-219060100	present	3549	Promoter
GGGGAGAGGGAGGGCCGGG	chr4:40244011-40244029	absent	399	3' UTR
GGGGGAGGGAGGGCTTGGG	chr8:124855332-124855350	present	157381	Intron

Supplemental Table S9. Summary of Family 80 G4 sequences.

Sequence	Location	Experimental Evidence	Gene ID	Distance To TSS	annot
GGGGCGGGCTGGGGGCGGGG	chr11:67630369-67630388	present	254552	-439	Promoter
GGGTCGGGGCCGGGGGAGGG	chr11:968778-968797	present	161	-16587	Intron
GGGGCGGGCCTCGGGGGCGGGG	chr14:93184925-93184946	present	64112	0	Promoter
GGGTGCGGGGCCGGGGGGAGGGG	chr17:39927095-39927117	absent	94103	112	Promoter
GGGGCTGGGGGCGGGGGCGGG	chr17:79836273-79836293	present	8535	1588	Promoter
GGGGCGGGGCCGGGGGCGGG	chr19:18097748-18097767	present	23031	-26	Promoter
GGGGCGGGTCGTGGGCGGGG	chr19:2096722-2096741	present	126308	-49	Promoter
GGGGCTGGGTCGGGGGCGGGG	chr19:55081615-55081635	present	54869	-57	Promoter
GGGGCGGGCCCAGGGGCGGG	chr1:16700286-16700305	present	100500876	19031	Intergenic
GGGGCGGGCCGGGGGGAGGGG	chr1:185411921-185411941	present	100288079	-76882	Intergenic
GGGGCGGGCCGGGGGCGGGG	chr20:5001518-5001537	present	9962	-19	Promoter
GGGGGCGGGCTCGGGGGCGGGG	chr21:5022922-5022943	present	23308	389	Promoter
GGGGCGGGGCACGGGGGAGGG	chr4:1011532-1011552	present	53834	-270	Promoter
GGGGCGGGACCGGGGAGAGGGG	chr6:11043967-11043988	present	100506409	207	Promoter
GGGGGGGGTAGTGGGCGGGG	chr7:156169292-156169311	present	389602	206660	Intergenic
GGGGCGGGCCGTGGGCCGGG	chr7:158829641-158829660	absent	57488	-13	Promoter
GGGGCAGGCCGGGCGGGAGGGG	chr7:20798511-20798532	absent	221833	-11625	Intergenic
GGGGCGGGGGCCGCGGGCGGG	chr7:6374902-6374922	absent	5879	339	Promoter
GGGGCCGGGGCCGGGGCCGGG	chr8:22049068-22049088	absent	2039	-61	Promoter
GGGGCGGGCTCGGGGGCGGGG	chr8:66962618-66962638	present	100129654	-28	Promoter
GGGGCCGGGCCGAGGGGCGGG	chr9:132241390-132241410	present	84628	-31	Promoter

Supplemental Table S10. Enriched GO:BP categories for Family 4.

GO Term	GO Term Name	P Value	ADJ P Value
GO:0021815	modulation of microtubule cytoskeleton involved in cerebral cortex radial glia guided migration	2.13E-05	2.13E-05
GO:0021816	extension of a leading process involved in cell motility in cerebral cortex radial glia guided migration	2.13E-05	2.13E-05
GO:0021814	cell motility involved in cerebral cortex radial glia guided migration	8.51E-05	8.51E-05
GO:0022030	telencephalon glial cell migration	0.000382	0.000382
GO:0021801	cerebral cortex radial glia-guided migration	0.000382	0.000382
GO:0021799	cerebral cortex radially oriented cell migration	0.000389	0.000389
GO:0031269	pseudopodium assembly	0.000552	0.000552
GO:0031268	pseudopodium organization	0.000557	0.000557
GO:0021795	cerebral cortex cell migration	0.00059	0.00059
GO:1904861	excitatory synapse assembly	0.00059	0.00059
GO:1904862	inhibitory synapse assembly	0.00059	0.00059
GO:0022029	telencephalon cell migration	0.000893	0.000893
GO:0021885	forebrain cell migration	0.000899	0.000899
GO:0008347	glial cell migration	0.000983	0.000983
GO:2001222	regulation of neuron migration	0.001145	0.001145
GO:0021987	cerebral cortex development	0.003356	0.003356
GO:0046847	filopodium assembly	0.004232	0.004232
GO:0060996	dendritic spine development	0.004705	0.004705
GO:0021543	pallium development	0.00474	0.00474
GO:0001764	neuron migration	0.007448	0.007448
GO:0021537	telencephalon development	0.009569	0.009569
GO:0007416	synapse assembly	0.0142	0.0142
GO:0030900	forebrain development	0.017572	0.017572
GO:0016358	dendrite development	0.017572	0.017572
GO:0042063	gliogenesis	0.017572	0.017572
GO:0030336	negative regulation of cell migration	0.031913	0.031913
GO:2000146	negative regulation of cell motility	0.034153	0.034153
GO:0050808	synapse organization	0.035988	0.035988
GO:0040013	negative regulation of locomotion	0.035988	0.035988
GO:0034329	cell junction assembly	0.046932	0.046932

Supplemental Table S11. Enriched GO:BP categories for Family 32.

GO ID	GO Name	P Value	ADJ P Value
GO:0031346	positive regulation of cell projection organization	0.017646	0.017646
GO:0035378	carbon dioxide transmembrane transport	0.017646	0.017646
GO:0032989	cellular component morphogenesis	0.023881	0.023881
GO:0048842	positive regulation of axon extension involved in axon guidance	0.023881	0.023881
GO:0048858	cell projection morphogenesis	0.023881	0.023881
GO:0003097	renal water transport	0.023881	0.023881
GO:0051130	positive regulation of cellular component organization	0.023881	0.023881
GO:0051239	regulation of multicellular organismal process	0.023881	0.023881
GO:0048846	axon extension involved in axon guidance	0.023881	0.023881
GO:0120039	plasma membrane bounded cell projection morphogenesis	0.023881	0.023881
GO:1902284	neuron projection extension involved in neuron projection guidance	0.023881	0.023881
GO:1903955	positive regulation of protein targeting to mitochondrion	0.023881	0.023881
GO:0032990	cell part morphogenesis	0.024503	0.024503
GO:1903749	positive regulation of establishment of protein localization to mitochondrion	0.025365	0.025365
GO:0097485	neuron projection guidance	0.029584	0.029584
GO:0007411	axon guidance	0.029584	0.029584
GO:1903214	regulation of protein targeting to mitochondrion	0.032099	0.032099
GO:0007167	enzyme-linked receptor protein signaling pathway	0.032099	0.032099
GO:0048518	positive regulation of biological process	0.032578	0.032578
GO:0051094	positive regulation of developmental process	0.033021	0.033021
GO:0050772	positive regulation of axonogenesis	0.03945	0.03945
GO:0048468	cell development	0.03945	0.03945
GO:1903747	regulation of establishment of protein localization to mitochondrion	0.03945	0.03945
GO:0007409	axonogenesis	0.03945	0.03945
GO:0022603	regulation of anatomical structure morphogenesis	0.039943	0.039943
GO:0048812	neuron projection morphogenesis	0.045216	0.045216
GO:0000902	cell morphogenesis	0.045216	0.045216
GO:0008361	regulation of cell size	0.045216	0.045216
GO:0051347	positive regulation of transferase activity	0.045887	0.045887
GO:0061564	axon development	0.045887	0.045887
GO:0090066	regulation of anatomical structure size	0.046587	0.046587
GO:0120035	regulation of plasma membrane bounded cell projection organization	0.046587	0.046587

Supplemental Table S12. Enriched GO:BP categories for Family 75.

GO ID	GO Name	P Value	ADJ P Value
GO:0045582	positive regulation of T cell differentiation	0.015898	0.015898
GO:0045621	positive regulation of lymphocyte differentiation	0.015898	0.015898
GO:1903708	positive regulation of hemopoiesis	0.016146	0.016146
GO:1902107	positive regulation of leukocyte differentiation	0.016146	0.016146
GO:0045580	regulation of T cell differentiation	0.016146	0.016146
GO:0045619	regulation of lymphocyte differentiation	0.019728	0.019728
GO:0050870	positive regulation of T cell activation	0.03222	0.03222
GO:0030217	T cell differentiation	0.03222	0.03222
GO:1903039	positive regulation of leukocyte cell-cell adhesion	0.03674	0.03674
GO:1902105	regulation of leukocyte differentiation	0.03674	0.03674
GO:0022409	positive regulation of cell-cell adhesion	0.047638	0.047638
GO:1903037	regulation of leukocyte cell-cell adhesion	0.04765	0.04765
GO:0030155	regulation of cell adhesion	0.04765	0.04765
GO:0030098	lymphocyte differentiation	0.04765	0.04765
GO:0050863	regulation of T cell activation	0.04765	0.04765
GO:1903706	regulation of hemopoiesis	0.049277	0.049277

Supplemental Table S13. Enriched GO:BP categories for Family 80.

GO ID	GO Name	P Value	ADJ P Value
GO:0098562	cytoplasmic side of membrane	1.57E-02	1.57E-02
GO:0005886	plasma membrane	1.57E-02	1.57E-02
GO:0009898	cytoplasmic side of plasma membrane	1.57E-02	1.57E-02
GO:0098590	plasma membrane region	1.57E-02	1.57E-02
GO:0031253	cell projection membrane	1.81E-02	1.81E-02
GO:0101003	ficolin-1-rich granule membrane	1.86E-02	1.86E-02
GO:0098552	side of membrane	1.86E-02	1.86E-02
GO:0071944	cell periphery	1.86E-02	1.86E-02
GO:0032587	ruffle membrane	1.86E-02	1.86E-02
GO:0030667	secretory granule membrane	1.93E-02	1.93E-02
GO:0031256	leading edge membrane	3.64E-02	3.64E-02
GO:0005884	actin filament	3.64E-02	3.64E-02
GO:0031234	extrinsic component of cytoplasmic side of plasma membrane	3.64E-02	3.64E-02
GO:0001726	ruffle	3.85E-02	3.85E-02
GO:0016020	membrane	3.85E-02	3.85E-02
GO:0019897	extrinsic component of plasma membrane	4.29E-02	4.29E-02
GO:0031224	intrinsic component of membrane	4.29E-02	4.29E-02
GO:0031227	intrinsic component of endoplasmic reticulum membrane	4.29E-02	4.29E-02
GO:0098797	plasma membrane protein complex	4.29E-02	4.29E-02
GO:0101002	ficolin-1-rich granule	4.29E-02	4.29E-02
GO:0070820	tertiary granule	4.29E-02	4.29E-02

Supplemental Table S14. Enriched GO:BP categories for experimentally validated G4s overlapping enhancers, group 1.

GO ID	GO Name	P Value	ADJ P Value
GO:0048583	regulation of response to stimulus	2.28E-06	2.28E-06
GO:0035556	intracellular signal transduction	2.28E-06	2.28E-06
GO:0010033	response to organic substance	2.36E-06	2.36E-06
GO:0007165	signal transduction	3.86E-06	3.86E-06
GO:1902531	regulation of intracellular signal transduction	3.86E-06	3.86E-06
GO:0009966	regulation of signal transduction	3.86E-06	3.86E-06
GO:0050896	response to stimulus	6.92E-06	6.92E-06
GO:0048584	positive regulation of response to stimulus	6.92E-06	6.92E-06
GO:0007166	cell surface receptor signaling pathway	2.89E-05	2.89E-05
GO:0007154	cell communication	2.89E-05	2.89E-05
GO:0010646	regulation of cell communication	6.71E-05	6.71E-05
GO:1902533	positive regulation of intracellular signal transduction	6.71E-05	6.71E-05
GO:0023051	regulation of signaling	6.71E-05	6.71E-05
GO:0023052	signaling	6.71E-05	6.71E-05
GO:0034097	response to cytokine	6.96E-05	6.96E-05
GO:0051716	cellular response to stimulus	7.39E-05	7.39E-05
GO:0009967	positive regulation of signal transduction	7.42E-05	7.42E-05
GO:0009615	response to virus	0.000118423	0.0001184
GO:0010647	positive regulation of cell communication	0.000218782	0.0002188
GO:0032101	regulation of response to external stimulus	0.000228634	0.0002286
GO:0023056	positive regulation of signaling	0.000228634	0.0002286
GO:0071310	cellular response to organic substance	0.000234694	0.0002347
GO:0019221	cytokine-mediated signaling pathway	0.000236278	0.0002363
GO:0042221	response to chemical	0.000236278	0.0002363
GO:0012501	programmed cell death	0.000236278	0.0002363
GO:0006915	apoptotic process	0.000236919	0.0002369
GO:0009605	response to external stimulus	0.000237548	0.0002375
GO:0044419	biological process involved in interspecies interaction between organisms	0.000270631	0.0002706
GO:0097190	apoptotic signaling pathway	0.000378032	0.000378
GO:0070887	cellular response to chemical stimulus	0.000398413	0.0003984
GO:0051607	defense response to virus	0.000398413	0.0003984
GO:0140546	defense response to symbiont	0.000398413	0.0003984
GO:0007249	I-kappaB kinase/NF-kappaB signaling	0.000398413	0.0003984
GO:1904747	positive regulation of apoptotic process involved in development	0.000693039	0.000693
GO:1902339	positive regulation of apoptotic process involved in morphogenesis	0.000693039	0.000693
GO:0009753	response to jasmonic acid	0.000693039	0.000693
GO:0071395	cellular response to jasmonic acid stimulus	0.000693039	0.000693
GO:0031347	regulation of defense response	0.000693039	0.000693
GO:0071345	cellular response to cytokine stimulus	0.000694499	0.0006945
GO:0048518	positive regulation of biological process	0.001032031	0.001032
GO:0051055	negative regulation of lipid biosynthetic process	0.001298251	0.0012983
GO:0008219	cell death	0.00131134	0.0013113
GO:0080134	regulation of response to stress	0.00131134	0.0013113
GO:0070542	response to fatty acid	0.001354706	0.0013547
GO:1901798	positive regulation of signal transduction by p53 class mediator	0.001354706	0.0013547
GO:0016032	viral process	0.001418243	0.0014182
GO:0002376	immune system process	0.001550915	0.0015509
GO:0043122	regulation of I-kappaB kinase/NF-kappaB signaling	0.001991596	0.0019916
GO:0043207	response to external biotic stimulus	0.002342802	0.0023428
GO:0051707	response to other organism	0.002342802	0.0023428
GO:0038061	NIK/NF-kappaB signaling	0.002342802	0.0023428
GO:0043067	regulation of programmed cell death	0.003172007	0.003172
GO:1903829	positive regulation of protein localization	0.003172007	0.003172
GO:0008630	intrinsic apoptotic signaling pathway in response to DNA damage	0.003495663	0.0034957
GO:1901222	regulation of NIK/NF-kappaB signaling	0.003495663	0.0034957
GO:0045071	negative regulation of viral genome replication	0.003495663	0.0034957
GO:0033209	tumor necrosis factor-mediated signaling pathway	0.003654884	0.0036549
GO:0071398	cellular response to fatty acid	0.003654884	0.0036549
GO:1902337	regulation of apoptotic process involved in morphogenesis	0.003677392	0.0036774
GO:0009607	response to biotic stimulus	0.003677392	0.0036774
GO:1904748	regulation of apoptotic process involved in development	0.003677392	0.0036774
GO:0045833	negative regulation of lipid metabolic process	0.003695766	0.0036958
GO:0016601	Rac protein signal transduction	0.004136778	0.0041368
GO:0072331	signal transduction by p53 class mediator	0.004424419	0.0044244
GO:0042981	regulation of apoptotic process	0.004424419	0.0044244
GO:0006952	defense response	0.004489547	0.0044895

Supplemental Table S14. (Continued).

GO ID	GO Name	P Value	ADJ P Value
GO:0048522	positive regulation of cellular process	0.004536959	0.004537
GO:0071677	positive regulation of mononuclear cell migration	0.004728666	0.0047287
GO:0050789	regulation of biological process	0.005144355	0.0051444
GO:0048525	negative regulation of viral process	0.005144355	0.0051444
GO:0032502	developmental process	0.005423495	0.0054235
GO:0050793	regulation of developmental process	0.005888178	0.0058882
GO:0008625	extrinsic apoptotic signaling pathway via death domain receptors	0.00633763	0.0063376
GO:1902644	tertiary alcohol metabolic process	0.006593339	0.0065933
GO:0030647	aminoglycoside antibiotic metabolic process	0.006614403	0.0066144
GO:0070383	DNA cytosine deamination	0.006614403	0.0066144
GO:0044597	daunorubicin metabolic process	0.006614403	0.0066144
GO:0097193	intrinsic apoptotic signaling pathway	0.006614403	0.0066144
GO:0060561	apoptotic process involved in morphogenesis	0.006614403	0.0066144
GO:0070278	extracellular matrix constituent secretion	0.006614403	0.0066144
GO:0032103	positive regulation of response to external stimulus	0.006708023	0.006708
GO:0071356	cellular response to tumor necrosis factor	0.00737156	0.0073716
GO:0043065	positive regulation of apoptotic process	0.007883042	0.007883
GO:0046890	regulation of lipid biosynthetic process	0.007883042	0.007883
GO:0031327	negative regulation of cellular biosynthetic process	0.008116453	0.0081165
GO:0030638	polyketide metabolic process	0.008227763	0.0082278
GO:0010648	negative regulation of cell communication	0.008227763	0.0082278
GO:0044598	doxorubicin metabolic process	0.008227763	0.0082278
GO:0016554	cytidine to uridine editing	0.008227763	0.0082278
GO:0006950	response to stress	0.008227763	0.0082278
GO:0032880	regulation of protein localization	0.008227763	0.0082278
GO:0048856	anatomical structure development	0.008355755	0.0083558
GO:0097191	extrinsic apoptotic signaling pathway	0.008544215	0.0085442
GO:0044249	cellular biosynthetic process	0.008552028	0.008552
GO:0023057	negative regulation of signaling	0.008552028	0.008552
GO:0006954	inflammatory response	0.009069224	0.0090692
GO:0009890	negative regulation of biosynthetic process	0.009624718	0.0096247
GO:0043068	positive regulation of programmed cell death	0.009624718	0.0096247
GO:0002831	regulation of response to biotic stimulus	0.00989629	0.0098963
GO:0048523	negative regulation of cellular process	0.010235836	0.0102358
GO:0009893	positive regulation of metabolic process	0.010235836	0.0102358
GO:0030865	cortical cytoskeleton organization	0.010319334	0.0103193
GO:0002682	regulation of immune system process	0.010684546	0.0106845
GO:0010941	regulation of cell death	0.0108774	0.0108774
GO:1901576	organic substance biosynthetic process	0.010926992	0.010927
GO:0051896	regulation of protein kinase B signaling	0.010926992	0.010927
GO:0034612	response to tumor necrosis factor	0.010946178	0.0109462
GO:0032102	negative regulation of response to external stimulus	0.011229625	0.0112296
GO:0002468	dendritic cell antigen processing and presentation	0.012370175	0.0123702
GO:0019216	regulation of lipid metabolic process	0.013075598	0.0130756
GO:0048585	negative regulation of response to stimulus	0.013075598	0.0130756
GO:0045892	negative regulation of DNA-templated transcription	0.013571874	0.0135719
GO:0002484	antigen processing and presentation of endogenous peptide antigen via MHC class I via ER pathway	0.01392641	0.0139264
GO:0002486	antigen processing and presentation of endogenous peptide antigen via MHC class I via ER pathway, TAP-independent	0.01392641	0.0139264
GO:0071798	response to prostaglandin D	0.01392641	0.0139264
GO:1902679	negative regulation of RNA biosynthetic process	0.01392641	0.0139264
GO:1903507	negative regulation of nucleic acid-templated transcription	0.01392641	0.0139264
GO:0048869	cellular developmental process	0.01392641	0.0139264
GO:0071799	cellular response to prostaglandin D stimulus	0.01392641	0.0139264
GO:0045006	DNA deamination	0.01401026	0.0140103
GO:2000010	positive regulation of protein localization to cell surface	0.01401026	0.0140103
GO:1900025	negative regulation of substrate adhesion-dependent cell spreading	0.01401026	0.0140103
GO:0042448	progesterone metabolic process	0.01401026	0.0140103
GO:0010771	negative regulation of cell morphogenesis involved in differentiation	0.01401026	0.0140103
GO:0032879	regulation of localization	0.014084941	0.0140849
GO:0009058	biosynthetic process	0.014084941	0.0140849
GO:0045069	regulation of viral genome replication	0.014214363	0.0142144
GO:1901700	response to oxygen-containing compound	0.014264573	0.0142646
GO:0008207	C21-steroid hormone metabolic process	0.014714282	0.0147143
GO:0009649	entrainment of circadian clock	0.014714282	0.0147143

Supplemental Table S14. (Continued).

GO ID	GO Name	P Value	ADJ P Value
GO:0031341	regulation of cell killing	0.014714282	0.0147143
GO:0045732	positive regulation of protein catabolic process	0.014783883	0.0147839
GO:0046649	lymphocyte activation	0.014783883	0.0147839
GO:0019079	viral genome replication	0.014854681	0.0148547
GO:0050792	regulation of viral process	0.015553577	0.0155536
GO:0065007	biological regulation	0.015880386	0.0158804
GO:0002687	positive regulation of leukocyte migration	0.016099492	0.0160995
GO:0043542	endothelial cell migration	0.016558791	0.0165588
GO:0048519	negative regulation of biological process	0.01755302	0.017553
GO:0031349	positive regulation of defense response	0.017642697	0.0176427
GO:0019058	viral life cycle	0.017642697	0.0176427
GO:0030154	cell differentiation	0.017814671	0.0178147
GO:0043491	protein kinase B signaling	0.018152988	0.018153
GO:0045869	negative regulation of single stranded viral RNA replication via double stranded DNA intermediate	0.018723896	0.0187239
GO:0034694	response to prostaglandin	0.018723896	0.0187239
GO:0030036	actin cytoskeleton organization	0.019390353	0.0193904
GO:0051253	negative regulation of RNA metabolic process	0.019530455	0.0195305
GO:1904377	positive regulation of protein localization to cell periphery	0.019927046	0.019927
GO:0001775	cell activation	0.019927046	0.019927
GO:0050688	regulation of defense response to virus	0.019927046	0.019927
GO:0019882	antigen processing and presentation	0.020806217	0.0208062
GO:0031326	regulation of cellular biosynthetic process	0.020872326	0.0208723
GO:0061044	negative regulation of vascular wound healing	0.021001067	0.0210011
GO:0071072	negative regulation of phospholipid biosynthetic process	0.021001067	0.0210011
GO:0003332	negative regulation of extracellular matrix constituent secretion	0.021001067	0.0210011
GO:0010604	positive regulation of macromolecule metabolic process	0.021001067	0.0210011
GO:0010942	positive regulation of cell death	0.021646385	0.0216464
GO:1900744	regulation of p38MAPK cascade	0.024190946	0.0241909
GO:0060341	regulation of cellular localization	0.024190946	0.0241909
GO:0016137	glycoside metabolic process	0.024636699	0.0246367
GO:0006955	immune response	0.024636699	0.0246367
GO:0051173	positive regulation of nitrogen compound metabolic process	0.024636699	0.0246367
GO:0051701	biological process involved in interaction with host	0.025116865	0.0251169
GO:0050691	regulation of defense response to virus by host	0.025813005	0.025813
GO:0030029	actin filament-based process	0.025944795	0.0259448
GO:0009889	regulation of biosynthetic process	0.025944795	0.0259448
GO:0010558	negative regulation of macromolecule biosynthetic process	0.025944795	0.0259448
GO:0051897	positive regulation of protein kinase B signaling	0.025963166	0.0259632
GO:0045595	regulation of cell differentiation	0.025963166	0.0259632
GO:0009968	negative regulation of signal transduction	0.026066467	0.0260665
GO:1903900	regulation of viral life cycle	0.026153803	0.0261538
GO:0030155	regulation of cell adhesion	0.027008361	0.0270084
GO:0044403	biological process involved in symbiotic interaction	0.027008361	0.0270084
GO:0043652	engulfment of apoptotic cell	0.027008361	0.0270084
GO:0016553	base conversion or substitution editing	0.027008361	0.0270084
GO:0033036	macromolecule localization	0.027008361	0.0270084
GO:0002685	regulation of leukocyte migration	0.027008361	0.0270084
GO:0002684	positive regulation of immune system process	0.027008361	0.0270084
GO:0002483	antigen processing and presentation of endogenous peptide antigen	0.027008361	0.0270084
GO:0033993	response to lipid	0.027008361	0.0270084
GO:0043123	positive regulation of I-kappaB kinase/NF-kappaB signaling	0.027008361	0.0270084
GO:0050794	regulation of cellular process	0.027008361	0.0270084
GO:0009719	response to endogenous stimulus	0.027008361	0.0270084
GO:0045091	regulation of single stranded viral RNA replication via double stranded DNA intermediate	0.027008361	0.0270084
GO:0071222	cellular response to lipopolysaccharide	0.027268538	0.0272685
GO:0019222	regulation of metabolic process	0.027268538	0.0272685
GO:0043277	apoptotic cell clearance	0.027268538	0.0272685
GO:0051247	positive regulation of protein metabolic process	0.027268538	0.0272685
GO:0008610	lipid biosynthetic process	0.027268538	0.0272685
GO:0071675	regulation of mononuclear cell migration	0.028066202	0.0280662
GO:0051093	negative regulation of developmental process	0.028066202	0.0280662
GO:1903726	negative regulation of phospholipid metabolic process	0.028066202	0.0280662
GO:1904238	pericyte cell differentiation	0.028066202	0.0280662
GO:0001910	regulation of leukocyte mediated cytotoxicity	0.028579395	0.0285794
GO:0019218	regulation of steroid metabolic process	0.028579395	0.0285794

Supplemental Table S14. (Continued).

GO ID	GO Name	P Value	ADJ P Value
GO:0045934	negative regulation of nucleobase-containing compound metabolic process	0.028579395	0.0285794
GO:0039692	single stranded viral RNA replication via double stranded DNA intermediate	0.028579395	0.0285794
GO:0031324	negative regulation of cellular metabolic process	0.029203388	0.0292034
GO:0002697	regulation of immune effector process	0.030651865	0.0306519
GO:0045862	positive regulation of proteolysis	0.030651865	0.0306519
GO:0051234	establishment of localization	0.031842009	0.031842
GO:0019748	secondary metabolic process	0.033101255	0.0331013
GO:2000059	negative regulation of ubiquitin-dependent protein catabolic process	0.033101255	0.0331013
GO:2000630	positive regulation of miRNA metabolic process	0.033101255	0.0331013
GO:0002819	regulation of adaptive immune response	0.033137999	0.033138
GO:0045321	leukocyte activation	0.033186568	0.0331866
GO:1904375	regulation of protein localization to cell periphery	0.033464279	0.0334643
GO:0071496	cellular response to external stimulus	0.033909758	0.0339098
GO:0045444	fat cell differentiation	0.033909758	0.0339098
GO:1905475	regulation of protein localization to membrane	0.034848048	0.034848
GO:0071219	cellular response to molecule of bacterial origin	0.034848048	0.034848
GO:1902742	apoptotic process involved in development	0.0351558	0.0351558
GO:1901701	cellular response to oxygen-containing compound	0.0351558	0.0351558
GO:0098542	defense response to other organism	0.0351558	0.0351558
GO:2001141	regulation of RNA biosynthetic process	0.035397391	0.0353974
GO:0030178	negative regulation of Wnt signaling pathway	0.035397391	0.0353974
GO:0048260	positive regulation of receptor-mediated endocytosis	0.036870748	0.0368707
GO:1905897	regulation of response to endoplasmic reticulum stress	0.037418355	0.0374184
GO:0006629	lipid metabolic process	0.037771303	0.0377713
GO:0031328	positive regulation of cellular biosynthetic process	0.038812722	0.0388127
GO:0038066	p38MAPK cascade	0.038928765	0.0389288
GO:0045807	positive regulation of endocytosis	0.038928765	0.0389288
GO:0051179	localization	0.038928765	0.0389288
GO:0048513	animal organ development	0.039697904	0.0396979
GO:0062014	negative regulation of small molecule metabolic process	0.040705221	0.0407052
GO:0009059	macromolecule biosynthetic process	0.040837389	0.0408374
GO:0097435	supramolecular fiber organization	0.041494668	0.0414947
GO:0001667	ameboidal-type cell migration	0.041494668	0.0414947
GO:0001892	embryonic placenta development	0.042976289	0.0429763
GO:0022604	regulation of cell morphogenesis	0.043574098	0.0435741
GO:0030199	collagen fibril organization	0.043788574	0.0437886
GO:0001912	positive regulation of leukocyte mediated cytotoxicity	0.043788574	0.0437886
GO:0030162	regulation of proteolysis	0.043806228	0.0438062
GO:0050729	positive regulation of inflammatory response	0.04466043	0.0446604
GO:0051785	positive regulation of nuclear division	0.046435533	0.0464355
GO:0009891	positive regulation of biosynthetic process	0.046435533	0.0464355
GO:0043433	negative regulation of DNA-binding transcription factor activity	0.046827685	0.0468277
GO:0031652	positive regulation of heat generation	0.046840867	0.0468409
GO:0019883	antigen processing and presentation of endogenous antigen	0.046840867	0.0468409
GO:0010876	lipid localization	0.047285215	0.0472852
GO:0007586	digestion	0.047408175	0.0474082
GO:0016477	cell migration	0.04744546	0.0474455
GO:0051090	regulation of DNA-binding transcription factor activity	0.048491772	0.0484918
GO:0051246	regulation of protein metabolic process	0.048491772	0.0484918
GO:0090090	negative regulation of canonical Wnt signaling pathway	0.048491772	0.0484918
GO:1903506	regulation of nucleic acid-templated transcription	0.049386871	0.0493869

Supplemental Table S15. Enriched GO:BP categories for experimentally validated G4s overlapping enhancers, group 2.

GO ID	GO Name	P Value	ADJ P Value
GO:0002376	immune system process	4.15E-23	4.15E-23
GO:0006955	immune response	5.99E-23	5.99E-23
GO:0002682	regulation of immune system process	2.97E-21	2.97E-21
GO:0002684	positive regulation of immune system process	7.09E-18	7.09E-18
GO:0050776	regulation of immune response	1.08E-15	1.08E-15
GO:0002764	immune response-regulating signaling pathway	8.25E-15	8.25E-15
GO:0050778	positive regulation of immune response	1.77E-13	1.77E-13
GO:0002429	immune response-activating cell surface receptor signaling pathway	1.83E-13	1.83E-13
GO:0002757	immune response-activating signal transduction	1.83E-13	1.83E-13
GO:0002768	immune response-regulating cell surface receptor signaling pathway	8.77E-13	8.77E-13
GO:0002253	activation of immune response	1.39E-12	1.39E-12
GO:0046649	lymphocyte activation	1.75E-11	1.75E-11
GO:0045321	leukocyte activation	4.23E-11	4.23E-11
GO:0048584	positive regulation of response to stimulus	6.13E-11	6.13E-11
GO:0007165	signal transduction	6.13E-11	6.13E-11
GO:0002252	immune effector process	5.25E-10	5.25E-10
GO:0001819	positive regulation of cytokine production	1.28E-09	1.28E-09
GO:0001775	cell activation	1.28E-09	1.28E-09
GO:0002250	adaptive immune response	1.30E-09	1.30E-09
GO:0006952	defense response	3.61E-09	3.61E-09
GO:0050851	antigen receptor-mediated signaling pathway	4.06E-09	4.06E-09
GO:0023052	signaling	4.37E-09	4.37E-09
GO:0007154	cell communication	5.81E-09	5.81E-09
GO:0002697	regulation of immune effector process	6.28E-09	6.28E-09
GO:0050852	T cell receptor signaling pathway	9.32E-09	9.32E-09
GO:0007166	cell surface receptor signaling pathway	9.32E-09	9.32E-09
GO:0001817	regulation of cytokine production	9.32E-09	9.32E-09
GO:0001816	cytokine production	1.03E-08	1.03E-08
GO:0002700	regulation of production of molecular mediator of immune response	2.40E-08	2.40E-08
GO:0048583	regulation of response to stimulus	2.48E-08	2.48E-08
GO:0042110	T cell activation	2.73E-08	2.73E-08
GO:0032103	positive regulation of response to external stimulus	7.15E-08	7.15E-08
GO:0050896	response to stimulus	1.27E-07	1.27E-07
GO:0002440	production of molecular mediator of immune response	2.12E-07	2.12E-07
GO:0051716	cellular response to stimulus	2.31E-07	2.31E-07
GO:0002702	positive regulation of production of molecular mediator of immune response	2.55E-07	2.55E-07
GO:0002699	positive regulation of immune effector process	3.32E-07	3.32E-07
GO:1903131	mononuclear cell differentiation	4.15E-07	4.15E-07
GO:0032101	regulation of response to external stimulus	4.74E-07	4.74E-07
GO:0030098	lymphocyte differentiation	4.95E-07	4.95E-07
GO:0031347	regulation of defense response	7.94E-07	7.94E-07
GO:0046631	alpha-beta T cell activation	1.63E-06	1.63E-06
GO:0002521	leukocyte differentiation	1.65E-06	1.65E-06
GO:0070663	regulation of leukocyte proliferation	2.73E-06	2.73E-06
GO:0006954	inflammatory response	2.82E-06	2.82E-06
GO:0031663	lipopolysaccharide-mediated signaling pathway	3.23E-06	3.23E-06
GO:0046629	gamma-delta T cell activation	3.28E-06	3.28E-06
GO:0031349	positive regulation of defense response	3.75E-06	3.75E-06
GO:0010628	positive regulation of gene expression	4.15E-06	4.15E-06
GO:1903037	regulation of leukocyte cell-cell adhesion	4.15E-06	4.15E-06
GO:0043207	response to external biotic stimulus	4.62E-06	4.62E-06
GO:0051707	response to other organism	4.62E-06	4.62E-06
GO:0051240	positive regulation of multicellular organismal process	4.87E-06	4.87E-06
GO:0098542	defense response to other organism	5.73E-06	5.73E-06
GO:0002831	regulation of response to biotic stimulus	5.76E-06	5.76E-06
GO:0050670	regulation of lymphocyte proliferation	5.76E-06	5.76E-06
GO:0019221	cytokine-mediated signaling pathway	5.76E-06	5.76E-06
GO:0032944	regulation of mononuclear cell proliferation	6.38E-06	6.38E-06
GO:1903039	positive regulation of leukocyte cell-cell adhesion	7.12E-06	7.12E-06
GO:0002718	regulation of cytokine production involved in immune response	7.48E-06	7.48E-06
GO:0002367	cytokine production involved in immune response	7.48E-06	7.48E-06
GO:0009607	response to biotic stimulus	7.59E-06	7.59E-06
GO:0032755	positive regulation of interleukin-6 production	8.88E-06	8.88E-06
GO:0032735	positive regulation of interleukin-12 production	1.12E-05	1.12E-05
GO:0045785	positive regulation of cell adhesion	1.26E-05	1.26E-05
GO:0007159	leukocyte cell-cell adhesion	1.40E-05	1.40E-05

Supplemental Table S15. (Continued).

GO ID	GO Name	P Value	ADJ P Value
GO:0000165	MAPK cascade	1.41E-05	1.41E-05
GO:0032675	regulation of interleukin-6 production	1.41E-05	1.41E-05
GO:0032637	interleukin-8 production	1.41E-05	1.41E-05
GO:0032635	interleukin-6 production	1.41E-05	1.41E-05
GO:0032677	regulation of interleukin-8 production	1.41E-05	1.41E-05
GO:0070661	leukocyte proliferation	1.68E-05	1.68E-05
GO:0051249	regulation of lymphocyte activation	1.91E-05	1.91E-05
GO:0002833	positive regulation of response to biotic stimulus	2.18E-05	2.18E-05
GO:0032757	positive regulation of interleukin-8 production	2.52E-05	2.52E-05
GO:0044419	biological process involved in interspecies interaction between organisms	2.79E-05	2.79E-05
GO:0046651	lymphocyte proliferation	3.25E-05	3.25E-05
GO:0097530	granulocyte migration	3.40E-05	3.40E-05
GO:0022409	positive regulation of cell-cell adhesion	3.49E-05	3.49E-05
GO:0002221	pattern recognition receptor signaling pathway	3.55E-05	3.55E-05
GO:0032943	mononuclear cell proliferation	3.56E-05	3.56E-05
GO:0070371	ERK1 and ERK2 cascade	3.65E-05	3.65E-05
GO:0050900	leukocyte migration	4.13E-05	4.13E-05
GO:0071345	cellular response to cytokine stimulus	4.32E-05	4.32E-05
GO:0070374	positive regulation of ERK1 and ERK2 cascade	4.36E-05	4.36E-05
GO:0002520	immune system development	4.94E-05	4.94E-05
GO:0030097	hemopoiesis	5.42E-05	5.42E-05
GO:0009605	response to external stimulus	6.53E-05	6.53E-05
GO:0043410	positive regulation of MAPK cascade	6.53E-05	6.53E-05
GO:0002443	leukocyte mediated immunity	6.61E-05	6.61E-05
GO:0035556	intracellular signal transduction	6.61E-05	6.61E-05
GO:0050865	regulation of cell activation	6.67E-05	6.67E-05
GO:0048534	hematopoietic or lymphoid organ development	7.00E-05	7.00E-05
GO:1990266	neutrophil migration	7.20E-05	7.20E-05
GO:0022407	regulation of cell-cell adhesion	7.20E-05	7.20E-05
GO:0002220	innate immune response activating cell surface receptor signaling pathway	7.20E-05	7.20E-05
GO:0032615	interleukin-12 production	7.75E-05	7.75E-05
GO:0032655	regulation of interleukin-12 production	7.75E-05	7.75E-05
GO:0050863	regulation of T cell activation	8.28E-05	8.28E-05
GO:0002758	innate immune response-activating signal transduction	8.30E-05	8.30E-05
GO:0032760	positive regulation of tumor necrosis factor production	8.79E-05	8.79E-05
GO:0002694	regulation of leukocyte activation	9.15E-05	9.15E-05
GO:0050870	positive regulation of T cell activation	9.63E-05	9.63E-05
GO:0070372	regulation of ERK1 and ERK2 cascade	0.000101	0.000101363
GO:0002720	positive regulation of cytokine production involved in immune response	0.000114	0.000114437
GO:1903557	positive regulation of tumor necrosis factor superfamily cytokine production	0.000117	0.000116819
GO:0050764	regulation of phagocytosis	0.000125	0.000125399
GO:0070665	positive regulation of leukocyte proliferation	0.00014	0.000139526
GO:0043408	regulation of MAPK cascade	0.000161	0.000161131
GO:0034097	response to cytokine	0.000169	0.000169097
GO:0002460	adaptive immune response based on somatic recombination of immune receptors built from immunoglobulin superfamily domains	0.000176	0.000175603
GO:0009966	regulation of signal transduction	0.000178	0.000177725
GO:0002683	negative regulation of immune system process	0.000178	0.000178272
GO:0032680	regulation of tumor necrosis factor production	0.000188	0.000187589
GO:0032640	tumor necrosis factor production	0.000188	0.000187589
GO:0007249	I-kappaB kinase/NF-kappaB signaling	0.000188	0.000187589
GO:0042129	regulation of T cell proliferation	0.000196	0.000195896
GO:1902531	regulation of intracellular signal transduction	0.000202	0.000201588
GO:0050867	positive regulation of cell activation	0.000202	0.000201588
GO:0050766	positive regulation of phagocytosis	0.000204	0.000203772
GO:0002703	regulation of leukocyte mediated immunity	0.000204	0.000203772
GO:0030155	regulation of cell adhesion	0.000206	0.00020608
GO:0001818	negative regulation of cytokine production	0.000225	0.000225163
GO:0051251	positive regulation of lymphocyte activation	0.000225	0.000225163
GO:0051209	release of sequestered calcium ion into cytosol	0.000225	0.000225163
GO:0050727	regulation of inflammatory response	0.000225	0.000225163
GO:1903555	regulation of tumor necrosis factor superfamily cytokine production	0.000225	0.000225163
GO:0002639	positive regulation of immunoglobulin production	0.000225	0.000225163
GO:0071706	tumor necrosis factor superfamily cytokine production	0.000225	0.000225163
GO:0051283	negative regulation of sequestering of calcium ion	0.000234	0.000233805
GO:0051282	regulation of sequestering of calcium ion	0.000249	0.000248588

Supplemental Table S15. (Continued).

GO ID	GO Name	P Value	ADJ P Value
GO:0051235	maintenance of location	0.000289	0.000289196
GO:0051651	maintenance of location in cell	0.000293	0.000293015
GO:0051208	sequestering of calcium ion	0.000297	0.00029746
GO:0042098	T cell proliferation	0.000327	0.000326578
GO:0045059	positive thymic T cell selection	0.000327	0.000326578
GO:0002675	positive regulation of acute inflammatory response	0.000331	0.000330722
GO:0043122	regulation of I-kappaB kinase/NF-kappaB signaling	0.000348	0.000347721
GO:0097529	myeloid leukocyte migration	0.000414	0.000414075
GO:0002224	toll-like receptor signaling pathway	0.000415	0.000415086
GO:0050671	positive regulation of lymphocyte proliferation	0.000415	0.000415086
GO:0032602	chemokine production	0.000416	0.000416113
GO:0032642	regulation of chemokine production	0.000416	0.000416113
GO:0032946	positive regulation of mononuclear cell proliferation	0.000432	0.000432255
GO:0097553	calcium ion transmembrane import into cytosol	0.000435	0.000435396
GO:0048518	positive regulation of biological process	0.000435	0.000435396
GO:0030217	T cell differentiation	0.000462	0.000462365
GO:0043405	regulation of MAP kinase activity	0.000495	0.000495183
GO:0050794	regulation of cellular process	0.0005	0.000499699
GO:0002637	regulation of immunoglobulin production	0.000513	0.00051273
GO:0002696	positive regulation of leukocyte activation	0.000546	0.000546307
GO:0009617	response to bacterium	0.000588	0.000588485
GO:0032613	interleukin-10 production	0.000615	0.000614909
GO:0032653	regulation of interleukin-10 production	0.000615	0.000614909
GO:0010646	regulation of cell communication	0.000615	0.000614909
GO:0043368	positive T cell selection	0.000617	0.000617145
GO:0031664	regulation of lipopolysaccharide-mediated signaling pathway	0.000617	0.000617145
GO:0023051	regulation of signaling	0.000634	0.000633952
GO:0042102	positive regulation of T cell proliferation	0.000641	0.000640656
GO:0050729	positive regulation of inflammatory response	0.000648	0.000648482
GO:0032663	regulation of interleukin-2 production	0.000648	0.000648482
GO:0070383	DNA cytosine deamination	0.000648	0.000648482
GO:0032623	interleukin-2 production	0.000648	0.000648482
GO:0071310	cellular response to organic substance	0.000729	0.00072903
GO:0038093	Fc receptor signaling pathway	0.000782	0.000782428
GO:0002449	lymphocyte mediated immunity	0.000791	0.000790591
GO:0006909	phagocytosis	0.000838	0.000838027
GO:0045061	thymic T cell selection	0.000885	0.00088504
GO:0016554	cytidine to uridine editing	0.000885	0.00088504
GO:0071216	cellular response to biotic stimulus	0.000891	0.000891177
GO:0045089	positive regulation of innate immune response	0.000898	0.000898357
GO:0002879	positive regulation of acute inflammatory response to non-antigenic stimulus	0.000908	0.000907555
GO:0002426	immunoglobulin production in mucosal tissue	0.000908	0.000907555
GO:2000557	regulation of immunoglobulin production in mucosal tissue	0.000908	0.000907555
GO:0045087	innate immune response	0.000908	0.000907555
GO:2000558	positive regulation of immunoglobulin production in mucosal tissue	0.000908	0.000907555
GO:0002525	acute inflammatory response to non-antigenic stimulus	0.000908	0.000907555
GO:0033993	response to lipid	0.000908	0.000907555
GO:0002877	regulation of acute inflammatory response to non-antigenic stimulus	0.000908	0.000907555
GO:0071674	mononuclear cell migration	0.001082	0.001081991
GO:0009615	response to virus	0.001111	0.001111069
GO:0032722	positive regulation of chemokine production	0.001127	0.00112736
GO:0002685	regulation of leukocyte migration	0.001202	0.001201974
GO:0140546	defense response to symbiont	0.001215	0.001215436
GO:0051607	defense response to virus	0.001215	0.001215436
GO:0071396	cellular response to lipid	0.001236	0.001236267
GO:0006950	response to stress	0.001259	0.001259325
GO:0038094	Fc-gamma receptor signaling pathway	0.001263	0.001262965
GO:0030593	neutrophil chemotaxis	0.001263	0.001262965
GO:0045058	T cell selection	0.001263	0.001262965
GO:0032609	interferon-gamma production	0.001263	0.001262965
GO:0032649	regulation of interferon-gamma production	0.001263	0.001262965
GO:0002218	activation of innate immune response	0.001366	0.001366144
GO:0002532	production of molecular mediator involved in inflammatory response	0.001366	0.001366144
GO:0080134	regulation of response to stress	0.001407	0.001406934
GO:0045123	cellular extravasation	0.001468	0.001467829
GO:0071222	cellular response to lipopolysaccharide	0.001546	0.001546387

Supplemental Table S15. (Continued).

GO ID	GO Name	P Value	ADJ P Value
GO:1902533	positive regulation of intracellular signal transduction	0.001547	0.001547171
GO:0071677	positive regulation of mononuclear cell migration	0.001551	0.001551313
GO:0032733	positive regulation of interleukin-10 production	0.001551	0.001551313
GO:0002526	acute inflammatory response	0.001551	0.001551313
GO:0070588	calcium ion transmembrane transport	0.001602	0.001602101
GO:0045006	DNA deamination	0.001725	0.001724872
GO:0002673	regulation of acute inflammatory response	0.001728	0.001728104
GO:0051239	regulation of multicellular organismal process	0.001746	0.001746288
GO:0050789	regulation of biological process	0.001863	0.001862778
GO:0002819	regulation of adaptive immune response	0.002011	0.002010754
GO:0002705	positive regulation of leukocyte mediated immunity	0.002074	0.002074331
GO:0071219	cellular response to molecule of bacterial origin	0.00217	0.002170104
GO:0009967	positive regulation of signal transduction	0.002227	0.002226784
GO:0002238	response to molecule of fungal origin	0.002259	0.00225885
GO:0072676	lymphocyte migration	0.002259	0.00225885
GO:0030183	B cell differentiation	0.002259	0.00225885
GO:0071226	cellular response to molecule of fungal origin	0.002259	0.00225885
GO:0002237	response to molecule of bacterial origin	0.002327	0.002327333
GO:0045869	negative regulation of single stranded viral RNA replication via double stranded DNA intermediate	0.002534	0.002533743
GO:0045088	regulation of innate immune response	0.002534	0.002533743
GO:0002385	mucosal immune response	0.002534	0.002533743
GO:0033077	T cell differentiation in thymus	0.002534	0.002533743
GO:0045859	regulation of protein kinase activity	0.002698	0.002698387
GO:0006816	calcium ion transport	0.002721	0.002720968
GO:0031295	T cell costimulation	0.002776	0.002775759
GO:0002366	leukocyte activation involved in immune response	0.002848	0.002847835
GO:0002251	organ or tissue specific immune response	0.003046	0.003046143
GO:0071621	granulocyte chemotaxis	0.003053	0.003053229
GO:0002377	immunoglobulin production	0.003062	0.003062258
GO:0010033	response to organic substance	0.003271	0.003271259
GO:0002263	cell activation involved in immune response	0.003282	0.003282432
GO:0031294	lymphocyte costimulation	0.00329	0.00328994
GO:0007252	I-kappaB phosphorylation	0.003534	0.00353432
GO:0098609	cell-cell adhesion	0.003796	0.003796477
GO:0060326	cell chemotaxis	0.003911	0.003910682
GO:0002437	inflammatory response to antigenic stimulus	0.003911	0.003910682
GO:0043549	regulation of kinase activity	0.003975	0.003974909
GO:0016553	base conversion or substitution editing	0.004052	0.004051782
GO:0045091	regulation of single stranded viral RNA replication via double stranded DNA intermediate	0.004052	0.004051782
GO:0007186	G protein-coupled receptor signaling pathway	0.004052	0.004051782
GO:0002710	negative regulation of T cell mediated immunity	0.004052	0.004051782
GO:0033630	positive regulation of cell adhesion mediated by integrin	0.004052	0.004051782
GO:0071398	cellular response to fatty acid	0.004052	0.004051782
GO:0001954	positive regulation of cell-matrix adhesion	0.004143	0.004142522
GO:0002534	cytokine production involved in inflammatory response	0.004491	0.004490953
GO:1900015	regulation of cytokine production involved in inflammatory response	0.004491	0.004490953
GO:0039692	single stranded viral RNA replication via double stranded DNA intermediate	0.00464	0.004640311
GO:0002698	negative regulation of immune effector process	0.00464	0.004640311
GO:0050853	B cell receptor signaling pathway	0.00464	0.004640311
GO:0002719	negative regulation of cytokine production involved in immune response	0.00464	0.004640311
GO:1901222	regulation of NIK/NF-kappaB signaling	0.00486	0.00486036
GO:0014065	phosphatidylinositol 3-kinase signaling	0.005023	0.005022521
GO:0032651	regulation of interleukin-1 beta production	0.005085	0.005085136
GO:0032611	interleukin-1 beta production	0.005085	0.005085136
GO:0019722	calcium-mediated signaling	0.005259	0.005258622
GO:0006935	chemotaxis	0.00539	0.005390484
GO:0042330	taxis	0.00539	0.005390484
GO:0070887	cellular response to chemical stimulus	0.005912	0.005911985
GO:0030888	regulation of B cell proliferation	0.005959	0.005958639
GO:0002706	regulation of lymphocyte mediated immunity	0.006009	0.006008889
GO:0045577	regulation of B cell differentiation	0.006017	0.00601741
GO:2000523	regulation of T cell costimulation	0.006155	0.006154972
GO:0032496	response to lipopolysaccharide	0.006435	0.006435286
GO:0010811	positive regulation of cell-substrate adhesion	0.006435	0.006435286

Supplemental Table S15. (Continued).

GO ID	GO Name	P Value	ADJ P Value
GO:0010647	positive regulation of cell communication	0.006466	0.006465847
GO:0032703	negative regulation of interleukin-2 production	0.006743	0.006743232
GO:0002891	positive regulation of immunoglobulin mediated immune response	0.006743	0.006743232
GO:0002714	positive regulation of B cell mediated immunity	0.006743	0.006743232
GO:0023056	positive regulation of signaling	0.006762	0.006761765
GO:0050777	negative regulation of immune response	0.0068	0.006800008
GO:0048525	negative regulation of viral process	0.006959	0.006959196
GO:1903169	regulation of calcium ion transmembrane transport	0.007022	0.007021788
GO:0050854	regulation of antigen receptor-mediated signaling pathway	0.007121	0.007120548
GO:0032102	negative regulation of response to external stimulus	0.007489	0.007488582
GO:0006801	superoxide metabolic process	0.007609	0.007609318
GO:0051924	regulation of calcium ion transport	0.007691	0.007691448
GO:0014066	regulation of phosphatidylinositol 3-kinase signaling	0.007925	0.00792478
GO:0032652	regulation of interleukin-1 production	0.008266	0.008265842
GO:0032612	interleukin-1 production	0.008266	0.008265842
GO:0061099	negative regulation of protein tyrosine kinase activity	0.008378	0.008378341
GO:0050790	regulation of catalytic activity	0.008464	0.008464202
GO:0002692	negative regulation of cellular extravasation	0.008464	0.008464202
GO:1903721	positive regulation of I-kappaB phosphorylation	0.008464	0.008464202
GO:0033634	positive regulation of cell-cell adhesion mediated by integrin	0.008464	0.008464202
GO:0065007	biological regulation	0.00872	0.008720037
GO:0016064	immunoglobulin mediated immune response	0.00872	0.008720037
GO:0002832	negative regulation of response to biotic stimulus	0.008851	0.008851095
GO:0045071	negative regulation of viral genome replication	0.008978	0.008978332
GO:0002701	negative regulation of production of molecular mediator of immune response	0.009167	0.009166584
GO:0019724	B cell mediated immunity	0.009947	0.009947069
GO:0038096	Fc-gamma receptor signaling pathway involved in phagocytosis	0.010124	0.010123844
GO:0032743	positive regulation of interleukin-2 production	0.010124	0.010123844
GO:0050672	negative regulation of lymphocyte proliferation	0.010124	0.010123844
GO:0002433	immune response-regulating cell surface receptor signaling pathway involved in phagocytosis	0.010124	0.010123844
GO:0032945	negative regulation of mononuclear cell proliferation	0.010662	0.010661701
GO:0072678	T cell migration	0.010662	0.010661701
GO:0031666	positive regulation of lipopolysaccharide-mediated signaling pathway	0.010859	0.010859184
GO:0035701	hematopoietic stem cell migration	0.010859	0.010859184
GO:0002752	cell surface pattern recognition receptor signaling pathway	0.010859	0.010859184
GO:0045619	regulation of lymphocyte differentiation	0.010859	0.010859184
GO:0002732	positive regulation of dendritic cell cytokine production	0.010859	0.010859184
GO:2000272	negative regulation of signaling receptor activity	0.010859	0.010859184
GO:0010529	negative regulation of transposition	0.010859	0.010859184
GO:0010528	regulation of transposition	0.010859	0.010859184
GO:0070542	response to fatty acid	0.010859	0.010859184
GO:1903719	regulation of I-kappaB phosphorylation	0.010859	0.010859184
GO:0030595	leukocyte chemotaxis	0.010859	0.010859184
GO:0008284	positive regulation of cell population proliferation	0.010859	0.010859184
GO:0071675	regulation of mononuclear cell migration	0.011266	0.011265926
GO:0038061	NIK/NF-kappaB signaling	0.011266	0.011265926
GO:0042113	B cell activation	0.011266	0.011265926
GO:0039694	viral RNA genome replication	0.011833	0.011832838
GO:0050901	leukocyte tethering or rolling	0.011833	0.011832838
GO:0048015	phosphatidylinositol-mediated signaling	0.012113	0.012112633
GO:0043254	regulation of protein-containing complex assembly	0.012578	0.012577554
GO:0048017	inositol lipid-mediated signaling	0.012864	0.012863661
GO:2000406	positive regulation of T cell migration	0.012912	0.012911804
GO:0080111	DNA demethylation	0.012912	0.012911804
GO:0070664	negative regulation of leukocyte proliferation	0.013452	0.013451563
GO:0042221	response to chemical	0.013978	0.013977709
GO:0007204	positive regulation of cytosolic calcium ion concentration	0.013978	0.013977709
GO:0051279	regulation of release of sequestered calcium ion into cytosol	0.013993	0.013993161
GO:0150077	regulation of neuroinflammatory response	0.013993	0.013993161
GO:0032196	transposition	0.013993	0.013993161
GO:0032689	negative regulation of interferon-gamma production	0.013993	0.013993161
GO:0016477	cell migration	0.014138	0.014137848
GO:0072507	divalent inorganic cation homeostasis	0.014506	0.014505758
GO:0030890	positive regulation of B cell proliferation	0.015167	0.015166606
GO:0043507	positive regulation of JUN kinase activity	0.015167	0.015166606

Supplemental Table S15. (Continued).

GO ID	GO Name	P Value	ADJ P Value
GO:0061097	regulation of protein tyrosine kinase activity	0.015436	0.015435761
GO:1902105	regulation of leukocyte differentiation	0.015478	0.015477851
GO:0050864	regulation of B cell activation	0.015762	0.015762353
GO:0071356	cellular response to tumor necrosis factor	0.015762	0.015762353
GO:0051338	regulation of transferase activity	0.016043	0.016043375
GO:0045730	respiratory burst	0.01619	0.016189681
GO:0002431	Fc receptor mediated stimulatory signaling pathway	0.01619	0.016189681
GO:0042554	superoxide anion generation	0.01619	0.016189681
GO:0050850	positive regulation of calcium-mediated signaling	0.01619	0.016189681
GO:0006812	cation transport	0.016473	0.016473414
GO:0002725	negative regulation of T cell cytokine production	0.016473	0.016473414
GO:0002371	dendritic cell cytokine production	0.016473	0.016473414
GO:0001771	immunological synapse formation	0.016473	0.016473414
GO:0002730	regulation of dendritic cell cytokine production	0.016473	0.016473414
GO:2001187	positive regulation of CD8-positive, alpha-beta T cell activation	0.016473	0.016473414
GO:0002274	myeloid leukocyte activation	0.016676	0.01667619
GO:0051345	positive regulation of hydrolase activity	0.017032	0.017031812
GO:0006874	cellular calcium ion homeostasis	0.01719	0.017190273
GO:0035510	DNA dealkylation	0.01719	0.017190273
GO:0071900	regulation of protein serine/threonine kinase activity	0.018455	0.018455118
GO:0032715	negative regulation of interleukin-6 production	0.018634	0.018634454
GO:0046632	alpha-beta T cell differentiation	0.019694	0.01969355
GO:0002687	positive regulation of leukocyte migration	0.019694	0.01969355
GO:1905155	positive regulation of membrane invagination	0.019694	0.01969355
GO:0050856	regulation of T cell receptor signaling pathway	0.019694	0.01969355
GO:0034142	toll-like receptor 4 signaling pathway	0.019694	0.01969355
GO:0033632	regulation of cell-cell adhesion mediated by integrin	0.019694	0.01969355
GO:0060100	positive regulation of phagocytosis, engulfment	0.019694	0.01969355
GO:1902622	regulation of neutrophil migration	0.019694	0.01969355
GO:2000403	positive regulation of lymphocyte migration	0.019694	0.01969355
GO:0002381	immunoglobulin production involved in immunoglobulin-mediated immune response	0.019694	0.01969355
GO:0031348	negative regulation of defense response	0.020164	0.020163552
GO:1903706	regulation of hemopoiesis	0.020164	0.020163552
GO:0002823	negative regulation of adaptive immune response based on somatic recombination of immune receptors built from immunoglobulin superfamily domains	0.021179	0.021179088
GO:0010469	regulation of signaling receptor activity	0.021888	0.02188832
GO:0055074	calcium ion homeostasis	0.021888	0.02188832
GO:0034612	response to tumor necrosis factor	0.022143	0.022143164
GO:0048870	cell motility	0.022143	0.022143164
GO:0030101	natural killer cell activation	0.022143	0.022143164
GO:0002889	regulation of immunoglobulin mediated immune response	0.022536	0.022536238
GO:0042100	B cell proliferation	0.023001	0.023000802
GO:0034154	toll-like receptor 7 signaling pathway	0.023074	0.023073529
GO:0042116	macrophage activation	0.02398	0.023979626
GO:0002712	regulation of B cell mediated immunity	0.023996	0.023995791
GO:0002707	negative regulation of lymphocyte mediated immunity	0.023996	0.023995791
GO:0050871	positive regulation of B cell activation	0.024457	0.024457447
GO:2000404	regulation of T cell migration	0.025704	0.025703611
GO:0002822	regulation of adaptive immune response based on somatic recombination of immune receptors built from immunoglobulin superfamily domains	0.026016	0.026016257
GO:0018108	peptidyl-tyrosine phosphorylation	0.026467	0.026467035
GO:0034695	response to prostaglandin E	0.02664	0.026639653
GO:0060099	regulation of phagocytosis, engulfment	0.02664	0.026639653
GO:0071346	cellular response to interferon-gamma	0.02664	0.026639653
GO:1905153	regulation of membrane invagination	0.02664	0.026639653
GO:0001867	complement activation, lectin pathway	0.02664	0.026639653
GO:0001779	natural killer cell differentiation	0.02664	0.026639653
GO:0043277	apoptotic cell clearance	0.026973	0.026973093
GO:0018212	peptidyl-tyrosine modification	0.027082	0.027081775
GO:0043269	regulation of ion transport	0.027416	0.0274162
GO:0002695	negative regulation of leukocyte activation	0.027886	0.027885725
GO:0007155	cell adhesion	0.028279	0.028279444
GO:0046634	regulation of alpha-beta T cell activation	0.028279	0.028279444
GO:0009620	response to fungus	0.028279	0.028279444
GO:0002820	negative regulation of adaptive immune response	0.028279	0.028279444
GO:0033628	regulation of cell adhesion mediated by integrin	0.028279	0.028279444

Supplemental Table S15. (Continued).

GO ID	GO Name	P Value	ADJ P Value
GO:0045069	regulation of viral genome replication	0.028279	0.028279444
GO:0043124	negative regulation of I-kappaB kinase/NF-kappaB signaling	0.028279	0.028279444
GO:0019932	second-messenger-mediated signaling	0.028785	0.028784641
GO:1903900	regulation of viral life cycle	0.029052	0.029052059
GO:0002821	positive regulation of adaptive immune response	0.029229	0.029228647
GO:0031341	regulation of cell killing	0.029229	0.029228647
GO:2000010	positive regulation of protein localization to cell surface	0.029564	0.029563665
GO:1903428	positive regulation of reactive oxygen species biosynthetic process	0.029564	0.029563665
GO:0002281	macrophage activation involved in immune response	0.029564	0.029563665
GO:0002438	acute inflammatory response to antigenic stimulus	0.029564	0.029563665
GO:0150078	positive regulation of neuroinflammatory response	0.029564	0.029563665
GO:0031665	negative regulation of lipopolysaccharide-mediated signaling pathway	0.029564	0.029563665
GO:0061756	leukocyte adhesion to vascular endothelial cell	0.029564	0.029563665
GO:0002708	positive regulation of lymphocyte mediated immunity	0.029822	0.029821865
GO:0002456	T cell mediated immunity	0.029822	0.029821865
GO:0051090	regulation of DNA-binding transcription factor activity	0.030156	0.030156343
GO:0072503	cellular divalent inorganic cation homeostasis	0.0309	0.030900223
GO:0001932	regulation of protein phosphorylation	0.032852	0.032852444
GO:0062208	positive regulation of pattern recognition receptor signaling pathway	0.032901	0.032901349
GO:0043506	regulation of JUN kinase activity	0.032901	0.032901349
GO:0050730	regulation of peptidyl-tyrosine phosphorylation	0.033073	0.033073105
GO:0010820	positive regulation of T cell chemotaxis	0.033396	0.033396178
GO:0032725	positive regulation of granulocyte macrophage colony-stimulating factor production	0.033396	0.033396178
GO:0002283	neutrophil activation involved in immune response	0.033396	0.033396178
GO:0051403	stress-activated MAPK cascade	0.033557	0.033557327
GO:0002704	negative regulation of leukocyte mediated immunity	0.034456	0.034455812
GO:0050732	negative regulation of peptidyl-tyrosine phosphorylation	0.034456	0.034455812
GO:0043406	positive regulation of MAP kinase activity	0.035444	0.035443875
GO:0065009	regulation of molecular function	0.036371	0.036370684
GO:0042130	negative regulation of T cell proliferation	0.036381	0.036381229
GO:0031098	stress-activated protein kinase signaling cascade	0.036388	0.036387884
GO:1901701	cellular response to oxygen-containing compound	0.036632	0.036631881
GO:0034694	response to prostaglandin	0.037076	0.037075732
GO:0034116	positive regulation of heterotypic cell-cell adhesion	0.037076	0.037075732
GO:0010819	regulation of T cell chemotaxis	0.037076	0.037075732
GO:0034134	toll-like receptor 2 signaling pathway	0.037076	0.037075732
GO:0033631	cell-cell adhesion mediated by integrin	0.037076	0.037075732
GO:0040011	locomotion	0.037076	0.037075732
GO:0062207	regulation of pattern recognition receptor signaling pathway	0.038791	0.038791444
GO:0002285	lymphocyte activation involved in immune response	0.039305	0.039305427
GO:0032645	regulation of granulocyte macrophage colony-stimulating factor production	0.041185	0.041185212
GO:0061154	endothelial tube morphogenesis	0.041185	0.041185212
GO:0032604	granulocyte macrophage colony-stimulating factor production	0.041185	0.041185212
GO:0051092	positive regulation of NF-kappaB transcription factor activity	0.041185	0.041185212
GO:1900221	regulation of amyloid-beta clearance	0.041185	0.041185212
GO:0098655	cation transmembrane transport	0.041185	0.041185212
GO:0003159	morphogenesis of an endothelium	0.041185	0.041185212
GO:0050866	negative regulation of cell activation	0.041185	0.041185212
GO:0035746	granzyme A production	0.042275	0.04227481
GO:0002669	positive regulation of T cell anergy	0.042275	0.04227481
GO:0002325	natural killer cell differentiation involved in immune response	0.042275	0.04227481
GO:2000334	positive regulation of blood microparticle formation	0.042275	0.04227481
GO:0045584	negative regulation of cytotoxic T cell differentiation	0.042275	0.04227481
GO:0150129	positive regulation of interleukin-33 production	0.042275	0.04227481
GO:0072682	eosinophil extravasation	0.042275	0.04227481
GO:0035782	mature natural killer cell chemotaxis	0.042275	0.04227481
GO:2000332	regulation of blood microparticle formation	0.042275	0.04227481
GO:0097534	lymphoid lineage cell migration	0.042275	0.04227481
GO:0032826	regulation of natural killer cell differentiation involved in immune response	0.042275	0.04227481
GO:0002442	serotonin secretion involved in inflammatory response	0.042275	0.04227481
GO:0097535	lymphoid lineage cell migration into thymus	0.042275	0.04227481
GO:0042325	regulation of phosphorylation	0.042275	0.04227481
GO:0090721	primary adaptive immune response involving T cells and B cells	0.042275	0.04227481
GO:2000526	positive regulation of glycoprotein biosynthetic process involved in immunological synapse formation	0.042275	0.04227481
GO:0090720	primary adaptive immune response	0.042275	0.04227481

Supplemental Table S15. (Continued).

GO ID	GO Name	P Value	ADJ P Value
GO:0002554	serotonin secretion by platelet	0.042275	0.04227481
GO:2000513	positive regulation of granzyme A production	0.042275	0.04227481
GO:2000511	regulation of granzyme A production	0.042275	0.04227481
GO:2000517	regulation of T-helper 1 cell activation	0.042275	0.04227481
GO:0042543	protein N-linked glycosylation via arginine	0.042275	0.04227481
GO:0038123	toll-like receptor TLR1:TLR2 signaling pathway	0.042275	0.04227481
GO:0002913	positive regulation of lymphocyte anergy	0.042275	0.04227481
GO:2000420	negative regulation of eosinophil extravasation	0.042275	0.04227481
GO:2000419	regulation of eosinophil extravasation	0.042275	0.04227481
GO:0002439	chronic inflammatory response to antigenic stimulus	0.042275	0.04227481
GO:2000518	negative regulation of T-helper 1 cell activation	0.042275	0.04227481
GO:0014895	smooth muscle hypertrophy	0.042275	0.04227481
GO:0072564	blood microparticle formation	0.042275	0.04227481
GO:0048298	positive regulation of isotype switching to IgA isotypes	0.042275	0.04227481
GO:0002351	serotonin production involved in inflammatory response	0.042275	0.04227481
GO:0150127	regulation of interleukin-33 production	0.042275	0.04227481
GO:0072639	interleukin-33 production	0.042275	0.04227481
GO:0061048	negative regulation of branching involved in lung morphogenesis	0.042275	0.04227481
GO:1901700	response to oxygen-containing compound	0.042275	0.04227481
GO:0010604	positive regulation of macromolecule metabolic process	0.042333	0.042332873
GO:1900017	positive regulation of cytokine production involved in inflammatory response	0.042404	0.042404035
GO:0033623	regulation of integrin activation	0.042404	0.042404035
GO:0034162	toll-like receptor 9 signaling pathway	0.042404	0.042404035
GO:0046635	positive regulation of alpha-beta T cell activation	0.042404	0.042404035
GO:0032753	positive regulation of interleukin-4 production	0.042404	0.042404035
GO:0050792	regulation of viral process	0.042598	0.042597594
GO:0001952	regulation of cell-matrix adhesion	0.044537	0.044537257
GO:2001185	regulation of CD8-positive, alpha-beta T cell activation	0.046537	0.046537297
GO:2000379	positive regulation of reactive oxygen species metabolic process	0.046537	0.046537297
GO:0150076	neuroinflammatory response	0.046537	0.046537297
GO:1900225	regulation of NLRP3 inflammasome complex assembly	0.046537	0.046537297
GO:0046641	positive regulation of alpha-beta T cell proliferation	0.046537	0.046537297
GO:0043652	engulfment of apoptotic cell	0.046537	0.046537297
GO:0030050	vesicle transport along actin filament	0.046537	0.046537297
GO:0034341	response to interferon-gamma	0.046932	0.046932004
GO:0030001	metal ion transport	0.047045	0.04704543
GO:0051336	regulation of hydrolase activity	0.047944	0.047943669
GO:1903038	negative regulation of leukocyte cell-cell adhesion	0.048216	0.048216447
GO:0031343	positive regulation of cell killing	0.048216	0.048216447
GO:1904062	regulation of cation transmembrane transport	0.048851	0.048851314
GO:0042127	regulation of cell population proliferation	0.049172	0.04917162
